# A nerve injury–specific long noncoding RNA promotes neuropathic pain by increasing *Ccl2* expression

**DOI:** 10.1172/JCI153563

**Published:** 2022-07-01

**Authors:** Shibin Du, Shaogen Wu, Xiaozhou Feng, Bing Wang, Shangzhou Xia, Lingli Liang, Li Zhang, Gokulapriya Govindarajalu, Alexander Bunk, Feni Kadakia, Qingxiang Mao, Xinying Guo, Hui Zhao, Tolga Berkman, Tong Liu, Hong Li, Jordan Stillman, Alex Bekker, Steve Davidson, Yuan-Xiang Tao

**Affiliations:** 1 Department of Anesthesiology, New Jersey Medical School, Rutgers, The State University of New Jersey, Newark, New Jersey, USA.; 2 Department of Anesthesiology, Pain Research Center, and Neuroscience Graduate Program, University of Cincinnati College of Medicine, Cincinnati, Ohio, USA.; 3 Center for Advanced Proteomics Research, Department of Microbiology, Biochemistry & Molecular Genetics,; 4 Department of Pharmacology, Physiology & Neuroscience, and; 5 Department of Cell Biology & Molecular Medicine, New Jersey Medical School, Rutgers, The State University of New Jersey, Newark, New Jersey, USA.

**Keywords:** Cell Biology, Neuroscience, Epigenetics, Neurological disorders

## Abstract

Maladaptive changes of nerve injury–associated genes in dorsal root ganglia (DRGs) are critical for neuropathic pain genesis. Emerging evidence supports the role of long noncoding RNAs (lncRNAs) in regulating gene transcription. Here we identified a conserved lncRNA, named nerve injury–specific lncRNA (*NIS-lncRNA*) for its upregulation in injured DRGs exclusively in response to nerve injury. This upregulation was triggered by nerve injury–induced increase in DRG ELF1, a transcription factor that bound to the *NIS-lncRNA* promoter. Blocking this upregulation attenuated nerve injury–induced CCL2 increase in injured DRGs and nociceptive hypersensitivity during the development and maintenance periods of neuropathic pain. Mimicking *NIS-lncRNA* upregulation elevated CCL2 expression, increased CCL2-mediated excitability in DRG neurons, and produced neuropathic pain symptoms. Mechanistically, *NIS-lncRNA* recruited more binding of the RNA-interacting protein FUS to the *Ccl2* promoter and augmented *Ccl2* transcription in injured DRGs. Thus, *NIS-lncRNA* participates in neuropathic pain likely by promoting FUS-triggered DRG *Ccl2* expression and may be a potential target in neuropathic pain management.

## Introduction

Peripheral neuropathic pain caused by primary damage or injury to the peripheral nervous system is a complex and debilitating major health problem. It impacts the quality of life of over 4 million people with an estimated cost of $600 billion per year in neuropathic pain–associated health care and productivity losses in the United States (1). Current treatments for this disorder have only a modest effect and in a minority of neuropathic pain patients (2). One important reason for this inefficiency of treatment is the inability to target underlying mechanisms precisely. Neuropathic pain is characterized by spontaneous ongoing pain and persistent somatosensory hypersensitivity including allodynia and hyperalgesia. The development and maintenance of these pain hypersensitivities likely result from sensitization and abnormal ectopic discharge in neuromas at the sites of peripheral nerve injury and the primary sensory neurons of dorsal root ganglia (DRGs) (3, 4). Maladaptive changes in gene expression at the transcriptional and translational levels in DRGs following peripheral nerve injury participate in these abnormal firing activities (5–7). Understanding the molecular mechanisms of how these nerve injury–associated genes are altered in DRGs may enable us to develop a new strategy for neuropathic pain management.

C-C chemokine ligand 2 (CCL2), also called monocyte chemoattractant protein-1, is a small secreted protein that functions as an important proinflammatory mediator for the recruitment of immune cells to injured tissues (8), and plays a pivotal role in the peripheral mechanisms of neuropathic pain (9). *Ccl2* mRNA and CCL2 protein are expressed and upregulated in injured DRG neurons in several models of neuropathic pain (10–14). Like other pain-related peptides, CCL2 is stored in large dense core vesicles, is released in a calcium-dependent manner from DRG neuronal bodies and their terminals, and directly excites DRG neurons through activation of its preferred C-C motif receptor 2 (CCR2) by autocrine/paracrine processes (15–17). CCR2 activation sensitizes nociceptors via transactivation of transient receptor potential channels (15). Intrathecal injection of CCL2 produces rapid heat hyperalgesia and mechanical allodynia (17, 18). Intrathecal administration of CCL2-neutralizing antibodies or CCR2 antagonists alleviates mechanical allodynia in different neuropathic pain models (14, 17–19). Consistently, mice lacking CCR2 exhibit impaired mechanical allodynia after partial ligation of the sciatic nerve (20). Thus, CCL2 signaling among DRG neurons is likely an endogenous initiator of neuropathic pain. However, the mechanisms by which the *Ccl2* gene is upregulated in the DRG after peripheral nerve injury are unknown.

Long noncoding RNAs (lncRNAs) are longer than 200 nucleotides without protein-coding capability and participate in transcriptional and translational regulation of gene expression through their binding to proteins, DNAs, and other RNAs (21). Although many lncRNAs are dysregulated in the pain-associated regions after peripheral nerve injury (6, 7), the detailed mechanisms of how they contribute to neuropathic pain are still elusive. Here we report a native lncRNA in mammalian neurons. Because its expression in injured DRGs specifically responds to peripheral nerve injury, we named it nerve injury–specific lncRNA (*NIS-lncRNA*). We found that *NIS-lncRNA* contributes to the development and maintenance of neuropathic pain by promoting FUS-triggered transcriptional activation of *Ccl2* in DRG neurons. *NIS-lncRNA* is likely a biological instigator in neuropathic pain genesis.

## Results

### Identification of NIS-lncRNA.

**Figure 1:**
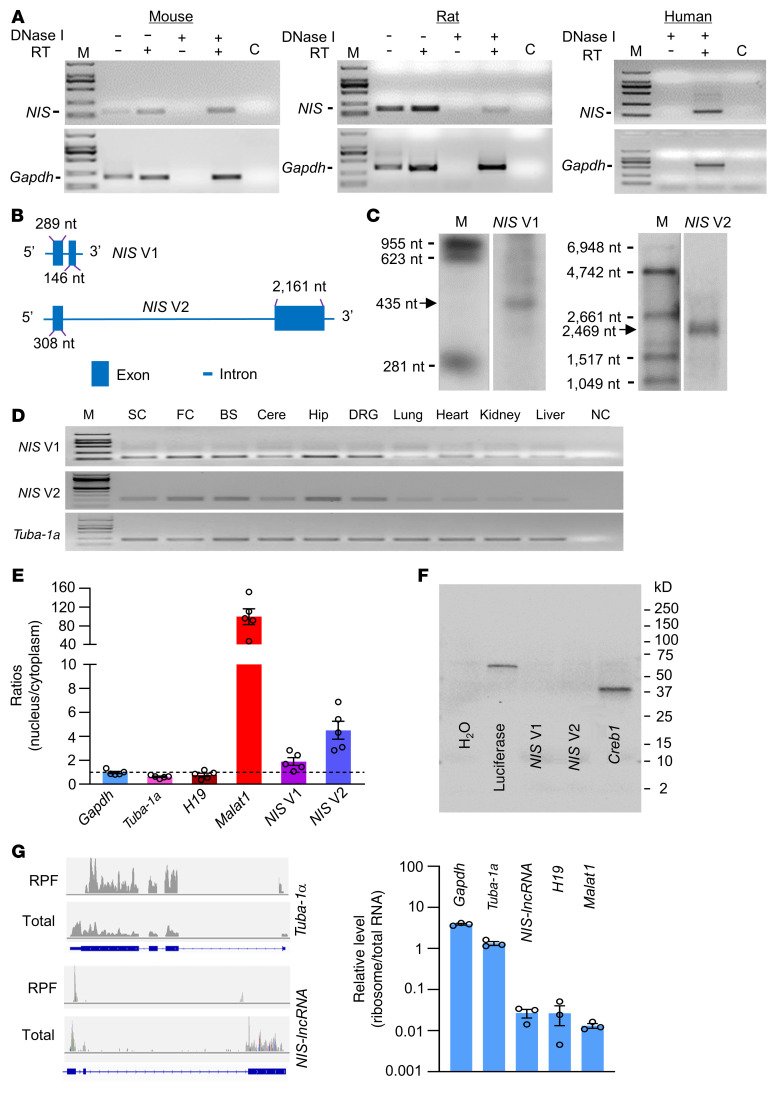
Identification of *NIS-lncRNA* in DRGs. (**A**) *NIS-lncRNA* transcript (*NIS*) in the lumbar DRGs of naive mouse, rat, and human using reverse transcriptase (RT) PCR with strand-specific primers. GAPDH is a control. Lane C: H_2_O. M: DNA ladder marker. *n =* 3 repeats per species. (**B**) Schematic diagrams of full-length *NIS* V1 and V2. (**C**) Northern blot expression analysis of *NIS* V1 and V2 (arrows) in the ipsilateral L4 DRG on day 7 after SNL. M: RNA marker. *n =* 3 repeats (10 mice per repeat). (**D**) *NIS* V1 and V2 transcripts in different tissues of normal mice. SC, spinal cord; FC, frontal cortex; BS, brainstem; Cere, cerebellum; Hip, hippocampus; NC, no-template control. *Tuba-1a* is a loading control. *n =* 3 mice. (**E**) Relative expression ratios of *Gapdh* mRNA, *Tuba-1a* mRNA, *H19*, *Malat1*, and *NIS* V1/V2 in nucleus versus cytoplasm from DRG. *n =* 5 mice. (**F**) In vitro translation of *NIS* V1 and V2 using the Promega Transcend Non-Radioactive Translation Detection Systems. Luciferase and *Creb1* are used as controls for coding RNA. *n =* 3 repeats. (**G**) Ribosome profiling of *NIS-lncRNA* and *Tuba-1a*. The blue rectangles represent their corresponding exons. Signal ratios of ribosome profiling to RNA sequencing (total) for mRNAs (*Gapdh* and *Tuba-1a*) and lncRNAs (*NIS-lncRNA*, *H19*, and *Malat1*). RPF, ribosome-protected fragments. *n =* 3 mice.

We first identified *NIS-lncRNA* through deep analysis of our previous RNA sequencing database (22) and found that the stacked reads in the genomic region (73,380,545–73,392,775) of chromosome 5 significantly increased in injured DRGs 7 days after unilateral L4 spinal nerve ligation (SNL) in comparison with sham-operated mice (Supplemental Figure 1; supplemental material available online with this article; https://doi.org/10.1172/JCI153563DS1). We then used strand-specific primers for reverse transcription and identified *NIS-lncRNA* transcripts in the DRGs of mice, rats, and humans (Figure 1A), although the sequences among these species were not completely identical. Using rapid amplification of cDNA ends for directional sequencing of 5′ and 3′ ends and reverse transcriptase PCR (RT-PCR) assays, we identified that *NIS-lncRNA* had 2 variants, full-length 435-nt variant 1 (*NIS* V1, including exons 1 and 2) and 2469-nt variant 2 (*NIS* V2, including exons 1 and 3), in mouse DRGs (Figure 1B and Supplemental Figure 2). A full-length 429-nt *NIS-lncRNA* (including exons 1 and 2) in rat DRGs (Supplemental Figure 3) and a full-length 1263-nt *NIS-lncRNA* in human DRGs (Supplemental Figure 4) were also identified. Using Northern blot analysis, *NIS* V1 and V2 were clearly confirmed at the expected size in injured mouse DRGs 7 days after SNL (Figure 1C), although detectable signals for both variants were extremely weak or undetected in normal/sham DRGs. The transcripts of both variants were also expressed in the spinal cord, various brain regions, and other body organs of naive mice (Figure 1D). Quantification analysis of nuclear/cytoplasmic RNA from DRG extracts showed that both variants were more enriched in the nucleus than in the cytoplasm (Figure 1E). Consistently, subcellular fractionation of DRGs revealed that *NIS* V1 and V2 displayed more distribution in the insoluble nuclear pellet (Supplemental Figure 5, A and B). Coding substitution frequency analysis proved no apparent open reading frame in both *NIS* V1 and V2 (Supplemental Figure 2). Furthermore, in vitro translation of these 2 variants did not yield proteins (Figure 1F). Ribosome profiling analysis showed no/minimal ribosomes on *NIS-lncRNA* (Figure 1G). The evidence indicates that *NIS-lncRNA* is a noncoding RNA.

### Upregulation of DRG NIS-lncRNA in specific response to peripheral nerve injury.

**Figure 2:**
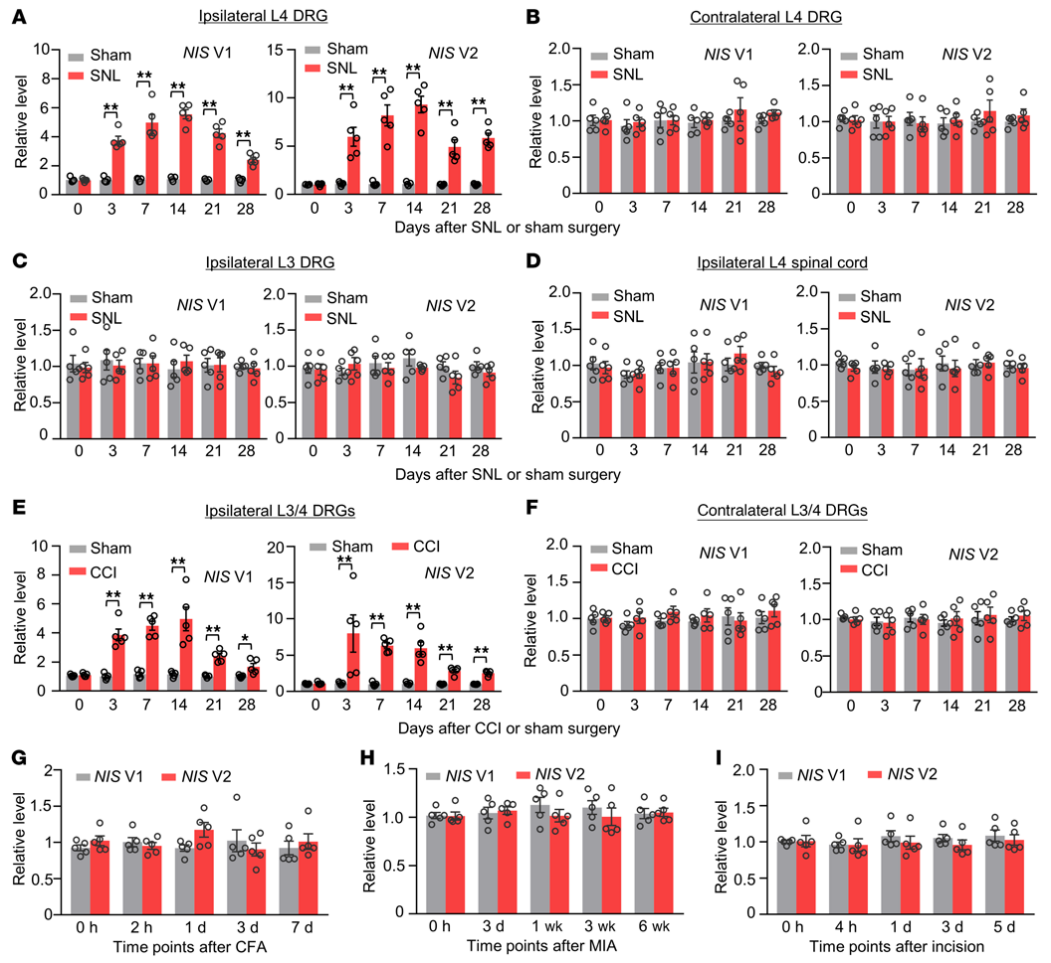
Upregulation of *NIS* V1 and V2 expression in injured DRGs of male mice after peripheral nerve injury. Data: mean ± SEM. (**A**–**D**) Levels of *NIS* V1 and V2 in the ipsilateral and contralateral L4 DRG, ipsilateral L3 DRG, and ipsilateral L4 spinal cord after SNL or sham surgery. *n =* 20 mice per time point per group. **P < 0.01, by 2-way ANOVA with post hoc Tukey’s test. (**E** and **F**) Levels of *NIS* V1 and V2 in the ipsilateral and contralateral L3/4 DRGs after CCI or sham surgery. *n =* 10 mice per time point per group. *P < 0.05, **P < 0.01, by 2-way ANOVA with post hoc Tukey’s test. (**G**–**I**) Levels of *NIS* V1 and V2 in the ipsilateral L3/4 DRGs after unilateral hind-paw injection of complete Freund’s adjuvant (CFA), after unilateral intra-articular injection of sodium monoiodoacetate (MIA), or after unilateral hind-paw incision. *n =* 10 mice per time point per group. One-way ANOVA with post hoc Tukey’s test.

Next, we carried out a quantitative RT-PCR assay plus an increasing sample template strategy and examined the *NIS-lncRNA* expression in 2 pain-associated regions, DRGs and spinal cord, after peripheral nerve injury. SNL, but not sham surgery, time-dependently upregulated both *NIS* V1 and V2 in the ipsilateral L4 DRG from day 3 to day 28 after surgery (Figure 2A). Neither SNL nor sham surgery altered basal expression of these 2 variants in the contralateral L4 DRG, ipsilateral intact L3 DRG, and ipsilateral L4 spinal cord (Figure 2, B–D). Results were similar in another animal model of neuropathic pain caused by chronic constriction injury (CCI) of unilateral sciatic nerve (Figure 2, E and F). Interestingly, levels of *NIS* V1 and V2 were not substantially changed in the ipsilateral L3/4 DRGs during the observation periods in animal models of chronic inflammatory pain caused by plantar injection of complete Freund’s adjuvant into the unilateral hind paw (Figure 2G) or intra-articular injection of sodium monoiodoacetate into the unilateral knee joint (Figure 2H) and in an animal model of postoperative pain caused by plantar incision of the unilateral hind paw (Figure 2I). Thus, it appears that transcriptional activation of DRG *NIS-lncRNA* occurs in specific response to peripheral nerve injury.

**Figure 3:**
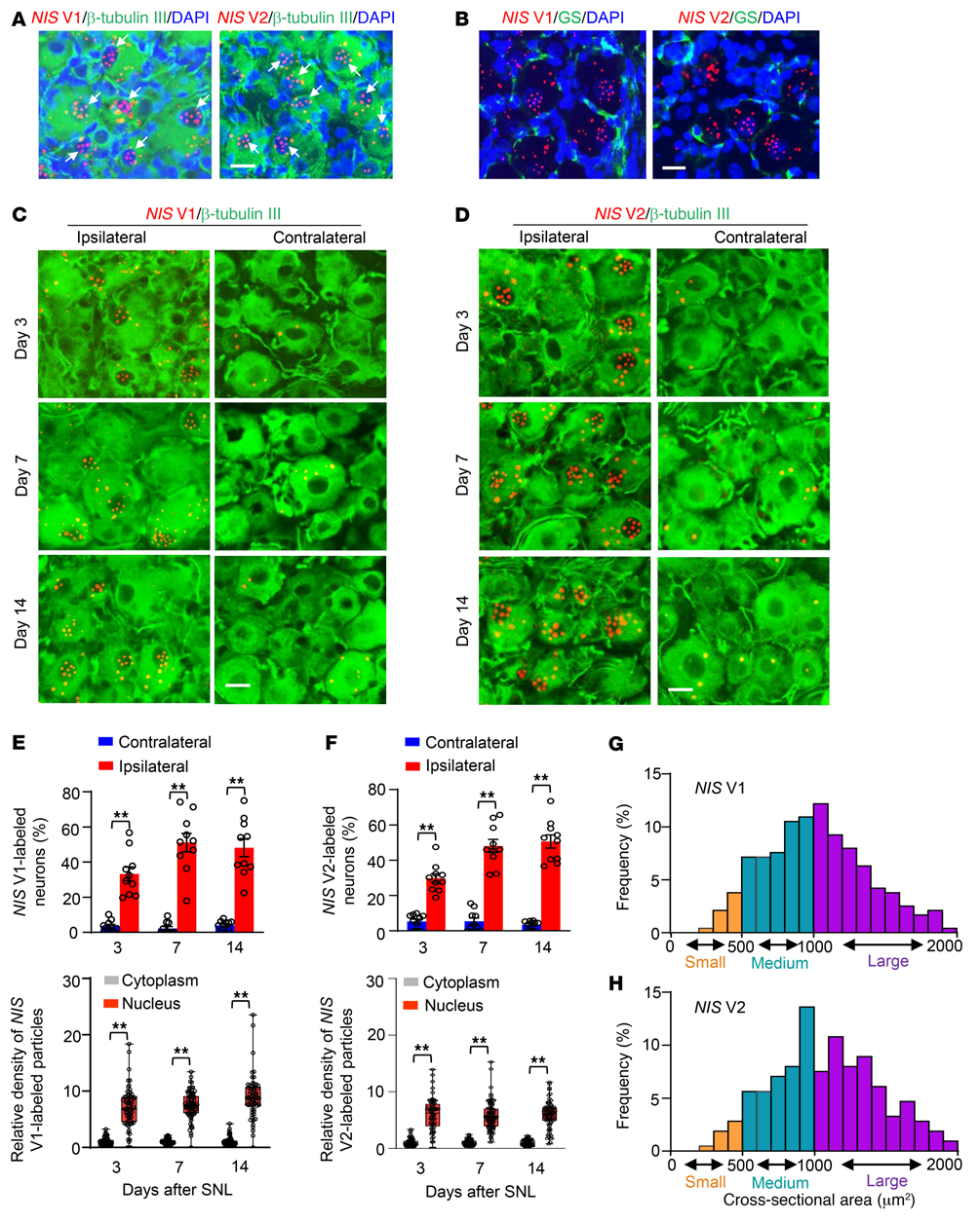
A significant increase in number of *NIS* V1– or V2–labeled neurons in injured DRGs of male mice after SNL. (**A** and **B**) Representative photomicrographs showing that *NIS* V1 (red, left) or V2 (red, right) was coexpressed with neuronal marker β-tubulin III (green) in the cells, but not detected in the satellite cells labeled by glutamine synthetase (GS, green), from the ipsilateral L4 DRG 7 days after SNL. Cellular nuclei are labeled by DAPI (blue). Arrows: signal particles (≥3) in neuronal nuclei. (**C**–**F**) Representative photomicrographs and corresponding statistical analysis. Relative densities of signal particles from 70, 76, and 67 DRG neurons for *NIS* V1 and 68, 82, and 73 DRG neurons for *NIS* V2 on days 3, 7, and 14 after SNL, respectively. Data: mean ± SEM. *n =* 5 mice per time point per group. **P < 0.01, by 2-way ANOVA with post hoc Tukey’s test. (**G** and **H**) Histograms showing the distribution of *NIS* V1– and V2–labeled somas in mouse ipsilateral L4 DRG on day 14 after SNL. *NIS* V1: small, 6%; medium, 43%; large, 51%. *NIS* V2: small, 5%; medium, 40%; large, 55%. *n =* 5 mice. Scale bars: 25 μm.

Cellular distribution pattern of *NIS-lncRNA* in the DRG was also observed using RNAscope in situ hybridization (ISH) assay followed by immunohistochemistry assay of distinct cell markers. The sensitivity and selectivity of the ISH assay were examined with a positive control probe for a housekeeping gene, peptidyl-prolyl *cis*-*trans* isomerase B (displaying robust signals), as well as a negative control probe for a specific bacterial gene, 4-hydroxy-tetrahydrodipicolinate (showing no signal), in the DRG (Supplemental Figure 6). *NIS* V1– or V2–labeled signal particles were undetected or sparsely detected in mouse DRGs under normal conditions (Supplemental Figure 7A) or after sham surgery (data not shown), but densely detected after SNL (Figure 3A). Both variants were coexpressed with β-tubulin III (a specific neuronal marker), but not glutamine synthetase (a marker for satellite glial cells) and CD68 (a marker for macrophages and monocytes), in individual cells of injured DRGs (Figure 3, A and B, and Supplemental Figure 7B). Quantification analysis showed that numbers of *NIS* V1– or V2–labeled neurons in the ipsilateral L4 DRG were significantly higher than those in the contralateral L4 DRG on days 3, 7, and 14 after SNL (Figure 3, C–F). Importantly, *NIS* V1– or V2–labeled signal particles (≥3) were more enriched in the nucleus than in the cytoplasm within the neurons from injured DRGs on the days indicated above after SNL (Figure 3, C–F, and Supplemental Figure 7C). A cross-sectional area analysis of neuronal somas showed that these changes occurred predominantly in medium and large DRG neurons from SNL mice (Figure 3, G and H). The majority of *NIS* V1– or V2–labeled neurons were positive for activating transcription factor 3 (an injury marker) in injured DRGs (Supplemental Figure 8). Consistently, in uninjured human DRGs, *NIS-lncRNA* was undetectable by RNAscope ISH assay (data not shown). We carried out a single-cell RT-PCR assay and revealed that about 86.7% of small neurons and 69.2% of large neurons expressed *NIS-lncRNA* in uninjured human DRGs (Supplemental Figure 9). Taken together, the evidence suggests that nerve injury–induced *NIS-lncRNA* upregulation in injured DRG neurons may have a functional role in neuropathic pain.

### ELF1 triggers DRG NIS-lncRNA transcriptional activity after SNL.

**Figure 4:**
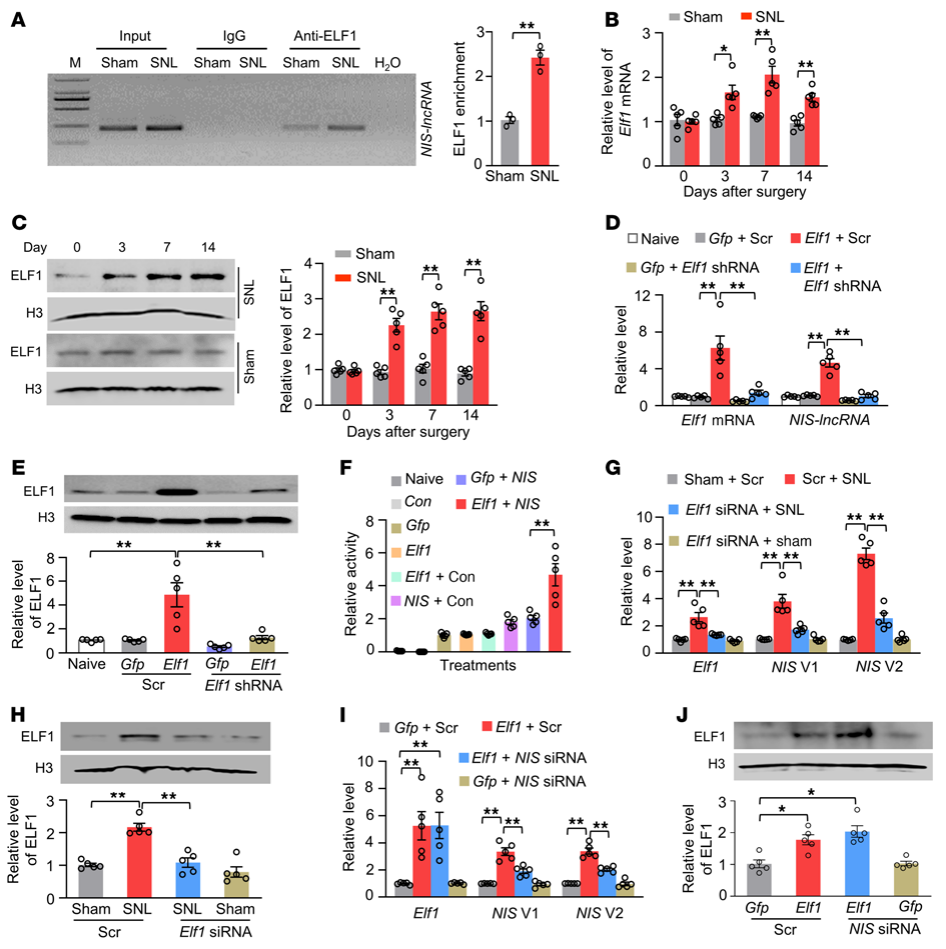
ELF1 triggers *NIS-lncRNA* upregulation in injured DRGs following SNL. Data: mean ± SEM. (**A**) *NIS-lncRNA* gene promoter fragment immunoprecipitated by rabbit anti-ELF1 antibody in the ipsilateral L4 DRG 7 days after surgery. *n =* 36 mice per group. **P < 0.01, by 2-tailed, unpaired Student’s *t* test. (**B** and **C**) Levels of *Elf1* mRNA and ELF1 protein in the ipsilateral L4 DRG after surgery. *n =* 20 mice per time point per group. *P < 0.05, **P < 0.01, by 2-way ANOVA with post hoc Tukey’s test. (**D** and **E**) Levels of *Elf1* mRNA, *NIS-lncRNA*), and ELF1 protein in cultured DRG neurons with transfection as indicated. *Elf1*: AAV5-*Elf1*; *Gfp*: AAV5-*Gfp*; Scr: scrambled shRNA. *n =* 5 repeats per treatment. **P < 0.01, by 1-way ANOVA with post hoc Tukey’s test. (**F**) *NIS-lncRNA* promoter activities in CAD cells transfected as shown. Con: control vector; *Elf1*: vector expressing *Elf1*; *Gfp*, vector expressing *Gfp*; *NIS*: pGL3 reporter vector with *NIS-lncRNA* promoter. *n =* 5 repeats per treatment. **P < 0.01, by 1-way ANOVA with post hoc Tukey’s test. (**G** and **H**) Levels of *Elf1* mRNA, *NIS* V1, and *NIS* V2 and ELF1 protein in the ipsilateral L4 DRG 5 days after surgery in mice with microinjection of scrambled siRNA (Scr) or *Elf1* siRNA 3 days before surgery. *n =* 20 mice per group. **P < 0.01, by 2-way ANOVA with post hoc Tukey’s test. (**I** and **J**) Levels of *Elf1* mRNA, *NIS* V1 and V2, and ELF1 protein in the ipsilateral L3/4 DRGs 7 days after DRG microinjection of *NIS-lncRNA* siRNA (*NIS* siRNA) or scrambled siRNA (Scr) in mice with microinjection of AAV5-*Gfp* (*Gfp*) or AAV5-*Elf1* (*Elf1*) 35 days before siRNA microinjection. *n =* 10 mice per group. *P < 0.05, **P < 0.01, by 1-way ANOVA with post hoc Tukey’s test.

We examined how DRG *NIS-lncRNA* was upregulated after nerve injury. Given that transcription factors regulate gene expression, we used the online database JASPAR and identified a consensus binding motif (_–227_TGGGGAGGAAGTT_–215_) for E74-like ETS transcription factor 1 (ELF1) in the promoter region of *NIS-lncRNA*. A fragment of the *NIS-lncRNA* promoter containing this binding motif could be amplified from the complex immunoprecipitated with ELF1 antibody in nuclear fractions from sham DRGs (Figure 4A). SNL increased the binding of ELF1 to the *NIS-lncRNA* promoter, as demonstrated by a 2.4-fold increase in binding activity in the ipsilateral L4 DRG on day 7 after SNL (Figure 4A). This increase was due to the elevations in the levels of *Elf1* mRNA and ELF1 protein in the ipsilateral L4 DRG following SNL, not sham surgery (Figure 4, B and C).

We further observed an ELF1 overexpression–induced increase of *NIS-lncRNA* in in vitro–cultured DRG neurons transduced with AAV5 expressing the full-length *Elf1* mRNA (AAV5-*Elf1*) plus AAV5 expressing control scrambled shRNA (Figure 4, D and E). This increase was absent in the DRG neurons transduced with AAV5-*Elf1* plus AAV5 expressing *Elf1* shRNA (AAV5-*Elf1* shRNA) (Figure 4, D and E), suggesting that the *NIS-lncRNA* increase was in specific response to ELF1. The luciferase assay revealed that cotransfection of full-length *Elf1* vector, but not control *Gfp* vector, significantly increased the activity of the *NIS-lncRNA* promoter in in vitro CAD cells (Figure 4F). Furthermore, blocking the SNL-induced increase of DRG ELF1 through DRG microinjection of *Elf1* siRNA (but not control scrambled siRNA) 3 days before surgery not only mitigated the development of SNL-induced mechanical, heat, and cold nociceptive hypersensitivities in male mice (Supplemental Figure 10, A–D), but also attenuated the SNL-induced increases in the levels of *NIS* V1 and V2 in the ipsilateral L4 DRG (Figure 4, G and H). In addition, DRG microinjection of AAV5-*Elf1*, but not AAV5-*Gfp*, in the scrambled siRNA–microinjected mice 35 days before siRNA microinjection increased the amounts of *NIS* V1 and V2 in the injected L3/4 DRGs on day 7 after siRNA microinjection and enhanced responses to mechanical, heat, and cold stimuli on the ipsilateral side on days 5 and 7 after siRNA microinjection in male mice (Figure 4, I and J, and Supplemental Figure 11, A–D). These changes were significantly attenuated in the mice co-microinjected with *NIS-lncRNA* siRNA and AAV5-*Elf1* (Figure 4, I and J, and Supplemental Figure 10, A–D). As expected, neither siRNA nor virus changed basal paw withdrawal responses on the contralateral side (Supplemental Figure 10, E–G, and Supplemental Figure 11, E–G) or locomotor function (Supplemental Table 1). Given that *Elf1* mRNA was coexpressed with *NIS* V1 and V2 in individual small, medium, and large DRG neurons (Supplemental Figure 12), our findings suggest that nerve injury–induced *NIS-lncRNA* upregulation is attributable, at least in part, to an increase of ELF1 expression in injured DRGs.

### Blocking upregulated DRG NIS-lncRNA alleviates neuropathic pain.

**Figure 5:**
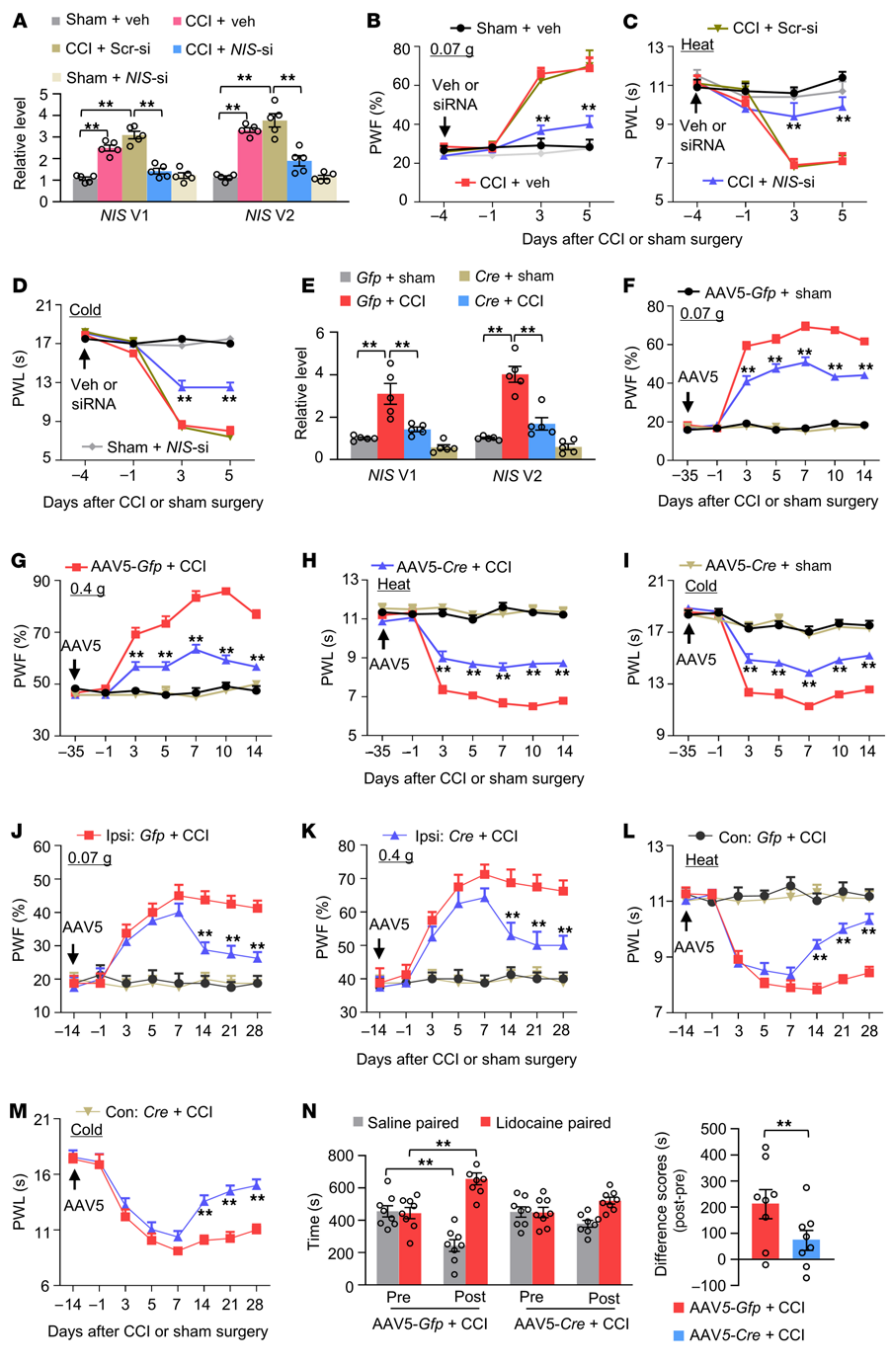
Blocking DRG *NIS-lncRNA* upregulation mitigates neuropathic pain development and maintenance in male mice. Data: mean ± SEM. PWF, paw withdrawal frequency; PWL, paw withdrawal latency. (**A**) Levels of *NIS* V1 and V2 in the ipsilateral L3/4 DRGs 5 days after surgery in mice with microinjection of *NIS-lncRNA* siRNA (*NIS*-si), scrambled siRNA (Scr-si), or vehicle (Veh) into unilateral L3/4 DRGs 4 days before surgery. *n =* 10 mice per group. **P < 0.01, by 2-way ANOVA with post hoc Tukey’s test. (**B**–**D**) Effect of microinjection of *NIS-lncRNA* siRNA, scrambled siRNA, or vehicle on the development of CCI-induced nociceptive hypersensitivity. *n =* 10 mice per group. **P < 0.01 vs. CCI plus vehicle group at the corresponding time points by 3-way ANOVA with repeated measures followed by post hoc Tukey’s test. (**E**) Levels of *NIS* V1 and V2 in the ipsilateral L3/4 DRGs 14 days after surgery in *NIS-lncRNA^fl/fl^* mice with DRG microinjection of AAV5-*Gfp* or AAV5-*Cre* 35 days before surgery. *n =* 10 mice per group. **P < 0.01, by 2-way ANOVA with post hoc Tukey’s test. (**F**–**M**) Effect of microinjection of AAV5-*Gfp* or AAV5-*Cre* into the ipsilateral L3/4 DRGs of *NIS-lncRNA^fl/fl^* mice on the development (**F**–**I**) and maintenance (**J**–**M**) of CCI-induced nociceptive hypersensitivity. *n =* 8–12 mice per group. **P < 0.01 vs. AAV5-*Gfp* plus CCI mice on the ipsilateral side at the corresponding time points by 3-way ANOVA with repeated measures followed by post hoc Tukey’s test. (**N**) Effect of microinjection of AAV5-*Cre* or AAV5-*Gfp* into ipsilateral L3/4 DRGs of *NIS-lncRNA^fl/fl^* mice on spontaneous ongoing pain as assessed by the conditional place preference paradigm 4 weeks after surgery. *n =* 8 mice per group. **P < 0.01, by 3-way ANOVA with post hoc Tukey’s test (left) or 2-tailed, unpaired Student’s *t* test (right).

Does the upregulated *NIS-lncRNA* in injured DRGs contribute to neuropathic pain? To this end, we first examined whether blocking DRG *NIS-lncRNA* upregulation affected the development of nerve injury–induced nociceptive hypersensitivity. *NIS-lncRNA* siRNA that significantly knocked down *NIS-lncRNA* and its 2 variants in in vitro–cultured DRG neurons (Supplemental Figure 13A) was microinjected into unilateral L3/4 DRGs 4 days before CCI or sham surgery in male mice. The CCI (not SNL) model was chosen because the mice were subjected to 2 surgeries at a 4-day interval. This microinjection not only blocked the CCI-induced increase in the levels of *NIS* V1 and V2 in the ipsilateral L3/4 DRGs on day 5 after CCI (Figure 5A) but also ameliorated the CCI-induced mechanical, heat, and cold nociceptive hypersensitivities on the ipsilateral side on days 3 and 5 after CCI (Figure 5, B–D). Microinjection of the control scrambled siRNA did not display these effects (Figure 5, B–D). After DRG microinjection of either siRNA, no changes were observed in basal levels of *NIS* V1 and V2 in the ipsilateral L3/4 DRGs of sham-operated mice, nor in basal mechanical, heat, or cold responses on the contralateral side of CCI mice and on both sides of sham-operated mice (Figure 5, A–D, and Supplemental Figure 13, B and C). Results were similar in CCI male mice microinjected with specific *NIS* V1 siRNA or V2 siRNA 4 days before surgery (Supplemental Figure 14, A–L). Locomotor function was normal in these treated mice (Supplemental Table 1).

siRNA may have potential off-target effects. To further confirm the role of DRG *NIS-lncRNA* in neuropathic pain, we generated *NIS-lncRNA^fl/fl^* mice (Supplemental Figure 15) and found that microinjection of AAV5-*Cre*, but not AAV5-*Gfp*, into the ipsilateral L3/4 DRGs of male mice 35 days before surgery blocked the CCI-induced increases in the levels of *NIS* V1 and V2 in the ipsilateral L3/4 DRGs 14 days after CCI (Figure 5E). This microinjection also mitigated development of the CCI-induced mechanical, heat, and cold nociceptive hypersensitivities on the ipsilateral side from day 3 to day 14 after CCI (Figure 5, F–I). Basal paw withdrawal responses on the ipsilateral side of sham *NIS-lncRNA^fl/fl^* mice with DRG microinjection of AAV5-*Cre* were not altered during the observation period (Figure 5, F–I), although there was a tendency toward decreased basal levels of *NIS* V1 and V2 in the ipsilateral L3/4 DRGs of these mice (Figure 5E). The role of DRG *NIS-lncRNA* upregulation in the maintenance of neuropathic pain was also examined. Given that AAV5-*Cre* requires 3–4 weeks to become expressed (23, 24), *NIS-lncRNA^fl/fl^* male mice were subjected to CCI 14 days after DRG viral microinjection. As expected, mechanical, heat, and cold nociceptive hypersensitivities on the ipsilateral side were completely developed in AAV5-*Cre*–microinjected *NIS-lncRNA^fl/fl^* mice on day 7 after CCI (Figure 5, J–M). These nociceptive hypersensitivities were significantly attenuated from day 14 to day 28 after CCI (Figure 5, J–M). Moreover, stimulation-independent spontaneous ongoing pain 28 days after CCI was also markedly diminished in the AAV5-*Cre*–microinjected *NIS-lncRNA^fl/fl^* mice (Figure 5N). Similar results were observed in CCI female and SNL male *NIS-lncRNA^fl/fl^* mice with DRG AAV5 microinjection (Supplemental Figure 16, A–H, and Supplemental Figure 17, A–I). Additionally, DRG microinjection of AAV5-*Cre* into the ipsilateral L4 DRG of *NIS-lncRNA^fl/fl^* mice reduced SNL-induced dorsal horn neuronal/glial hyperactivities as indicated by the abolition of SNL-induced increases in the phosphorylation of extracellular signal–regulated kinase 1 and 2 (p-ERK1/2, a marker for neuronal hyperactivation) and glial fibrillary acidic protein (GFAP, a marker of astrocyte hyperactivation) in the ipsilateral L4 dorsal horn 14 days after SNL (Supplemental Figure 17J).

DRG microinjection may lead to cell damage, although the injected DRG retained its structural integrity and exhibited no changes in the number of cells (23, 24). To exclude the possibility that the behavioral effects observed above were caused by tissue damage, we examined SNL-induced nociceptive hypersensitivities in male mice with conditional *NIS-lncRNA* knockdown (*NIS* KD), which were generated by cross-breeding of *NIS-lncRNA^fl/fl^* female mice with sensory neuron–specific *Advillin^Cre/+^* male mice. Like AAV5-*Cre*–injected mice, *NIS-*KD mice displayed the attenuation in the SNL-induced increases of *NIS* V1 and V2 in the ipsilateral L4 DRG 14 days after SNL and the impaired mechanical, heat, and cold nociceptive hypersensitivities on the ipsilateral side from day 3 to day 14 after SNL (Supplemental Figure 18, A–E). Basal responses on the contralateral side of SNL/CCI mice and on both sides of sham-operated mice were not altered in the virus-microinjected mice or genetic-KD mice (Figure 5, F–M, and Supplemental Figure 18, A–K). All microinjected/KD mice exhibited normal locomotor activity (Supplemental Table 1).

Taken together, our findings strongly suggest that upregulated *NIS-lncRNA* in injured DRGs is required for neuropathic pain development and maintenance.

### Mimicking nerve injury–induced DRG NIS-lncRNA upregulation produces nociceptive hypersensitivity.

**Figure 6:**
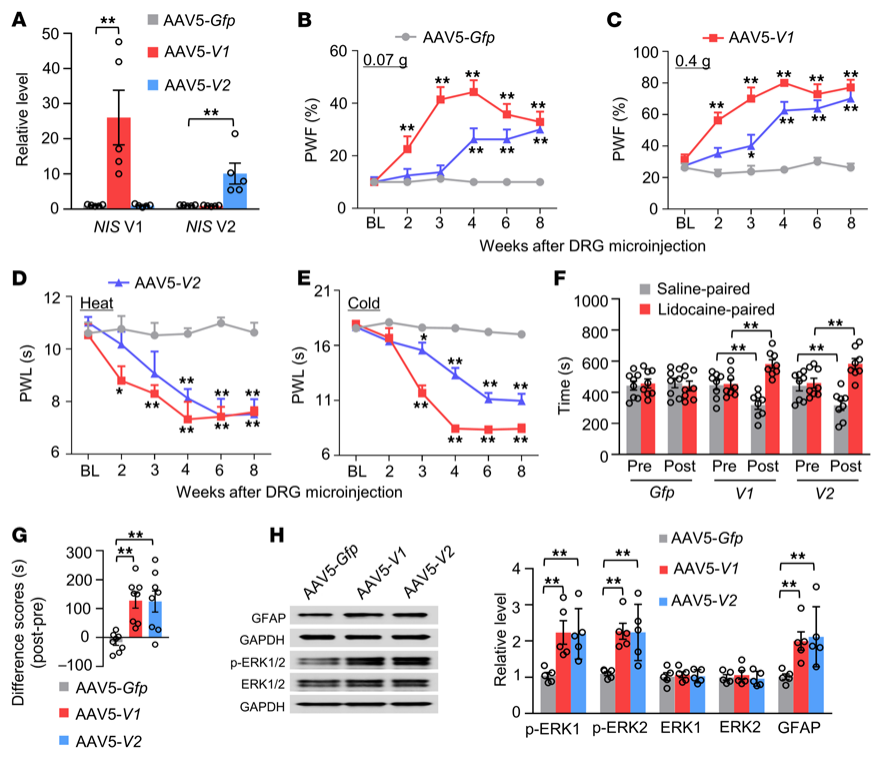
DRG overexpression of *NIS-lncRNA* produces neuropathic pain–like symptoms in naive male mice. Data: mean ± SEM. (**A**) Levels of *NIS* V1 and V2 in the ipsilateral L3/4 DRGs 8 weeks after microinjection of AAV5-*Gfp*, AAV5–*NIS* V1 (AAV5-*V1*), or AAV5–*NIS* V2 (AAV5-*V2*) into unilateral L3/4 DRGs. *n =* 10 mice per group. **P < 0.01, by 1-way ANOVA with post hoc Tukey’s test. (**B**–**E**) Effect of microinjection of AAV5-*V1*, AAV5-*V2*, or AAV5-*Gfp* into the ipsilateral L3/4 DRGs on the ipsilateral paw withdrawal frequency (PWF) in response to 0.07 g and 0.4 g von Frey filament stimuli and paw withdrawal latency (PWL) in response to heat and cold stimuli at indicated weeks after microinjection. *n =* 8 mice per group. *P < 0.05, **P < 0.01 vs. AAV5-*Gfp*–treated mice at the corresponding weeks by 2-way ANOVA with repeated measures followed by post hoc Tukey’s test. (**F** and **G**) Effect of microinjection of AAV5-*V1*, AAV5-*V2*, or AAV5-*Gfp* into the ipsilateral L3/4 DRGs on spontaneous ongoing pain as assessed by the conditional place preference paradigm 8 weeks after microinjection. Pre, preconditioning; Post, post-conditioning. *n =* 8 mice per group. **P < 0.01, by 2-way (**F**) or 1-way (**G**) ANOVA with repeated measures followed by post hoc Tukey’s test. (**H**) Effect of microinjection of AAV5-*V1*, AAV5-*V2*, or AAV5-*Gfp* into the unilateral L3/4 DRGs on levels of p-ERK1/2, ERK1/2, and GFAP in the ipsilateral L3/4 dorsal horn 8 weeks after microinjection. *n =* 6 mice per group. **P < 0.01, by 1-way ANOVA followed by post hoc Tukey’s test.

We then asked whether DRG *NIS*-*lncRNA* upregulation was sufficient for nerve injury–induced nociceptive hypersensitivity. To this end, we microinjected AAV5 expressing the full-length *NIS* V1 (AAV5-*V1*) or V2 (AAV5-*V2*) into unilateral L3/4 DRGs of native male mice. AAV5-*Gfp* was used as a control. As expected, levels of *NIS* V1 and V2 were significantly increased in injected DRGs 8 weeks after viral microinjection (Figure 6A). DRG microinjection of either AAV5-*V1* or AAV5-*V2* led to increases in paw withdrawal frequencies in response to mechanical stimuli and decreases in paw withdrawal latencies in response to heat or cold stimulation on the injected side (Figure 6, B–E). These behavioral changes occurred between 2 and 4 weeks after microinjection and lasted for at least 8 weeks (Figure 6, B–E). Neither viral microinjection altered basal paw withdrawal responses on the noninjected side (Supplemental Figure 19, A–C) and locomotor function (Supplemental Table 1). DRG microinjection of either AAV5-*V1* or AAV5-*V2* also produced stimulation-independent spontaneous ongoing pain 8 weeks after microinjection (Figure 6, F and G). Neuronal and astrocyte hyperactivities indicated by robust increases in the levels of p-ERK1/2 and GFAP, respectively, were also detected in the ipsilateral L3/4 dorsal horn 8 weeks after microinjection (Figure 6H). Similar results were seen in female mice with DRG microinjection of AAV5-*V1* or AAV5-*V2* (Supplemental Figure 20). These findings indicate that, in the absence of nerve injury, DRG *NIS-lncRNA* upregulation likely produces neuropathic pain–like symptoms.

### Upregulated NIS-lncRNA is responsible for the nerve injury–induced Ccl2 activation in injured DRGs.

To elucidate the mechanism by which DRG *NIS-lncRNA* upregulation participates in neuropathic pain, we performed high-throughput RNA sequencing to identify the downstream targets regulated by *NIS-lncRNA* in injured DRGs of male mice. The unbiased gene expression database showed about 816 upregulated and 4 downregulated genes in microinjected DRGs 4 weeks after microinjection of a mixture of AAV5-*V1* and AAV5-*V2* (data not shown). Our previous RNA sequencing database revealed that SNL upregulated approximately 5869 genes and downregulated about 5294 genes in injured DRGs (22). We found about 484 upregulated genes overlapped between 2 databases (Supplemental Figure 21A). They are notably enriched for the immune response/process (Supplemental Figure 21B).

**Figure 7:**
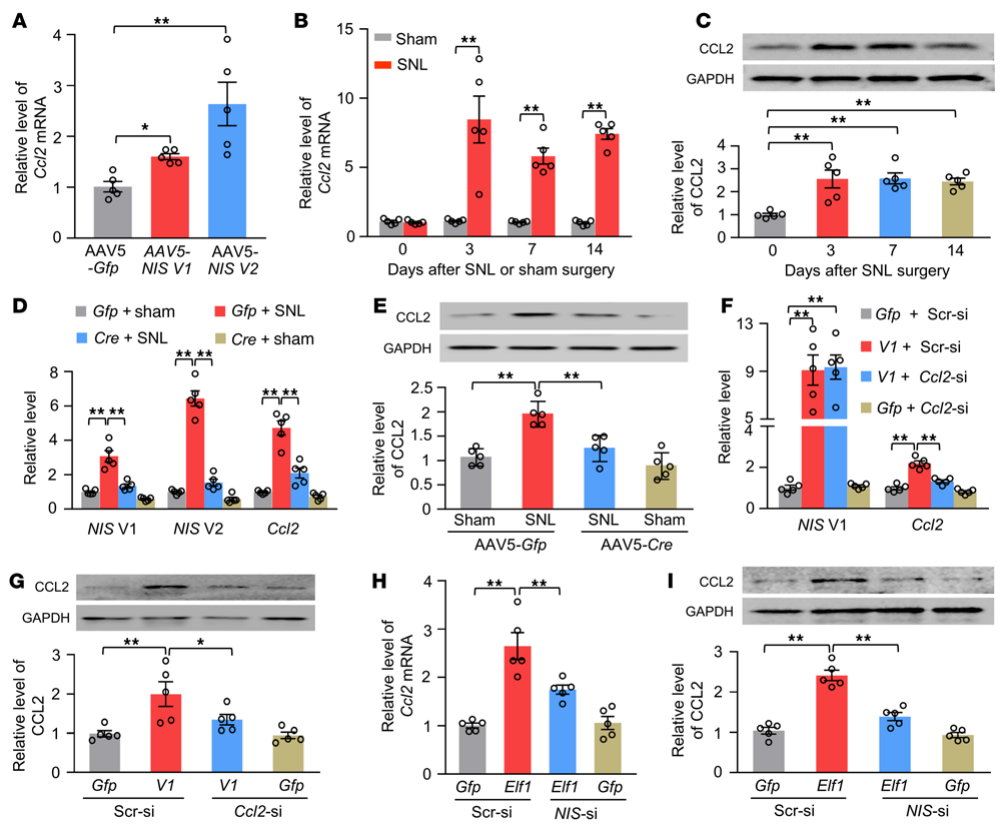
Upregulated *NIS lncRNA* is required for the SNL-induced CCL2 increase in injured DRGs of male mice. Data: mean ± SEM. (**A**) Level of *Ccl2* mRNA in the cultured DRG neurons 3 days after transduction with AAV5-*Gfp*, AAV5-*NIS* V1, or AAV5-*NIS* V2. *n =* 5 repeats per group. *P < 0.05, **P < 0.01, by 2-tailed, unpaired Student’s *t* test. (**B** and **C**) Levels of *Ccl2* mRNA and CCL2 protein in the ipsilateral L4 DRG after surgery. *n =* 20 mice per day per group. **P < 0.01, by 2-way (**B**) or 1-way (**C**) ANOVA with post hoc Tukey’s test. (**D** and **E**) Levels of *NIS* V1, *NIS* V2, and *Ccl2* mRNA and CCL2 protein in the ipsilateral L4 DRG 14 days after surgery in the *NIS*-*lncRNA^fl/fl^* mice with microinjection of AAV5-*Gfp* or AAV5-*Cre* 35 days before surgery. *n =* 20 mice per group. **P < 0.01, by 2-way ANOVA with post hoc Tukey’s test. (**F** and **G**) Levels of *NIS* V1, *Ccl2* mRNA, and CCL2 protein in the ipsilateral L3/4 DRGs 5 days after DRG microinjection of *Ccl2* siRNA (*Ccl2*-si) or scrambled siRNA (Scr-si) in mice with microinjection of AAV5-*Gfp* (*Gfp*) or AAV5-*NIS* V1 (*V1*) 35 days before siRNA microinjection. *n =* 10 mice per group. *P < 0.05, **P < 0.01, by 1-way ANOVA with post hoc Tukey’s test. (**H** and **I**) Levels of *Ccl2* mRNA and CCL2 protein in the ipsilateral L3/4 DRGs 7 days after DRG microinjection of *NIS lncRNA* siRNA (*NIS*-si) or scrambled siRNA (Scr-si) in mice with microinjection of AAV5-*Gfp* or AAV5-*Elf1* (*Elf1*) 35 days before siRNA microinjection. *n =* 10 mice per group. **P < 0.01, by 2-way ANOVA with post hoc Tukey’s test.

**Figure 8:**
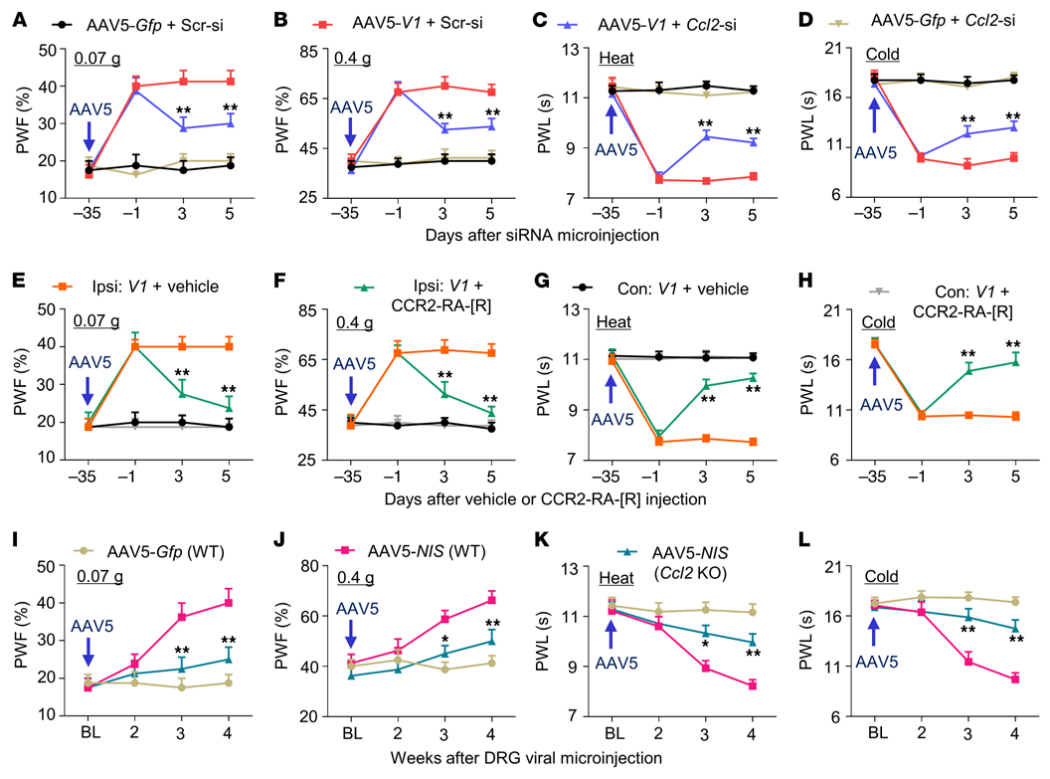
Pharmacological inhibition or genetic knockdown/knockout of DRG CCL2 mitigates nociceptive hypersensitivity caused by DRG *NIS* V1 overexpression in male mice. PWF, paw withdrawal frequency; PWL, paw withdrawal latency. Data: mean ± SEM. (**A**–**D**) Effect of microinjection of *Ccl2* siRNA (*Ccl2*-si) or scrambled siRNA (Scr-si) into ipsilateral L3/4 DRGs on ipsilateral paw withdrawal responses on the days indicated in mice with microinjection of AAV5-*V1* or AAV5-*Gfp* 35 days before siRNA microinjection. *n =* 8 mice per group. **P < 0.01 vs. AAV5-*V1* plus Scr-si group at the corresponding time points by 2-way ANOVA with repeated measures followed by post hoc Tukey’s test. (**E**–**H**) Effect of i.p. administration of the CCR2 receptor antagonist (CCR2-RA-[R]; 5 mg/kg) or vehicle (DMSO) once daily for 5 days on paw withdrawal responses on the ipsilateral (Ipsi) and contralateral (Con) sides on the days indicated in mice with microinjection of AAV5-*V1* (*V1*) 35 days before i.p. injection. *n =* 8 mice per group. **P < 0.01 vs. AAV5-*V1* plus vehicle group at the corresponding time points by 3-way ANOVA with repeated measures followed by post hoc Tukey’s test. (**I**–**L**) Paw withdrawal responses on the weeks indicated after microinjection of AAV5-*Gfp* (*Gfp*) or a mixture of AAV5*-Gfp* and AAV5*-V1* (*NIS*) into unilateral L3/4 DRGs of WT or *Ccl2*-KO mice on the ipsilateral side. *n =* 8 mice per group. *P < 0.05, **P < 0.01 vs. *Gfp*-microinjected WT mice at the corresponding time points, by 3-way ANOVA with repeated measures followed by post hoc Tukey’s test.

The chemokine *Ccl2* mRNA, one most striking gene among these overlapped genes, is a key player in neuropathic pain (11, 13). Overexpression of *NIS* V1 and V2 through transduction of AAV5-*V1* and AAV5-*V2*, respectively, into the cultured DRG neurons significantly increased the level of *Ccl2* mRNA (Figure 7A). Consistent with previous reports (11, 13), SNL increased the amounts of *Ccl2* mRNA and CCL2 protein in the ipsilateral L4 DRG of male mice from 3 to 14 days after SNL (Figure 7, B and C). SNL- or CCI-induced increases of *Ccl2* mRNA and CCL2 protein were markedly blocked in the ipsilateral L4 DRG of male or female *NIS-lncRNA^fl/fl^* mice with DRG microinjection of AAV5*-Cre*, but not AAV5-*Gfp* (Figure 7, D and E, and Supplemental Figure 22A). Conversely, DRG overexpression of *NIS* V1 through microinjection of AAV5-*V1* into unilateral L3/4 DRGs of male mice treated with control scrambled siRNA not only produced the enhanced paw withdrawal responses to mechanical, heat, and cold stimuli on the ipsilateral (not contralateral) side (Figure 8, A–D, and Supplemental Figure 23, A–C), but also elevated the levels of *Ccl2* mRNA and CCL2 protein in the ipsilateral L3/4 DRGs (Figure 7, F and G). Similar changes were detected in female mice with DRG microinjection of AAV5*-V1* or AAV5*-V2* (Supplemental Figure 20 and Supplemental Figure 22B). DRG microinjection of *Ccl2* siRNA or intraperitoneal administration of the CCR2 antagonist CCR2-RA-[R] given on day 35 after AAV5-*V1* microinjection blocked these augmented behavioral responses (Figure 7, F and G, and Figure 8, A–H). Furthermore, DRG overexpression of ELF1 through DRG microinjection of AAV5-*Elf1* also increased the amounts of *Ccl2* mRNA and CCL2 protein in injected DRGs of the male mice treated with control scrambled siRNA (Figure 7, H and I). These increases were significantly attenuated through DRG microinjection of *NIS-lncRNA* siRNA 35 days after AAV5-*Elf1* microinjection (Figure 7, H and I). Taken together, our evidence indicates that upregulated *NIS-lncRNA* likely participates in nerve injury–induced transcriptional activation of the *Ccl2* gene in injured DRGs.

### Mimicking nerve injury–induced DRG NIS-lncRNA upregulation increases CCL2-mediated DRG neuronal excitability.

**Figure 9:**
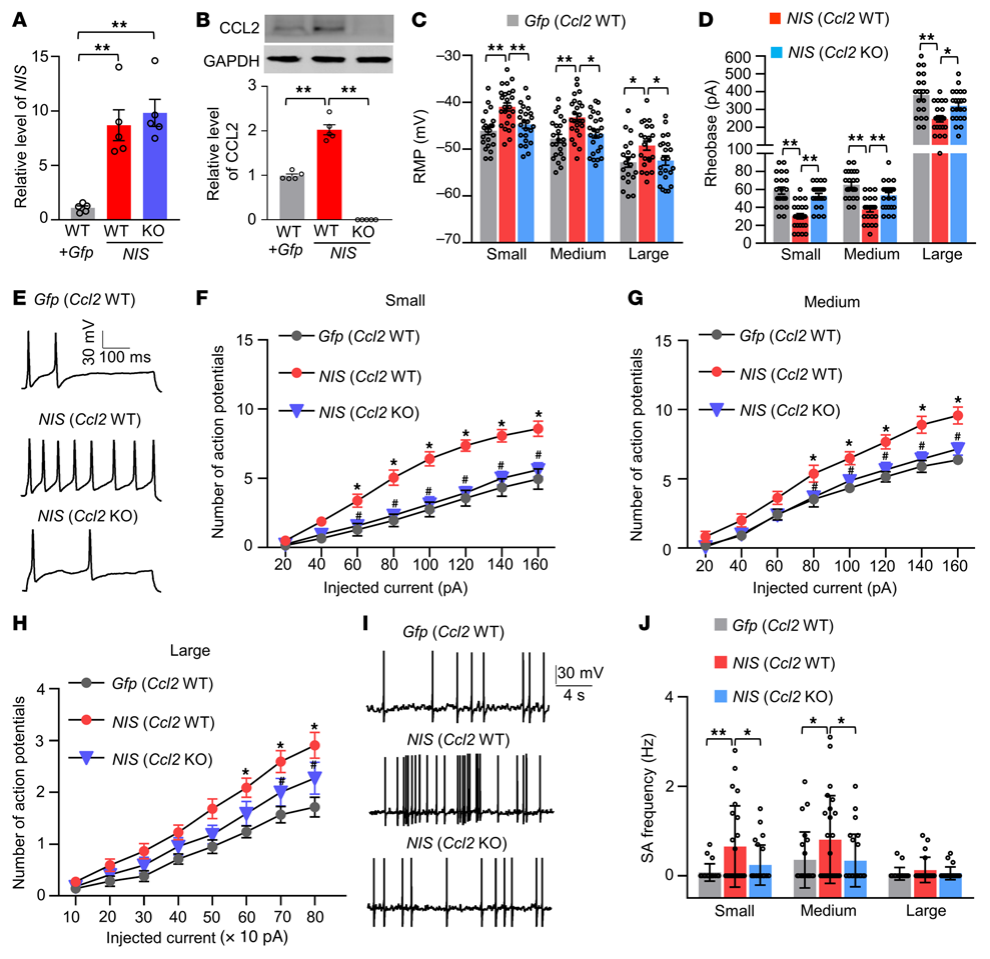
DRG overexpression of *NIS-lncRNA* increases CCL2-mediated DRG neuronal excitability. Data: mean ± SEM. (**A** and **B**) Levels of *NIS* V1 and CCL2 protein in the ipsilateral L3/4 DRGs 4 weeks after microinjection of AAV5-*Gfp* alone (*Gfp*) or a mixture of AAV5*-Gfp* and AAV5-*V1* (*NIS*) into unilateral L3/4 DRGs of WT or *Ccl2*-KO mice. *n =* 10 mice per group. **P < 0.01, by 2-way ANOVA with post hoc Tukey’s test. (**C** and **D**) Resting membrane potentials (RMP) and rheobases. *n =* 22 small, 23 medium, and 20 large cells from 14 *Gfp-*microinjected WT mice; *n =* 24 small, 24 medium, and 22 large cells from 18 *NIS-*microinjected WT mice; *n =* 24 small, 24 medium, and 22 large cells from 18 *NIS-*microinjected *Ccl2*-KO mice. *P < 0.05, **P < 0.01, by 2-way ANOVA with post hoc Tukey’s test. (**E**–**H**) Numbers of evoked action potentials after application of different currents as indicated. (**E**) Representative traces of evoked action potentials (100 pA, 500 ms) in small DRG neurons. Numbers of recorded cells and mice used are the same as in **C** and **D**. *P < 0.05 vs. *Gfp-*microinjected WT mice at the corresponding injected intensity and ^#^*P* < 0.05 vs. *NIS-*microinjected WT mice at the corresponding injected intensity, by 2-way ANOVA with post hoc Tukey’s test. (**I** and **J**) Frequencies of spontaneous activity (SA). (**I**) Representative SA traces of small DRG neurons. Numbers of recorded cells and mice used are the same as in **C** and **D**. *P < 0.05, **P < 0.01, by 2-way ANOVA with post hoc Tukey’s test.

Given that CCL2 excites DRG neurons (16), we further examined whether mimicking nerve injury–induced DRG *NIS-lncRNA* upregulation altered CCL2-mediated DRG neuronal excitability. Whole-cell current-clamp recording was carried out in acutely disassociated neurons from the ipsilateral L3/4 DRGs 4–5 weeks after microinjection of AAV5-*Gfp* (*Gfp*) alone or a mixed viral solution of AAV5–*NIS-lncRNA* V1 and AAV5-*Gfp* (*NIS*) into the unilateral L3/4 DRGs of wild-type (WT) or *Ccl2-*knockout (KO) male mice (Figure 9, A and B). Only green DRG neurons were recorded (Supplemental Figure 24A). As expected, the enhancements in response to mechanical, heat, and cold stimuli on the ipsilateral (but not contralateral) side from the *NIS*-microinjected WT mice were impaired in the *NIS-*microinjected *Ccl2-*KO mice (Figure 8, I–L, and Supplemental Figure 23, D–F). Compared with the *Gfp*-microinjected WT mice, small, medium, and large DRG neurons from the *NIS*-microinjected WT mice showed depolarization by 5.1, 4.5, and 3.6 mV, respectively, in resting membrane potentials (Figure 9C) and reductions by 49%, 43%, and 36%, respectively, in the rheobase (Figure 9D and Supplemental Figure 24B). Moreover, small, medium, and large DRG neurons from the *NIS*-microinjected WT mice also exhibited increases in the number of action potentials evoked by injected currents compared with those in the *Gfp*-microinjected WT mice (Figure 9, E–H). However, these changes were not observed in small, medium, and large DRG neurons from the *NIS-*microinjected *Ccl2-*KO mice (Figure 9, C–H). No marked changes in membrane input resistance and other action potential parameters, such as threshold, amplitude, overshoot, or afterhyperpolarization amplitude, were seen among 3 microinjected groups (Supplemental Table 2). In addition, a significant increase in the frequency of spontaneous activity was observed in small and medium, but not large, DRG neurons from the *NIS*-microinjected WT mice (Figure 9, I and J). These increases were also markedly attenuated in the *NIS*-microinjected *Ccl2-*KO mice (Figure 9, I and J). Together, these findings indicate that CCL2 may mediate the *NIS-lncRNA* upregulation–induced increase in DRG neuronal excitability.

### FUS mediates NIS-lncRNA upregulation–induced Ccl2 expression in injured DRGs after nerve injury.

**Figure 10:**
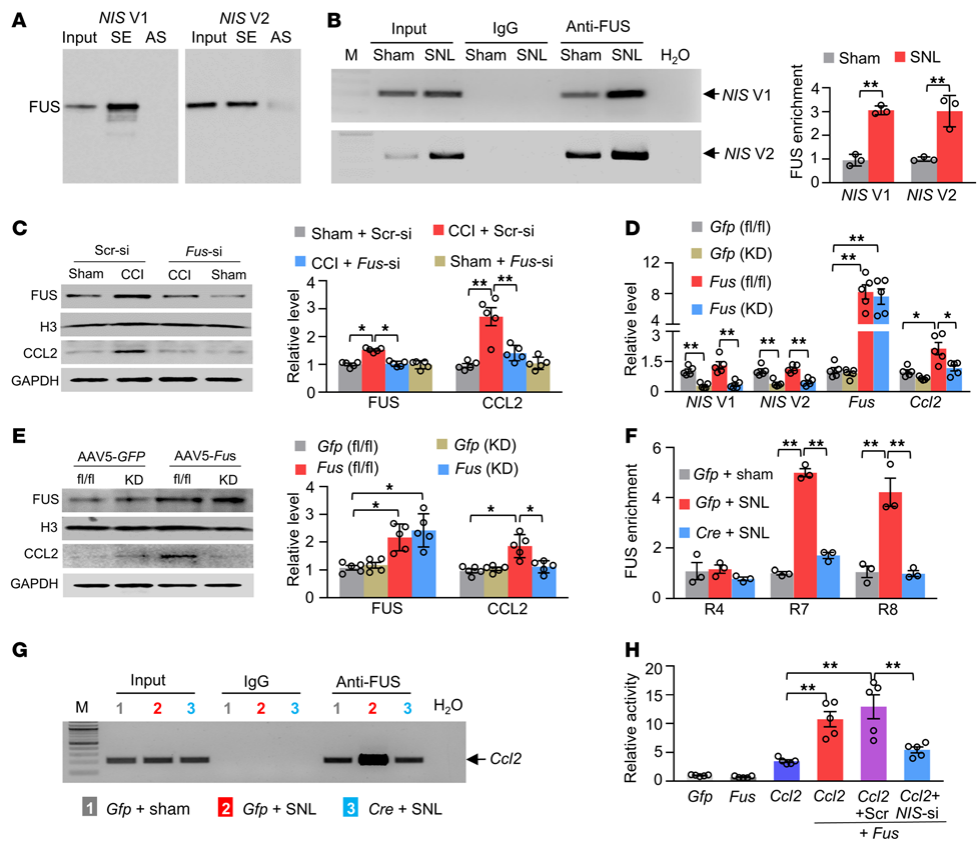
*NIS-lncRNA* is required for FUS-triggered CCL2 expression in injured DRGs. Data: mean ± SEM. (**A**) FUS pulled down by the *NIS* V1 and V2 sense (SE), but not their antisense (AS), RNA probes in cultured DRG neurons. *n =* 3 repeats. (**B**) *NIS* V1 and V2 fragments immunoprecipitated by mouse anti-FUS antibody in the ipsilateral L3/4 DRGs 7 days after surgery. *n =* 45 mice per group. **P < 0.01, by 2-tailed, unpaired Student’s *t* test. (**C**) Levels of FUS and CCL2 proteins in the ipsilateral L3/4 DRGs 5 days after surgery in mice with DRG microinjection of *Fus* siRNA (*Fus*-si) or scrambled siRNA (Scr-si) 3 days before surgery. *n =* 10 mice per group. *P < 0.05, **P < 0.01, by 2-way ANOVA with post hoc Tukey’s test. (**D** and **E**) Levels of *NIS* V1, *NIS* V2, *Fus* mRNA, *Ccl2* mRNA, and their proteins in the ipsilateral L3/4 DRGs 28 days after DRG microinjection of AAV5-*Gfp* or AAV5-*Fus* in *NIS-lncRNA^fl/fl^* mice (fl/fl) or *NIS-lncRNA–*knockdown mice (KD). *n =* 10 mice per group. *P < 0.05, **P < 0.01, by 2-way ANOVA with post hoc Tukey’s test. (**F** and **G**) Three regions (R4, R7, and R8) within the *Ccl2* gene promoter were immunoprecipitated by mouse anti-FUS in the ipsilateral L3/4 DRGs 7 days after surgery from the *NIS-lncRNA^fl/fl^* mice with microinjection of AAV5-*Gfp* (*Gfp*) or AAV5-*Cre* (*Cre*) 28 days before surgery. Representative image shows the binding of FUS to R7. *n =* 36 mice per group. **P < 0.01, by 2-way ANOVA with post hoc Tukey’s test. (**H**) *Ccl2* gene promoter activities in in vitro CAD cells transfected as shown. *Gfp*: vector expressing *Gfp* mRNA; *Fus*: vector expressing *Fus* mRNA; *Ccl2*: pGL3 reporter vector with *Ccl2* promoter; Scr: scrambled siRNA; *NIS*-si: *NIS-lncRNA* siRNA. *n =* 5 repeats per treatment. **P < 0.01, by 1-way ANOVA with post hoc Tukey’s test.

Finally, we addressed how the *Ccl2* gene was transcriptionally activated by *NIS-lncRNA* in injured DRGs under neuropathic pain conditions. RNA-binding proteins participate in RNA transcription (25). To search for *NIS-lncRNA–*binding proteins, we designed specific sense RNA probes for *NIS* V1 and V2, respectively, and identified *NIS-lncRNA*–binding proteins using the comprehensive identification of RNA-binding proteins by mass spectrometry (ChIRP-MS) assay. We found that *NIS* V1 and V2 interacted with 409 and 600 proteins, respectively, of which 130 proteins were overlapped, in the cultured DRG neurons (Supplemental Figure 25A). These overlapped proteins are notably enriched for RNA binding (Supplemental Figure 25B). Fused in sarcoma (FUS), an RNA-binding protein (26), appeared to be a most likely binding partner of *NIS-lncRNA* among these overlapped proteins. Indeed, FUS could be pulled down, respectively, by specific *NIS* V1 and V2 sense RNA probes, but not by the corresponding negative control antisense RNA probes, in in vitro–cultured DRG neurons (Figure 10A). Both *NIS* V1 and V2 fragments were immunoprecipitated by anti-FUS antibody (but not normal purified IgG) in in vivo sham DRGs of male mice (Figure 10B). These 2 immunoprecipitated activities were dramatically increased 3.2-fold and 3-fold, respectively, in injured DRGs on day 7 after SNL compared with the corresponding sham groups (Figure 10B). Our findings indicate an increase in the ability of *NIS-lncRNA* to bind to FUS in injured DRGs following peripheral nerve injury.

We further found that FUS overexpression through transduction of AAV5 expressing full-length *Fus* mRNA (AAV5-*Fus*) plus AAV5 expressing control scrambled shRNA (AAV5-*shScr*) into cultured DRG neurons considerably elevated the level of CCL2 (Supplemental Figure 26). However, this elevation did not occur in cultured DRG neurons transduced with AAV5-*Fus* plus AAV5 expressing *Fus* shRNA (AAV5-*shFus*; Supplemental Figure 26), suggesting that this CCL2 elevation is in specific response to FUS overexpression. Moreover, preventing the CCI-induced increase in the level of FUS through DRG *Fus* siRNA microinjection blocked an increase in the amount of CCL2 in injured DRGs on day 5 after CCI and impaired mechanical, heat, and cold nociceptive hypersensitivities on days 3 and 5 after CCI on the ipsilateral side (Figure 10C and Supplemental Figure 27, A–G). DRG overexpression of FUS through DRG microinjection of AAV5-*Fus* (but not AAV5-*Gfp*) significantly elevated the levels of *Ccl2* mRNA and CCL2 protein in microinjected DRGs of the *NIS-lncRNA^fl/fl^* male mice (Figure 10, D and E). However, these elevations were not seen in the *NIS-*KD mice (Figure 10, D and E). The ChIP assay revealed that FUS bound to 3 regions (R4: –196/+5; R7: –589/–399; and R8: –748/–542) of the *Ccl2* gene promoter, as evidenced by the amplification of only these 3 regions (out of 9 regions from –863 to +546) from the complexes immunoprecipitated with anti-FUS antibody in nuclear fraction from sham DRGs (Figure 10, F and G, and Supplemental Figure 28). The binding activities in R7 and R8, but not R4, from injured DRGs of AAV5-*Gfp*–microinjected *NIS-lncRNA^fl/fl^* mice on day 7 after SNL increased 5.4-fold and 3-fold, respectively, compared with those after sham surgery (Figure 10, F and G). These increases were absent in the AAV5-*Cre*–microinjected SNL *NIS-lncRNA^fl/fl^* mice (Figure 10, F and G). The luciferase assay showed that the *Ccl2* gene promoter was significantly activated in in vitro CAD cells cotransfected with full-length *Fus* vector plus control scrambled siRNA, but not with full-length *Fus* vector plus *NIS-lncRNA* siRNA (Figure 10H). Given that *NIS-lncRNA* also bound to R7 of the *Ccl2* gene promoter (Supplemental Figure 29), the evidence indicates that *NIS-lncRNA* may function as a molecular scaffolder to recruit FUS to the *Ccl2* gene promoter to trigger its transcription in injured DRGs after peripheral nerve injury.

Consistently, marked increases in the activities in R7 and R8 of the *Ccl2* gene promoter were seen in in vitro CAD cells cotransfected with control scrambled shRNA plus full-length *NIS* V1 or V2 vector (Supplemental Figure 30). However, these increases were significantly reduced in CAD cells cotransfected with *Fus* shRNA plus full-length *NIS* V1 or V2 vector (Supplemental Figure 30). Furthermore, a significant increase in the amount of *Ccl2* mRNA was detected in cultured DRG neurons cotransduced with AAV5-*shScr* plus AAV5-*V1* or AAV5-*V2*, but not with AAV5-*shFus* plus AAV5-*V1* or AAV5-*V2* (Supplemental Figure 31, A and B). Additionally, DRG microinjection of *Fus* siRNA (but not control scrambled siRNA) blocked the AAV5-*V1*–induced increase in the level of *Ccl2* mRNA and CCL2 protein in injected DRGs on day 5 after microinjection (Supplemental Figure 31, C and D) and blunted the AAV5-*V1*–induced mechanical, heat, and cold nociceptive hypersensitivities on days 3 and 5 after microinjection in male mice (Supplemental Figure 32, A–G). Given that *NIS* V1 and *NIS* V2 were coexpressed with *Fus* mRNA and *Ccl2* mRNA in individual large, medium, and small DRG neurons (Supplemental Figure 12), our data indicate that FUS mediates the *NIS-lncRNA*–promoted increase of *Ccl2* expression in injured DRGs after peripheral nerve injury.

## Discussion

Although peripheral neuropathic pain has been intensively investigated for several decades, how peripheral nerve injury leads to nociceptive hypersensitivity is still elusive. In the present study, we identified a nerve injury–specific lncRNA and reported its upregulation triggered by an elevation of the transcription factor ELF1 in injured DRGs after peripheral nerve injury. This upregulation contributes to the development and maintenance of nerve injury–induced nociceptive hypersensitivity through promoting FUS-triggered *Ccl2* gene expression in injured DRG neurons. Given that DRG CCL2 is required for neuropathic pain genesis (14, 17–19), *NIS-lncRNA* likely is an endogenous initiator of neuropathic pain.

Like other lncRNAs (24, 27), *NIS-lncRNA* could be transcriptionally activated in the DRG neurons after peripheral nerve injury. Under normal conditions, the level of either of the 2 variants of *NIS-lncRNA* was extremely low or undetectable in both mouse and human DRGs. However, their expression was dramatically increased in injured DRGs, but not intact DRGs and ipsilateral spinal cord, in preclinical mouse models of neuropathic pain caused by SNL or CCI. Both models are complementary, as SNL-induced pain results mainly from direct nerve injury (28), whereas CCI-induced pain is initiated primarily by ischemia (29). The increased levels of *NIS* V1 and V2 occurred predominantly in large and medium DRG neurons, although both variants were expressed in all types of DRG neurons. *NIS* V1 and V2 were undetected in satellite cells and macrophages in injured DRGs. Moreover, quantitative analysis of nuclear/cytoplasmic RNA, subcellular fractionation of DRGs, and RNAscope ISH assay showed that both variants were more enriched in the neuronal nucleus than in the cytoplasm. Interestingly, peripheral tissue injury and inflammation did not substantially change basal expression of DRG *NIS-lncRNA*. How transcriptional activation of DRG *NIS-lncRNA* occurs only in a specific response to peripheral nerve injury is still unknown, but our findings strongly suggest that this activation is related to a nerve injury–induced increase in DRG transcription factor EFL1. Whether *Elf1* mRNA, like *NIS-lncRNA*, in injured DRGs responds specifically to nerve injury remains to be determined. Admittedly, other transcription factors that may also be involved in *NIS-lncRNA* upregulation in injured DRGs cannot be excluded. In addition, epigenetic modifications and/or an increase in RNA stability may participate in *NIS-lncRNA* upregulation under neuropathic pain conditions. These possibilities will be investigated in future studies.

FUS participates in the *NIS-lncRNA* upregulation–induced increase in CCL2 expression in injured DRGs. By interacting with proteins, RNA, and DNA, lncRNAs modulate gene expression (21). We showed that nerve injury increased the binding of *NIS* V1/V2 to FUS in injured DRGs. We also demonstrated that FUS interacted with the *Ccl2* gene promoter, triggered *Ccl2* transcriptional activation, and participated in nerve injury–induced increase of DRG CCL2 expression. This transcriptional activation may require the transcription factor EBF1, as interaction of FUS with EBF1 enhanced chromatin opening (30). *NIS-lncRNA* knockdown not only reduced the FUS-triggered *Ccl2* promoter activation and DRG *Ccl2* mRNA/CCL2 protein expression but also blocked the nerve injury–induced increase in binding of FUS to the *Ccl2* promoter in injured DRGs. Given that *NIS-lncRNA* also interacted with the *Ccl2* promoter in the DRG, our findings suggest that *NIS-lncRNA* functions as a molecular scaffolder to recruit FUS to the *Ccl2* promoter. Nerve injury–induced upregulation of *NIS* V1 and V2 may result in their increased binding to FUS, recruiting increased FUS to the *Ccl2* promoter to transcriptionally activate *Ccl2* expression in injured DRGs. This conclusion is further supported by the facts that *NIS* V1/V2–induced activation of the *Ccl2* promoter, increase of DRG *Ccl2* mRNA/CCL2 protein expression, and nociceptive hypersensitivities on the ipsilateral side were attenuated by *Fus* knockdown and that *NIS* V1/V2 coexisted with *Fus* and *Ccl2* mRNAs in individual DRG neurons. Thus, FUS appears to mediate the functional role of *NIS-lncRNA* in nerve injury–induced CCL2 expression in injured DRGs. Our ChIRP-MS assay showed that, in addition to FUS, other RNA-binding proteins also bind to *NIS-lncRNA* in DRGs. Potential contribution of these proteins to *NIS-lncRNA* promotion of CCL2 expression in injured DRGs cannot be ruled out.

Upregulated DRG *NIS-lncRNA* participates in neuropathic pain through promoting *Ccl2* gene expression in injured DRGs. We revealed that blocking nerve injury–induced upregulation of *NIS-lncRNA* through DRG microinjection of *NIS-lncRNA* siRNA in CD1 mice blocked the development of nerve injury–induced hypersensitivity. Likewise, genetic knockdown of DRG *NIS-lncRNA* in *NIS*-KD mice or AAV5-*Cre*–microinjected *NIS-lncRNA^fl/fl^* mice attenuated the increases of *Ccl2* mRNA and CCL2 protein in injured DRGs and impaired development and maintenance of nerve injury–induced nociceptive hypersensitivities in both male and female mice. Mimicking nerve injury–induced DRG *NIS-lncRNA* upregulation through DRG microinjection of AAV5*-V1* or AAV5-*V2* in CD1 male and female mice increased the expression of DRG *Ccl2* mRNA and CCL2 protein, elevated the CCL2-mediated DRG neuronal excitability, and enhanced basal response to noxious stimuli. Moreover, these enhanced nociceptive responses were significantly mitigated after pharmacological inhibition or genetic DRG knockout/knockdown of CCL2. These findings indicate that CCL2 mediates the functional role of *NIS-lncRNA* in neuropathic pain in injured DRGs. Interestingly, DRG *NIS-lncRNA* expression was not altered after peripheral inflammation, whereas *Ccl2* mRNA and CCL2 protein were upregulated in innervating DRGs following induction of osteoarthritis (31). This suggests that the *Ccl2* gene could be regulated through *NIS-lncRNA*–independent mechanisms under inflammatory conditions. It should be noted that, besides CCL2, blocking DRG *NIS-lncRNA* upregulation may affect nerve injury–induced changes in other DRG transcripts. We revealed that at least 484 genes (including *Ccl2*) were upregulated in microinjected DRGs after DRG *NIS-lncRNA* overexpression. Thus, other potential mechanisms by which *NIS-lncRNA* contributes to neuropathic pain cannot be excluded.

Molecular mechanisms underlying neuropathic pain are still incompletely understood. Knockdown or knockout of Nav1.7, Nav1.8, or Nav1.9 in small DRG neurons reduced inflammatory pain, but did not alter neuropathic pain (32–34). The use of diphtheria toxin to selectively ablate most nociceptors (80%) in mouse DRGs did not affect nerve injury–induced mechanical or thermal pain hypersensitivities (35). Interestingly, a nerve injury–induced increase in spontaneous ectopic activity was detected in injured myelinated afferents (but not unmyelinated fibers) and the corresponding medium and large DRG neurons (36, 37). Aβ fibers mediated nerve injury–induced mechanical allodynia (38, 39). The cooling applied on mouse and human peripheral axons induced bursts of action potentials in myelinated A fibers, but not unmyelinated C fibers, after oxaliplatin administration (40). Consistent with this, local knockdown of Nav1.6 expressed mainly in medium and large DRG neurons markedly attenuated CCI- or SNL-induced mechanical hypersensitivity and blocked abnormal spontaneous activity in myelinated neurons of injured DRGs (41). The evidence suggests that the nerve injury–induced increase in excitability of medium and large DRG neurons drives the release of neurotransmitters/neuromodulators from their central terminals and leads to spinal sensitization and enhanced response to peripheral stimuli.

Like *NIS-lncRNA*, *Ccl2* mRNA and CCL2 protein were significantly increased predominantly in large and medium neurons of injured DRG after peripheral nerve injury (11). CCL2 is considered an endogenous initiator of neuropathic pain (17–20) by directly sensitizing nociceptors (15), exciting DRG neurons (15–17), and enhancing glutamate release of primary afferents and postsynaptic glutamate receptor function in the superficial dorsal horn (18, 42). We conclude that the anti-nociceptive effect produced by blocking upregulated DRG *NIS-lncRNA* in neuropathic pain likely results from inactivation of the *Ccl2* gene, silencing of CCL2 protein expression, and reduction of neuronal excitability, particularly in medium and large neurons of injured DRGs. The latter may cause a decrease in the release of primary afferent transmitters/neuromodulators and consequent impairment of spinal cord central sensitization formation. In support of this conclusion, we demonstrated that blocking *NIS-lncRNA* upregulation in injured DRGs diminished nerve injury–induced hyperactivation in dorsal horn neurons and astrocytes.

In conclusion, we demonstrated a *NIS-lncRNA*–triggered mechanism by which *Ccl2* is transcriptionally activated in injured DRGs selectively under neuropathic pain conditions. Given that *NIS-lncRNA* was upregulated in specific response to peripheral nerve injury only in injured DRG neurons and that preventing this upregulation blocked the development and reversed the maintenance of neuropathic pain without affecting basal/acute pain and locomotor function, *NIS-lncRNA* may be a potential target for treatment to alleviate neuropathic pain.

## Methods

A detailed description of the materials and methods used is provided in Supplemental Methods.

The RNA sequencing data sets presented in this study have been deposited in the NCBI Gene Expression Omnibus database (GEO GSE206043).

### Study approval

The Animal Care and Use Committee at Rutgers New Jersey Medical School approved all experimental procedures with animals, which were also consistent with the ethical guidelines of the US National Institutes of Health and the International Association for the Study of Pain. Human DRGs were recovered, in collaboration with LifeCenter, Cincinnati, from deidentified organ donors who gave written informed consent; the procedure was approved by the University of Cincinnati Internal Review Board (IRB#00003152; Study ID 2015-5302).

## Author contributions

YXT conceived the project and supervised all experiments. S Du, SW, XF, BW, and YXT designed the project. S Du, SW, XF, BW, SX, LL, LZ, GG, QM, XG, TB, and HZ carried out molecular, biochemical, morphological, and behavioral experiments. S Du carried out patch clamp recording. A Bunk, FK, and S Davidson collected and cultured human DRGs. TL and HL performed mass spectrometry and data analysis. S Du, SW, XF, BW, SX, LL, JS, A Bekker, and YXT analyzed the remaining data. YXT wrote/edited the manuscript. All authors read and discussed the manuscript.

## Supplementary Material

Supplemental data

## Version 1 07/01/2022

Electronic publication
