# Supplementary material for: A nerve injury–specific long noncoding RNA promotes neuropathic pain by increasing Ccl2 expression
Source: J Clin Invest. 2022 Jul 1;132(13):e153563. doi: 10.1172/JCI153563 (PMC9246381; doi:10.1172/JCI153563)
Supplement: Supplemental data [file jci-132-153563-s104.pdf]

Supplemental Methods

Animals.

Male and female mice weighing 25–30 g were used for the experiments. CD1 mice (purchased from Charles River Laboratory, Wilmington, MA), *NIS-lncRNA*$^{fl/fl}$ mice (generated by Biocytogen, Worcester, MA), *Advillin*$^{Cre/+}$ mice (provided by Dr. Fan Wang of Duke University) and *Ccl2* knockout (Ccl2 $^{-/-}$) mice (purchased from The Jackson Laboratory, Stock No: 004434) were used in this study. *NIS-lncRNA*$^{fl/fl}$ mice were bred onto a C57B/L6 background for at least 3 generations by crossing the heterozygotes in our facility. Male sensory neuron-specific *Cre* line *Advillin*$^{Cre/+}$ mice were crossed with female *NIS-lncRNA*$^{fl/fl}$ mice to obtain conditional *NIS-lncRNA* knockdown (*NIS* KD) mice. All mice were kept in the central housing facility at Rutgers New Jersey Medical School under a standard 12-h light/dark cycle, with water and food pellets available ad libitum. The Animal Care and Use Committee at Rutgers New Jersey Medical School approved all experimental procedures, which were also consistent with the ethical guidelines of the US National Institutes of Health and the International Association for the Study of Pain. The animals were randomly assigned into different groups. The experimenters were blinded to all treated conditions. All efforts were made to minimize mouse suffering and to reduce the number of mice used. No animals used were excluded from the experiments.

Human DRG donors

DRGs were recovered from deidentified, consented organ donors in collaboration with LifeCenter, Cincinnati. The procedure was approved by University of Cincinnati Internal Review Board (IRB#00003152; Study ID: 2015-5302). Three donors included in this study were male 47-58 years old. Lumbar level 4-5 DRGs were collected from both sides within 1.5 hours of

aortic cross clamp and transported to an onsite laboratory in an NMDG-based solution for tissue processing and primary cell dissociation and culture following protocols described in detail previously (43).

Animal models

For trauma-induced neuropathic pain models, sciatic nerve chronic constriction injury (CCI) and fourth lumbar (L4) spinal nerve ligation (SNL) in mice were carried out as described previously(23, 27, 44-46). Briefly, animals were placed under anesthesia with isoflurane. For the SNL model, unilateral L4 spinal nerve was ligated with 7-0 silk suture and then transected at the distal site. For the CCI model, unilateral sciatic nerve was exposed and loosely ligated with 7-0 silk thread at four sites at intervals of about 1 mm, proximal to the trifurcation of the sciatic nerve. Sham animals received an identical surgery but without the ligation or transection of the respective nerve. The sodium mono-iodoacetate (MIA, MilliporeSigma, Burlington, MA)-induced chronic inflammatory pain model was carried out as previously described (47). Briefly, under isoflurane anesthesia, mice were injected intra-articularly with 5 µl of MIA at a concentration of 50 µg/µl (diluted in 0.9% saline), with the needle passing behind the patellar ligament into the joint space of the unilateral knee. The complete Freund's adjuvant (CFA)-induced chronic inflammatory pain model was established as previous published(45;48;49). The 20 µl of undiluted CFA (Sigma-Aldrich) was injected subcutaneously into the plantar surface of one hind paw. For the incisional pain model as described previously(48;50;51), a 0.8-cm longitudinal incision was made through skin, fascia and muscle of unilateral plantar aspect of the hind paw in isoflurane -anesthetized mice.

Behavioral analysis

The evoked behavioral testing, including mechanical, heat, and cold tests, was carried out in sequential order at 1-hour intervals. Conditional place preference (CPP) testing was performed 2 or 4 weeks after surgery. Locomotor function testing was carried out before the tissue collection. All mice were habituated 1 to 2 hours every day for 2-3 days before basal behavioral testing.

Paw withdrawal thresholds in response to mechanical stimulation were measured with two calibrated von Frey filaments (0.07 and/or 0.4 g, Stoelting Co., Wood Dale, IL) as described(23, 24, 44, 52). Briefly, mice were placed in a Plexiglas chamber on an elevated mesh screen and allowed to habituate for 30 min. Each von Frey filament was applied to the plantar sides of both hind paws 10 times. A quick withdrawal of the paw was regarded as a positive response. The number of positive responses among 10 applications was recorded as percentage withdrawal frequency [(number of paw withdrawals/10 trials) × 100 = % response frequency].

Paw withdrawal latencies in response to noxious heat stimulation were examined as published (23, 24, 44, 52). Briefly, mice were placed in a Plexiglas chamber on a glass plate. A beam of light was emitted from a hole in the light box of a Model 336 Analgesia Meter (IITC Inc. Life Science Instruments. Woodland Hills, CA) and applied to the middle of the plantar surface of each hind paw. A quick lift of the hind paw was regarded as a signal to turn off the light. The length of time between the start and the stop of the light beam was defined as the paw withdrawal latency. For each side, 4-5 trials at 5-min intervals were carried out. A cutoff time of 20 s was used to avoid tissue damage to the hind paw.

Paw withdrawal latencies to noxious cold (0 °C) were observed as described(23, 24, 44, 52). Mice were placed in a Plexiglas chamber on the cold aluminum plate, the temperature of which was monitored continuously by a thermometer. The paw withdrawal latency was recorded as the length of time between placement and the first sign of the mouse jumping and/or flinching. Each

trial was repeated three times at 10-min intervals on the ipsilateral side. To avoid tissue damage, a cut-off time of 20 sec was used.

CPP test was carried out as described with minor modifications (23, 24, 44, 52). Briefly, an apparatus with two Plexiglas chambers connected with an internal door (Med Associates Inc., St. Albans, VT) was used. One of the chambers was made of a rough floor and walls with black and white horizontal stripes, and another one was composed of a smooth floor and walls with black and white vertical stripes. Movement of the mice and time spent in each chamber were monitored by photobeam detectors installed along the chamber walls and automatically recorded in MED-PC IV CPP software. Mice were first preconditioned for 30 min with full access to two chambers to habituate them to the environment. At the end of the preconditioning phase, basal duration spent in each chamber was recorded within 15 min to check whether animals had a preexisting chamber bias. Mice spending more than 80% or less than 20% of total time in any chamber were excluded from further testing. The conditioning protocol was performed for the following 3 days with the internal door closed. The mice first received an intrathecal injection of saline (5 µl) specifically paired with one conditioning chamber in the morning. Six hours later, lidocaine (0.8 % in 5 µl of saline) was given intrathecally paired with the opposite conditioning chamber in the afternoon. Lidocaine at this dosage did not affect motor function. The injection order of saline and lidocaine was switched every day. On the test day, at least 20 hours after the conditioning, the mice were placed in one chamber with free access to both chambers. The duration of time that each mouse spent in each chamber was recorded for 15 min. Score differences were calculated as test time-preconditioning time spent in the lidocaine chamber.

Locomotor function was examined as described(23, 24, 44, 52). Three reflexes were conducted as follows. For the placing reflex, the placed positions of the hind limbs were slightly

lower than those of the forelimbs, and the dorsal surfaces of the hind paws were brought into contact with the edge of a table. Whether the hind paws were placed on the table surface reflexively was recorded. For the grasping reflex, after the mouse was placed on a wire grid, whether the hind paws grasped the wire on contact was recorded. For the righting reflex, when the mice were placed on its back on a flat surface, whether it immediately assumed the normal upright position was recorded. Each trial was repeated 5 times at 5-min interval and the scores for each reflex were recorded based on counts of each normal reflex.

DRG microinjection

DRG microinjection was conducted as described with minor modification (53-55). Briefly, after the mouse was anesthetized with isoflurane, a dorsal midline incision was made in the lower lumbar back region. The unilateral L4 and/or L3 articular processes were exposed and then removed. Viral solution (1 µl/DRG, 4-9 × 10^{12}) or siRNA solution (1 µl/DRG, 40-80 µM) was injected into ipsilateral exposed L4 or L3/4 DRGs with the use of a glass micropipette connected to a Hamilton syringe. After injection, the 10 min pipette retention was used before it was removed. The surgical field was irrigated with sterile saline and the skin incision closed with wound clips. None of the microinjected mice showed signs of paresis or other abnormalities. Injected DRGs that were stained with hematoxylin/eosin confirmed the integrity of their structure and demonstrated no visible leukocytes.

Cell culture and transfection

HEK-293T (purchased from ATCC) or CAD cell (provided by Dr. Virgil Muresan at Rutgers New Jersey Medical School) cultures and DRG neuronal cultures were prepared as previously described(23, 24, 44, 52). Briefly, HEK-293T cells were cultured in Dulbecco’s modified Eagle’s medium/high glucose medium (Gibco/Thermo Fisher Scientific) containing 10% fetal

bovine serum (FBS) and 1% antibiotics. CAD cells were cultured in Dulbecco's modified Eagle's medium/F-12 HEPES (Gibco/Thermo Fisher Scientific) containing 8% fetal bovine serum (FBS) and 1% antibiotics. For primary DRG neuronal cultures, after the mice (≥ 4 weeks) were euthanized with isoflurane, all DRGs were collected in cold Neurobasal Medium (Gibco/ThermoFisher Scientific) containing 10% fetal bovine serum (JR Scientific, Woodland, CA), 5 mL L-glutamine (200 mM) (Gibco/ThermoFisher Scientific), 10 mL B-27® Supplement (50x) (Gibco/ThermoFisher Scientific), 100 units/ml Penicillin and 100 µg/ml Streptomycin (Quality Biological, Gaithersburg, MD). The DRGs were then treated with enzyme solution (5 mg/ml dispase, 1 mg/ml collagenase type I) in Hanks' balanced salt solution (HBSS) without Ca^{2+} and Mg^{2+} (Gibco/ThermoFisher Scientific). After trituration and centrifugation, dissociated cells were resuspended in mixed Neurobasal Medium and plated in a six-well plate coated with 50 µg/ml poly-D-lysine (Sigma, St. Louis, MO) at 1.5-4 × 10^5 cells. The cells were incubated at 5% CO_2 and 37 ℃. On the second day, 4-10 µl of virus (titer ≥ 6-9 × 10^{12}/µl), 300-500 ng of vector or siRNA (100 nM; transfected with Lipofectamine 2000) was added to each 2 ml well. Cells were collected 3 days later.

Quantitative real-time reverse transcription (RT)-PCR

The unilateral L3/4 DRGs from two adult CCI, corresponding sham surgery, incisional, CFA, or MIA mice, the unilateral L4 DRGs from four adult SNL or corresponding sham mice, or the cultured DRGs neurons from one well of a 6-well plate were collected rapidly and pooled together to achieve enough RNA. Total RNA was extracted by the RNeasy Mini Kit (Qiagen, Valencia, CA) and treated with excess DNase I (New England Biolabs, Ipswich, MA). Highly purified, DNase-treated RNA samples from human DRG were purchased from Clontech Laboratories, Inc. (Mountain View, CA). RNA concentration was measured using the NanoDrop

2000 Spectrophotometer (Thermo Scientific, Wilmington, DE). Ratios of A260/280nm were between 1.97 and 2.08. RNA (500 ng) was reverse-transcribed into single-stranded cDNA using the Omniscript RT Kit (Qiagen) with specific RT-primers or oligo(dT) primers, and the cDNA template was amplified by real-time PCR using the primers listed in the Supplemental Table 3. Each sample was run in triplicate in a 20 μL reaction using SsoAdvanced Universal SYBR Green Supermix (Bio-Rad Laboratories, Hercules, CA). Reactions were carried out in a BIO-RAD CFX96 real-time PCR system. Ratios of RNA levels at different time points post-surgery (or post-injection) to RNA levels at 0 hours (or days) before surgery/injection, or of RNA levels in other treated groups to RNA levels in the control group were calculated using the ΔCt method ($2^{-\Delta\Delta Ct}$). All data were normalized to *Tuba1α*, which was stable even after peripheral nerve injury insult in mice as shown in our previous studies(23, 24, 44, 52).

Single-cell RT-PCR

The freshly dissociated DRG neurons from adult mice or donors were first prepared as described (23, 24, 43, 44, 52). Briefly, 4 hours (for mouse DRG culture) or 12-20 hours (for human DRG culture) after plating, single mouse living large (> 35 μm), medium (25-35 μm), and small (< 25 μm) DRG neurons (56) or single human living large (> 60 μm) and small (≤ 60 μm) DRG neurons (57) were sorted under an inverted microscope fit with a micromanipulator and microinjector and collected in a PCR tube with 6-8 μl of cell lysis buffer (Signosis, Sunnyvale, CA). After centrifugation, the supernatants were collected and divided into PCR tubes for different genes. The remaining RT-PCR procedure was performed according to the manufacturer's instructions with the Single-Cell RT-PCR Assay Kit (Signosis). All nest-PCR primers used are listed in Supplemental Table 3.

Rapid amplification of cDNA ends (RACE)

To determine the 5’ and 3’ end of both *NIS* V1 and *NIS* V2, the 5’ and 3’ RACE were carried out using a 2nd Generation 5’/3’ RACE Kit (Roche Diagnostics, Indianapolis, IN). For the 5’ RACE, the cDNA of each variant was first reversely transcribed from the total RNA of mouse DRG by strand-specific primers followed by poly(A) tailing and PCR amplification of the 5’-end of cDNA according to the manufacturer’s instructions. The 3’ RACE analysis was performed by reverse transcription of cDNA using oligo dT-anchor primer followed by gene-specific and anchor primer amplification. All primers are listed in Supplemental Table 3. PCR products from 5’ RACE and 3’ RACE were extracted, purified, and cloned to pCRBlunt II-TOPO vector (ThermoFisher) for DNA sequencing. All sequences were analyzed and the full-length *NIS* V1 and *NIS* V2 were determined.

RNA fractionation

Separation of nuclear and cytoplasmic fractions of primary cultured mouse DRG neurons followed by RNA isolation was carried out by using PARIS Kit (Invitrogen) (58). Briefly, after being rinsed with 1× DPBS, the cultured DRG neurons were lysed in ice-cold cell fractionation buffer. After incubation on ice for 10 minutes, the lysate was centrifuged for 1,000 × *g* at 4°C for 10 minutes to separate the nuclear and cytoplasmic fractions. Total RNA of each fraction was extracted by following the Kit instructions. Various gene/transcript expression levels in both nuclear and cytoplasmic fractions of all samples were quantified by quantitative real-time RT-PCR as described above.

Plasmid constructs and virus production

Full-length *Elf1, Fus, NIS* V1 and *NIS* V2 cDNAs were respectively amplified from mouse DRG RNA by using the SuperScript III One-Step RT-qPCR System with the Platinum Taq High Fidelity Kit (Invitrogen/Thermo-Fisher Scientific) and primers with restriction enzymes

(Supplemental Table 3). After double enzyme digestion, the PCR products were inserted into the corresponding sites of the pHpa-tra-SK plasmids (University of North Carolina, Chapel Hill) to replace enhanced GFP sequence or the multiple cloning site of the pAAV-MCS vector (Cell Biolabs, CA). The resulting vectors expressed the genes under the control of the cytomegalovirus promoter. The designed sense and antisense sequences for *Elf1* shRNA and *Fus* shRNA were annealed and inserted between the BamHI and XbaI sites of pAAV-shRNA-EF1a-EYFP. AAV5 packaging of viral particles carrying the cDNA was carried out using the AAVpro Purification Kit (Takara, Mountain View, CA). Virus titer was evaluated using the AAVpro® Titration Kit (Takara). AAV5-Cre and AAV5-*Gfp* were purchased from UNC Vector Core.

Northern blotting

To prepare complementary RNA (cRNA) probes of mouse *NIS* V1 and *NIS* V2, two PCR products with 400 bp and 452 bp fragments were amplified using mouse cDNA with a pair of primers including the T7 promoter at the 3' end (Supplemental Table 3) and identified using DNA sequencing. After PCR purification, a riboprobe was generated through *in vitro* transcription and labeled with digoxigenin-dUTP according to the manufacturer's instructions (Roche Diagnostics, Indianapolis, IN) at 37 °C for 2 h. The probes were purified using Micro Bio-Spin^{TM} 30 Chromatography Column (Bio-Rad).

Northern blot analysis was performed as described previously (24, 59). Briefly, the RNA (10 μg) extracted from injured DRGs 7 days after SNL was separated on an agarose/formaldehyde gel (2% for *NIS* V1 and 1% for *NIS* V2), transferred to a BrightStar-plus positively charged nylon membrane, and cross-linked using UV light. After pre-hybridization, the membrane was hybridized overnight at 68°C with a digoxigenin-UTP-labeled cRNA probe for *NIS-lncRNA*. The membrane was washed in a low salt buffer at room temperature for 2 × 5

min, high salt buffer at 68 °C for 2 × 5 min and SSC at 68 °C for 1 × 2 min. After being blocked, the membrane was incubated with alkaline phosphatase-conjugated sheep anti-digoxigenin (1:500, Roche) for 1 h at room temperature, and washed for 2 × 5 min, incubated by CDP-Star solution provided in the DIG Northern Starter Kit (Roche) and imaged by the ChemiDoc XRS System with Image Lab software (Bio-Rad). To clearly display the markers, the membranes loaded with the markers were separately imaged.

Luciferase reporter assay

A 693-bp fragment from the *NIS-lncRNA* promoter region (including the ELF1-binding motif) and a 1,664-bp fragment from the *Ccl2* promoter region (including FUS-binding sites) were amplified by PCR from genomic DNA using primers (Supplemental Table 3) to construct the *NIS-lncRNA* and *Ccl2* gene reporter plasmids, respectively, as described previously (24). The PCR products were cloned into the KpnI/HindIII or KpnI/NheI restriction sites of pGL3-Basic vector (Promega, Madison, WI). The accuracy of recombinant clones was verified by DNA sequencing. HEK-293T or CAD cells were plated on a 12-well plate and cultured at 37 °C in a humidified incubator with 5% CO_2. One day after culture, the cells in each well were co-transfected with 300 ng of plasmid (expressing full-length *Elf1*, *Fus*, *NIS* V1 or *NIS* V2), 300 ng of pGL3-Basic vector with or without the reporter plasmid, and 10 ng of the pRL-TK (Promega) using Lipofectamine 2000 (Invitrogen), according to the manufacturer's instructions. The wells were divided into different groups as indicated. Two days after transfection, the cells were collected and lysed in passive lysis buffer. Approximately 10 μl of supernatant was used to measure the luciferase activity using the Dual-Luciferase Reporter Assay System (Promega). Transfection experiments were repeated 3 independent times. Relative reporter activity was calculated after normalization of the firefly activity to *renilla*.

RNA sequencing

The ipsilateral L3/4 DRGs were harvested 5 weeks after microinjection with AAV5-*Gfp* or a mixture of AAV5-*V1* and AAV5-*V2* into the unilateral L3/4 DRGs of mice. Eight DRGs from four microinjected mice were pooled together to achieve enough RNA. Total RNA (1.2 μg/sample) extracted as described above was subjected to rRNA depletion by Ribo-Zero rRNA Removal (Human/Mouse/Rat) Kit (Illumina, San Diego, CA, USA). Strand-specific RNA libraries were prepared using TruSeq Stranded Total RNA Sample Preparation Kit (Illumina) without poly-A selection. All assays were performed according to the manufacturer's instructions. Sequencing was carried out using the Illumina HiSeq2500 platform High Output Mode, in a 2 x150 bp paired-end configuration, with a total of more than 190 M reads per lane (at least 60 M reads per sample) (22).

Basescope *in situ* hybridization and co-immunohistochemistry

Mice were deeply anaesthetized with isoflurane and transcardially perfused with 25-30 ml of 0.1M phosphate-buffered saline (PBS, pH 7.4) followed by 30-50 ml of 4% paraformaldehyde in 0.1 M PBS. Following perfusion, L4 DRG was harvested, post-fixed for 4-6 h at 4 °C in the same fixative and cryoprotected in 30% sucrose overnight. A series of 15-μm transverse sections were cut on a cryostat. The Basescope *in situ* hybridization (ISH) was carried out by using a protocol tailored to the BaseScope™ Detection Reagent Kit v2 with minor modification. The positive control probe for the housekeeping gene *Ppib* (REF: 320881, ACD) and the negative control probe for the bacterial gene *DapB* were used to validate the ISH procedure. Briefly, after the sections on the slides were treated with 0.3% Triton-X-100 in PBS at room temperature for 30 min, hydrogen peroxide was applied for 10 min at room temperature. The sections were then treated with Protease Plus (REF: 32330, ACD) for 15 min at 40° C, and incubated with either the

probe directed against mouse *NIS* V1 (REF: 720721, ACD) or *NIS* V2 (REF: 720731, ACD) for over two nights at 62° C. The signals were revealed following the manufacturer's instructions. Following ISH, the immunohistochemistry staining was performed as described previously(23, 44, 54, 55). The sections were incubated with chicken anti-β-tubulin III (1:200, catalog number: AB9354, EMD Millipore), rabbit anti-glutamine synthetase (1:500, catalog number: G2781, Sigma-Aldrich), rabbit anti-CD68 (1:100, catalog number: AB213363, Abcam) and rabbit anti-ATF3 (1:100, catalog number: HPA001562, Sigma-Aldrich) at 4°C overnight, respectively. The fluorescent signals were developed with appropriate fluorescence-conjugated secondary antibodies. The images were captured with a Leica DMI4000 fluorescence microscope (Leica). Immunoreactive positive neurons containing three or more particles of *NIS* V1 or *NIS* V2 were considered to be "co-expressed" cells, according to preceding studies(60, 61) and counted manually.

Bioinformatics prediction of transcription factors

The University of Santa Cruz (UCSC) genomic database (https://genome.ucsc.edu) was used to acquire the 2,000-bp promoter sequence of *NIS-lncRNA* gene. JASPAR database (http://jaspar.genereg.net/) was used to predictively analyze whether there were motifs of ELF1 in the promoter region of the *NIS-lncRNA* gene. The relative profile score threshold was set as 80%. Through analyzing the predicted score from the potential ELF1-binding regions, the region with the highest score was used to clone and to investigate the binding between ELF1 and *NIS-lncRNA.*

***In vitro* protein translation**

The full-length *NIS* V1 and *NIS* V2 DNA fragments containing the T7 promoter were obtained by RT-PCR with the primers listed in Supplemental Table 3 and were *in vitro*

transcribed and translated by the Transcend™ Non-Radioactive Translation Detection System procedures described in the manufacturer's manual (Promega, Madison, WI). In this system, biotinylated lysine residues were incorporated into nascent proteins during translation, allowing for *in vitro* non-radioactively-labeled protein synthesis. Proteins were detected by incubation with streptavidin-horseradish peroxidase and visualized using Western blotting procedure (62). *Luciferase* and *Creb1* were used as coding gene controls.

RNA pull down and mass spectrometry

The RNA-protein pull down assay was performed as described previously (44, 63). The cultured DRG neurons from one-month mice were prepared as mentioned above. Three days after culture, DRG neurons were rinsed with chilled PBS and collected in Pierce IP Lysis Buffer (Thermo Scientific). The biotinylated full-length sense *NIS* V1 or *NIS* V2 RNAs were synthesized by Biotin RNA Labeling Mix (Roche). The corresponding antisense RNAs were used as the negative controls. These RNAs were incubated, respectively, with the cell lysates overnight. The complex of RNA/protein was pulled down by Dynabeads MyOne Streptavidin T1 (Invitrogen). The collected proteins were solubilized in Laemmli sample buffer and separated by the 4–20% Mini-PROTEAN TGX Precast Protein Gels (Bio-Rad). Mass spectrometry experiments and data analysis were carried out in the Center for Advanced Proteomics Research, Rutgers New Jersey Medical School. Each gel lane was cut into five fractions, and in-gel trypsin digestion was performed. The resulting peptides were desalted with a C18 spin column and then analyzed by LC-MS/MS on a Q Exactive Mass Spectrometer. The MS/MS spectra were searched against the Swiss-Prot Mus Musculus database using a local MASCOT (V.2.4) search engine on the Proteome Discoverer (V1.4) platform. Through the analysis of Mass Spectrometry Proteomics, only those proteins pulled down by sense probes, but not negative control antisense

probes, were considered to be the positive targeted proteins. To verify the binding of *NIS-lncRNA* to FUS, Western blot analysis was carried out as described below.

Chromatin isolation by RNA purification

Chromatin isolation by RNA purification was carried out as reported (63, 64). Briefly, the DRG neuronal culture was prepared as described above. Three days after culture, the neurons were rinsed with chilled PBS and crosslinked by 1% formaldehyde for 10 min. The reactions were quenched by adding 0.125 M glycine for 5 min. After cell pellets were dissolved in nuclear lysis buffer, the cell lysates were sonicated to break DNA to 100-500 bp fragments. A total of 14 different biotinylated antisense DNA probes that were complementary to the sequence of *NIS-lncRNA* were designed using an online tool (Singlemoleculefish.com) and numbered. The negative control probes that were not complementary to the sequence of *NIS-lncRNA* were used as a negative control. Seven odd-numbered probes, seven even-numbered probes and negative control probes were hybridized, respectively, with the cell lysates overnight. The complex of beads/probes/DNA was pulled down by using streptavidin magnetic C1 beads (Invitrogen). The DNA was collected with pre-spin down yellow phase-lock gel tubes and the potential binding DNA was ready for analysis by quantitative PCR assay as described above.

RNA immunoprecipitation (RIP) Assay

The RIP assay was conducted using the Magna RIP Kit (Upstate/ EMD Millipore, Darmstadt, Germany) as described previously (44). The homogenates from mouse SNL or sham DRGs were suspended in the RIP lysis buffer containing the protease inhibitor cocktail and RNase inhibitor. The RIP lysate was incubated on ice for 5 min and stored at -80℃. The Magnetic Beads Protein A/G suspension for each IP was washed twice with the RIP wash buffer. Mouse anti-FUS antibody (2.0 µg; catalog number: AB154141, Abcam) or purified mouse IgG was conjugated to

Magnetic Beads Protein A/G re-suspended in RIP wash buffer for 30 min at room temperature. After being washed three times with RIP wash buffer, the Beads Protein A/G- antibody complexes were re-suspended into the RIP immunoprecipitation buffer. After being thawed and centrifuged at 14,000 rpm at 4℃ for 10 min, the supernatants of the RIP lysate were incubated with beads-antibody complex in the RIP immunoprecipitation buffer overnight at 4℃ by rotating. After the samples were washed six times with the RIP wash buffer, RNA was eluted from the beads by incubating in the proteinase K buffer at 55℃ for 30 min by shaking, purified by phenol/chloroform extraction and analyzed by quantitative RT-PCR as described above. The supernatant of the RIP lysate was used as Input. All primers used are listed in Supplemental Table 3.

Chromatin immunoprecipitation (ChIP) assay

Chromatin immunoprecipitation was performed using the EZ ChIP Kit (Upstate/EMD Millipore, Darmstadt, Germany) as described previously (23, 24, 65). Briefly, DRG homogenates were crosslinked with 1% formaldehyde for 10 min at room temperature. The reaction was terminated by the addition of 0.25 M glycine. After centrifugation, the collected pellet was lysed by SDS lysis buffer with a protease inhibitor cocktail and sonicated until the DNA was broken into fragments with a mean length of 200-1,000 bp. After the samples were pre-cleaned with protein G agarose, they were subjected to immunoprecipitation overnight with 2 μg of rabbit anti-ELF1 (catalog number: ORB315774, Abcam), mouse anti-FUS (catalog number: AB154141, Abcam), or purified rabbit IgG overnight at 4 °C. Input (10–20% of the sample for immunoprecipitation) was used as a positive control. The DNA fragments were purified and identified using PCR/quantitative real-time PCR with the primers listed in Supplemental Table 3.

Western blotting

To achieve sufficient protein, unilateral L3/4 DRGs from 2 CCI/sham mice, or unilateral L4 DRGs from 4 SNL/sham mice were pooled together. The tissues were homogenized and the cultured cells ultrasonicated in chilled lysis buffer (10 mM Tris, 1 mM phenylmethylsulfonyl fluoride, 5 mM $MgCl_2$, 5 mM EGTA, 1 mM EDTA, 1 mM DTT, 40 μ M leupeptin, 250 mM sucrose). After centrifugation at 4 °C for 15 min at 1,000 g, the supernatant was collected for cytosolic/membrane proteins and the pellet for nuclear proteins. The protein concentration in the samples was measured using the Bio-Rad protein assay (Bio-Rad) and the samples then heated at 99 °C for 5 min and loaded onto a 4–15% stacking/7.5% separating SDS-polyacrylamide gel (Bio-Rad Laboratories). The proteins were then electrophoretically transferred onto a polyvinylidene difluoride membrane (Bio-Rad Laboratories). After the membranes were blocked with 3% nonfat milk in Tris-buffered saline containing 0.1% Tween-20 for 1 h, they were incubated overnight at 4 °C with the following primary antibodies including goat anti-ELF1 (1:500, catalog number: PA5-18072, Invitrogen), mouse anti-CCL2 (1:500, catalog number: MA5-17040, Invitrogen), rabbit anti-FUS (1:1000, catalog number: AB154141, Abcam), rabbit anti-phospho-ERK1/2 (Thr202/Tyr204, 1:800, catalog number: 4370S, Cell Signaling), rabbit anti-ERK1/2 (1:800, catalog number: 4695S, Cell Signaling), mouse anti-GFAP (1:800, catalog number: 3670S, Cell Signaling), rabbit anti-GAPDH (1:2000, catalog number: G9545, Cell Signaling), and rabbit anti-histone H3 (1:1,000, catalog number: 17168-1-AP, Proteintech). The proteins were detected by horseradish peroxidase–conjugated anti-mouse secondary antibody (1:3,000, catalog number: 17168-1-AP, Jackson ImmunoResearch) or anti-rabbit secondary antibody (1:3,000, catalog number: 115-035-003, Jackson ImmunoResearch) and visualized by western peroxide reagent and luminol/enhancer reagent (Clarity Western ECL Substrate, Bio-

Rad) and exposed using the ChemiDoc XRS System with Image Lab software (Bio-Rad). The intensity of blots was quantified with densitometry using Image Lab software (Bio-Rad). All cytosolic/membrane protein bands were normalized to GAPDH and all nuclear proteins to histone H3.

Whole-cell patch-clamp recording

The acute disassociated neurons from the ipsilateral L3/4 DRGs of adult (8-10 weeks) wild type (WT) male mice or *Ccl2* knockout (KO) male mice with microinjection of AAV5-*Gfp* (*Gfp*) or a mixture of AAV5-*Gfp* and AAV5-*NIS* V1 (*V1*) into the unilateral L3/4 DRGs 4-5 weeks before tissue collection were cultured on laminin-coated coverslip as described (23, 24, 55, 65). Whole-cell patch clamp recording was carried out 4 to 6 h after plating to allow the recovery of cultured DRG neurons (23, 24, 55, 65). Only green-labeled neurons were recorded. Coverslips were placed in the chamber and were continuously perfused with 95% O_2/5% CO_2 bubbled with artificial cerebrospinal fluid (see extracellular solution) at 36 ℃. The artificial cerebrospinal fluid consisted of (in mM): NaCl 140, KCl 5, $CaCl_2$ 2, $MgCl_2$ 1, HEPES 10, and glucose 5, with pH adjusted to 7.3 by NaOH. The electrode resistances of micropipettes ranged from 3 to 5 MΩ. The neurons were voltage-clamped with an Axopatch-700B amplifier (Molecular Devices, Sunnyvale, CA). The intracellular pipette solution contained (in mM): potassium gluconate 120, KCl 20, $MgCl_2$ 2, EGTA 10, HEPES 10, Mg-ATP 4 (pH= 7.3 with KOH, 310 mOsm). The data were stored on computer by a DigiData 1550A interface and were analyzed by the pCLAMP 10.7 software package (Molecular Devices). According to the previous studies (23, 24, 66-69), manually identified small (< 25 μm), medium (25-35 μm) and large (> 35 μm) green-labeled DRG neurons were and recorded, respectively. The resting membrane potential was taken 3 min after a stable recording was first obtained. Action potential (AP) was evoked by delivering depolarizing currents. The membrane potential was held at the existing resting membrane potential during the current injection. The rheobase current was defined as the first step current which induced one AP by 50 ms depolarizing step current. Small and medium DRG neurons

were injected between 0 pA and 120 pA in 10 pA increments and large DRG neurons were injected between 100 pA and 600 pA in 50 pA increments, in current-clamp mode. The spike frequency versus injected current experiments were performed by measuring the average action potential firing rate during 500 ms depolarizing step current injection. Small and medium DRG neurons ranged between 20 pA and 160 pA in 20 pA increments and large DRG neurons ranged between 100 pA and 800 pA in 100 pA increments in current-clamp mode. The AP amplitude was measured between the peak and the baseline and the AP overshoot was measured between the AP peak and 0 mV. The after-hyperpolarization amplitude was measured between the maximum hyperpolarization and the final plateau voltage. The number of spontaneous APs was calculated as the number of APs per second in 3 min during extracellular buffer application.

Statistical analysis.

For *in vivo* experiments, mice were distributed into various treatment groups randomly. For *in vitro* experiments, the cells were evenly suspended and then randomly distributed into each well tested. The sample sizes were determined based on our pilot studies, previous reports in the field (24, 45, 70-72) and power analyses (power of 0.90 at $p < 0.05$). All of the results are given as means ± S.E.M. Data distribution was assumed to be normal but this was not formally tested. The data were statistically analyzed using two-tailed, paired Student's t-test and a one-way, two-way, or three-way ANOVA. When ANOVA showed a significant difference, pairwise comparison between means was performed using the *post hoc* Tukey method (SigmaPlot 12.5, San Jose, CA). Significance was set at $P < 0.05$.

References

43. Valtcheva MV, et al. Surgical extraction of human dorsal root ganglia from organ donors and preparation of primary sensory neuron cultures. *Nat. Protoc.* 2016;11:1877-1888.

44. Li Y, et al. N(6)-Methyladenosine Demethylase FTO Contributes to Neuropathic Pain by Stabilizing G9a Expression in Primary Sensory Neurons. *Adv. Sci. (Weinh.)* 2020; 7:1902402.

45. Tao,YX, et al. Impaired NMDA receptor-mediated postsynaptic function and blunted NMDA receptor-dependent persistent pain in mice lacking postsynaptic density-93 protein. *J Neurosci.* 2003;23:6703-6712.

46. Zhang Z, et al. Eukaryotic initiation factor 4 gamma 2 contributes to neuropathic pain through downregulation of Kv1.2 and the mu opioid receptor in mouse primary sensory neurones. *Br. J. Anaesth.* 2020;126(3):706-719.

47. Mailhot B, et al. Neuronal interleukin-1 receptors mediate pain in chronic inflammatory diseases. *J. Exp. Med.* 2020;217(9):e20191430.

48. Atianjoh FE, et al. Spinal cord protein interacting with C kinase 1 is required for the maintenance of complete Freund's adjuvant-induced inflammatory pain but not for incision-induced post-operative pain. *Pain* 2010;151:226-234.

49. Chu YC, et al. Effect of genetic knockout or pharmacologic inhibition of neuronal nitric oxide synthase on complete Freund's adjuvant-induced persistent pain. *Pain* 2005;119:113-123.

50. Cao J, et al. Short-term pre- and post-operative stress prolongs incision-induced pain hypersensitivity without changing basal pain perception. *Mol. Pain* 2015;11:73.

51. Wang PK, et al. Short-Term Sleep Disturbance-Induced Stress Does not Affect Basal Pain Perception, but Does Delay Postsurgical Pain Recovery. *J. Pain* 2015;16:1186-1199.

52. Rigaud M, et al. Species and strain differences in rodent sciatic nerve anatomy: implications for studies of neuropathic pain. *Pain* 2008;136:188-201.

53. Huang LN, et al. Fn14 Participates in Neuropathic Pain Through NF-kappaB Pathway in Primary Sensory Neurons. *Mol. Neurobiol.* 2019;56:7085-7096.

54. Li Z, et al. The transcription factor C/EBPbeta in the dorsal root ganglion contributes to peripheral nerve trauma-induced nociceptive hypersensitivity. *Sci. Signal.* 2017;10:eaam5345.

55. Mo K, et al. MBD1 Contributes to the Genesis of Acute Pain and Neuropathic Pain by Epigenetic Silencing of Oprm1 and Kcna2 Genes in Primary Sensory Neurons. *J. Neurosci.* 2018;38:9883-9899.

56. Pan Z, et al. Downregulation of a Dorsal Root Ganglion-Specifically Enriched Long Noncoding RNA is Required for Neuropathic Pain by Negatively Regulating RALY-Triggered Ehmt2 Expression. *Adv. Sci. (Weinh.)* 2021;8:e2004515.

57. Davidson S, et al. Human sensory neurons: Membrane properties and sensitization by inflammatory mediators. *Pain* 2014;155:1861-1870.

58. Han P, et al. A long noncoding RNA protects the heart from pathological hypertrophy. *Nature* 2014;514:102-106.

59. Gallagher PE, Diz DI. Analysis of RNA by northern-blot hybridization. *Methods Mol. Med.* 2001;51:205-213.

60. Samineni VK, et al. Cell type-specific modulation of sensory and affective components of itch in the periaqueductal gray. *Nat. Commun.* 2019;10:4356.

61. Steinkellner T, et al. Role for VGLUT2 in selective vulnerability of midbrain dopamine neurons. *J. Clin. Invest* 2018;128:774-788.

62. Baldwin A, Huh KW, Munger K. Human papillomavirus E7 oncoprotein dysregulates steroid receptor coactivator 1 localization and function. *J. Virol.* 2006;80:6669-6677.

63. Chu C, et al. Systematic discovery of Xist RNA binding proteins. *Cell* 2015:161:404-416.

64. Chu C, Quinn J, Chang HY. Chromatin isolation by RNA purification (ChIRP). *J. Vis. Exp.* 2012;61:e3912.

65. Liang L, et al. G9a participates in nerve injury-induced Kcna2 downregulation in primary sensory neurons. *Sci. Rep.* 2016;6:37704.

66. Biet M, et al. The neuronal potassium current IA is a potential target for pain during chronic inflammation. *Physiol Rep.* 2021;9:e14975.

67. Donnelly CR, et al. STING controls nociception via type I interferon signalling in sensory neurons. *Nature* 2021;591:275-280.

68. Kim YS, et al. Coupled Activation of Primary Sensory Neurons Contributes to Chronic Pain. *Neuron* 2016;91:1085-1096.

69. Stemkowski PL, et al. Increased excitability of medium-sized dorsal root ganglion neurons by prolonged interleukin-1beta exposure is K(+) channel dependent and reversible. *J. Physiol* 2015;593:3739-3755.

70. Li Z, et al. Dorsal root ganglion myeloid zinc finger protein 1 contributes to neuropathic pain after peripheral nerve trauma. *Pain* 2015;156:711-721.

71. Park JS, et al. Persistent inflammation induces GluR2 internalization via NMDA receptor-triggered PKC activation in dorsal horn neurons. *J Neurosci.* 2019;29:3206-3219.

72. Xu JT, et al. Opioid receptor-triggered spinal mTORC1 activation contributes to morphine tolerance and hyperalgesia. *J Clin Invest* 2014;124:592-603.

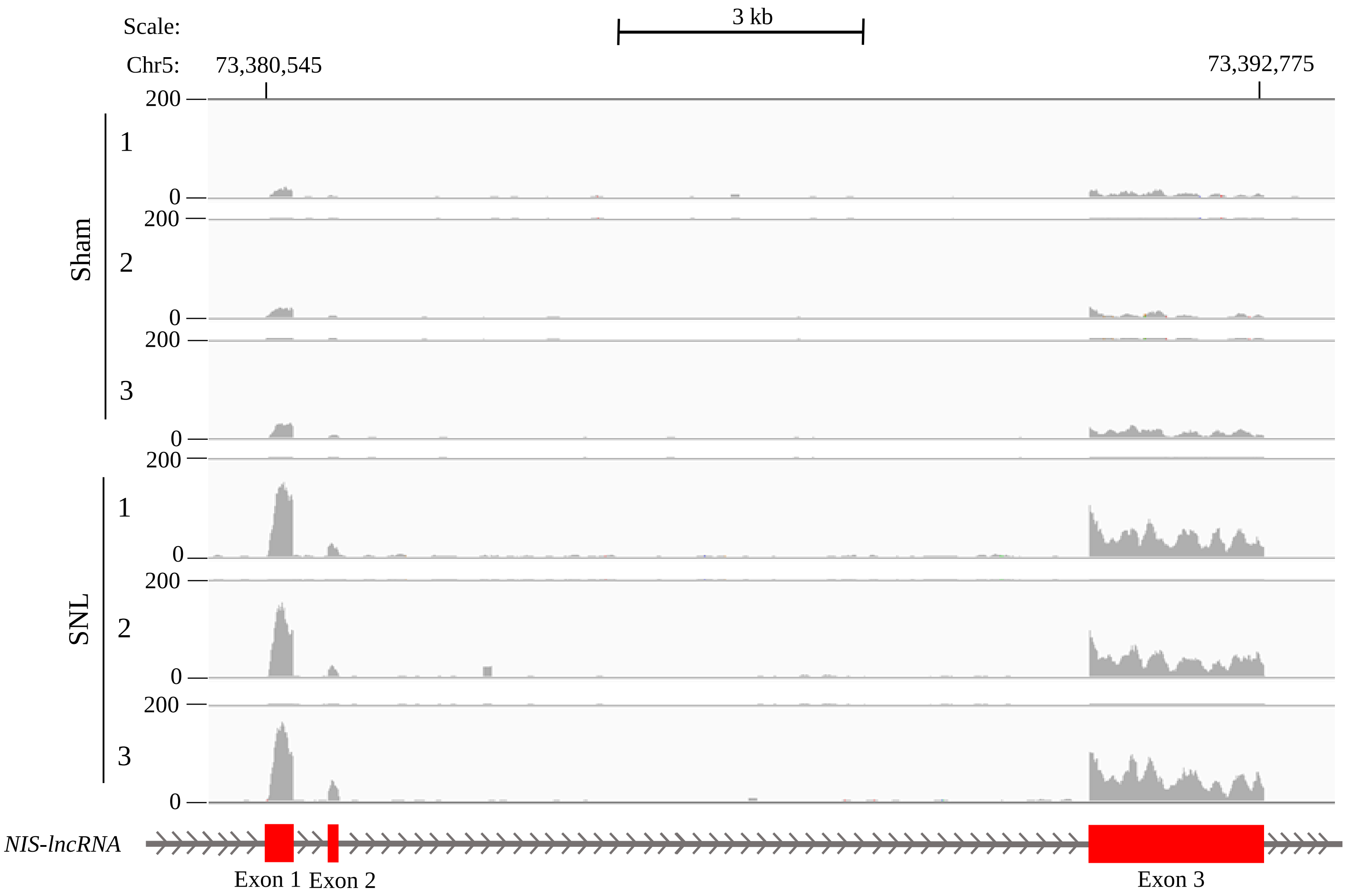

Supplemental Figure 1. The stacked reads in the exon regions of *NIS-lncRNA* gene in injured mouse dorsal root ganglion (DRG) after spinal nerve ligation (SNL) or sham surgery. Next-generation RNA-seq with a higher sequencing depth and without mRNA poly-A tail selection was carried out. The stacked reads were increased in the exon regions of *NIS-lncRNA* gene in the ipsilateral L4 dorsal root ganglia from SNL mice compared to those from sham mice on day 7 post-surgery. n = 36 mice (3 biological repeats)/group.

NIS V1

ACAAAGGGAACGCAGTGAGTGAGTAGAAGAGATTTTGGACTTAGTGGGTGAGGACATCCAAGAGAGCAAGGTACATGACGACGTGCAG
GAAAGATGAGCCAAGTAAAGAAGAGACTAGAACAACGCTGTGCTAGACTTTCCAGGCTCTGGGGACAGCTGAGGCTAGAACGATGAGA
AAGAGAGAGGAGTGAGCCAGAAGAGGAGAGAGACAAGGGGAAGGGGAGTCTGCCGAGGAAGGATGAAGACAGAGGGAAGCATAGTTG
GGAAGGAGAAGCGCTATAAAACCCAGGAGCAAAGCAGACTGGGCTTGTAGCAGAAGGCAGAATAGCTTGGCAGCTATGTGACAGACAT
GATGCTGAGCTTGGCTACGTCACTGACACCGTGACTTCAGGTTTCCAGCCCTGTGACAGACATCTAAAATAAACAGCATGCATA

NIS V2

TCACCAAGACGGGCAAATCACAAAGGGAACGCAGTGAGTGAGTAGAAGAGATTTTGGACTTAGTGGGTGAGGACATCCAAGAGAGCAAG
GTACATGACGACGTGCAGGAAAGATGAGCCAAGTAAAGAAGAGACTAGAACAACGCTGTGCTAGACTTTCCAGGCTCTGGGGACAGCTGA
GGCTAGAACGATGAGAAAGAGAGAGGAGTGAGCCAGAAGAGGAGAGAGACAAGGGGAAGGGGAGTCTGCCGAGGAAGGATGAAGACAG
AGGGAAGCATAGTTGGGAAGGAGAAGCGCTATAAAACCCAGGTGCTGCGGGAGCCACAGGTTACTGTGCAGATGTGGCTAAGTCCAAGAA
CCACACGATACACAACCAGTCCAGCAAATGGCACAGAAATGGCATCAAGAAACCCAGGTCACAAAGATATAAATATCTTAAGGGGGCTGA
CCCCAAGTTCCTGAGGAACATGCGCCTTGCCAAGAAGCACAACAAGAAAGGCCTGAAGAAGATGCAGGGCAACAATGCAAAGGCAGTGA
GTGCGCACGCAGAGGTCATCAAGGCCCTGGTGAAGCCTCAGGCCATCGAGCCCAAGATGCCAAAGGGCCCAGCCACAGACTCAGCGGGCT
GGCTTTCATCACTCACCCCAAGCTTGGGAAGCAGATTTGAAGCTACATGGCCAAGGGTGTAGGCTCTGCCAACTAAAGCCCAAGGCAGAGG
CCACAGCTCCAGCTAAGGGCCAGGCTTTAGCTCCAGCCCAGGCTCGCAAAGGTGCGCAGGCCCCTGTGAAGGTCCCATGGAAAAGGCTCCT
GCCAGTGTGAAGACAGATGGACTGCTGTGACACACCTACCTATACACTGTTTGCAGATGACCAGTGACCTATGCTGTTTTTACAAATAAACT
TGAGGCAAGATCTAACACACACACACACATACACACACACACACACACACACACACACACACACACGCACACACGCACGCACGCACAC
ATGCACACACGCACACACACATTTATTTTGGAGAAAAGGTCTCACCATGCAACCCAGGAAAGTCTGGAACTCACTATGTACTCCAGACTGG
CTGTGAGCTTATGAAACTCCTGCCTCAGCACCTTGAACACTGTGACTAAAGATGTGCACTATGAAGTTTTAATTCTCTCAGCTGGTTTTCAG
CAAGCGTTCACAATGAGTTTCACTGTGGGTGTGGACTGCATACACAACACTGTTTTCTGTAAAATCTTGATGATTAAAAATAAAAGCATATA
ACTCCTCACTAGAAAGATTTCAGGCCAGCACTGGAAATGTTTTGTTTGTTCCTTCGCTGGCTTTTGTTTTTACTTAGATCTATGATTAAAAGG
AAGTGAATGTTGATGAAAAACCTGACTCAGGGCTTCACTGAGCAGCTACAGTGAATTCATAGAATAGTCATTGTCTGCGTGCTGGCCGCGT
TTGTGCAGTCACTGTAGAATGACGACGGTACACCCCTAACAATGTTCACAAGAGAAACAGTTCTGAGGGGTTTCTTTCGTAGTTCCTGAATC
AGACAGCCATGTCCCAATAGCAGTACTTTAACTCCTGTGCCTTGGTGCTGGGGGCAAAGAGAGTGGCCCTCTCGGAACACCAGCTCGTGGA
CTGCATCACACCTGGTCTGAAGATGAACTTGGAAGGCGCTGCATTTGACGTCACGTGGAGCCAAGCGTCTACCACTGCCTCTCTCAACTGTG
GCTGGCTGTTCAAAATCTTTACTTCTGACTTAGCGGCTCCAGTTCTCATGGTTCTAAGTCGTGGGATCTCCATTGTGCGAGAGATCAGGTATC
CCCTGAAAGGGACTTGACAATAAATGTGGTATTCACTGTTAATGTTCTTTTCGCTGGATCTCCTCCAACTTGGTGCCAAAGAATATTTGAGA
GTTGTTGGGCTGTGTATCTAAAGGTTTCTGTTGGGCCTGCGATTTGTATCAGAAATAATTTATATTGAAGATGGAGAAACACAGACACTAAAA
GACACTTTTGAGACTGATTCAAGAGAAAGGCATTTTGAAATGCCCCATGTCCTGAATCTTCACTTAGTGCACATGTGCTGTGTGCATGCCTC
ATGCAGCCATGCGCTAGGGAGCTCTCTGGGTACCTAGCACCCGGCACCCGGCATCCATCATCCATCATCCATCATCCAACGAGGCCAGCAT
CACGGTGTGCCCTTGATGTGCCTTCAAACCATGCAAAGGACTCAGATTCACCTACAGCCTCTGGCTCCCCCTCTGAAAGGGCACAAAGAATT
CTTCCTAGAGCAAAGTCACTCCTCTCCTGCTCTGCCTTGGAGAGAGGTCAGAGGTCACGATGGTCAGAGGCTCTTCTTTCCTTCAGTCTGTTC
AGGTGTCAGTGTTCTGGCTGAAGCACCCCACCCCCCCTGTTCTCGCTTTTGTCACATATCTCTCTAGGATATTTGCCTTTAAAATCTTGACCA
CTTCTTA

Supplemental Figure 2. Full-length cDNA sequences of mouse *NIS-lncRNA*. Two splice variants: splice variant 1 (*NIS* V1, 435 nt) and variant 2 (*NIS* V2, 2,469 nt) cDNAs. Two exons are presented in different colors. Translation analysis with DNAMAN software shows many stop codons (underlined TAG, TGA, TAA) distributed throughout the sequences. Genomic location: Chr5: 73380564 – 73381411 for *NIS* V1 and Chr5: 73380545 – 73392775 for *NIS* V2. n = 3 repeats (mice).

AAGGGGAATGTGGTAGGTGAGCAGGAGAGATTTTGGACTTAGCGGGTGAGGACATTGAAGAGACCTAGGTACT
TGACGAAGTTCAGGAAAGATGATCCGAGCAAAGAAGAGACTAGAACAATTCGCTGCTGGAATTTCCAGGACCT
GGGGAACAGCGAAGTTAGAATGATGAGAGAGAGAGAGAGGAGTGAGCCCAAGAAGGAGAGAGACAGGGGTG
TCTGAAGAGGAAGGATGAAGACTTGGGAGAGGGGAGTATAGTTGGTTAGTAGAGTGGCTGTAAAACCCAGACA
AAGCAGACTGGTCTTGTAGCAGAAGGCAGAATAGCTTGGCAGCTATGTGACAGACAGGCTGCTGAACTTGGCT
GTGTCACTGACACTGTGACTTCAGGTTTCCAGCACTGTGACAGACATCTAAAATAACCAGCATGCA

Supplemental Figure 3. Full-length cDNA sequence of rat *NIS-lncRNA* (429 nt). Two exons are presented in different colors. Translation analysis with DNAMAN software shows many stop codons (underlined TAG, TGA, TAA) distributed throughout the sequences. Genomic location: Chr14: 34826164-34826450. n = 3 repeats (rats).

TTGCAAAGAGCCTATAGGAGGAAACAGATACATCAGAAAGGTAACAAAATACACTACGTGATACTGGTTCCA
ACTACTGAGGAGAGTGTGTGGAGCCTTGACTTGAGGGCTGGGAGAAGCTTCACCAATGTCACAAATAACTTT
TGAACTTGGCTTTGAAGTGGGCTTCTTCTGCTCAGATAAGAAAGAATTACATTGACAAAGGTGAGGAGGAAG
TAGACATAGAGAAAATTAGCTAATATTTAAAGAGTTCTAAACACCCTATTTGGTACGCAGTAGATACTCTGTAA
ATATTTATTCAGTGAATAAGCATCCTTGTTAGAAAAATGTCATTGTAGCCATTTTGCAGATGAAGAAATGGAGG
CACAGACAGATTGTTCTAAAGTCACGTAGCTAGAAGTTGTGGAATTAGATATTGACTCCTAAGTTGCCTGCCT
AACAAGCTTTATGTTCTTTCCCTTGTCCAGATGTGTGGCGAAAGTGCTCACATGTGGTATAAAGTATCAGATGC
CTAGGTGAGGGAGTGGAGGGGGGTGTTATGGCCAGAGGTGAACTTAGAAACACAAGTCAGAATGGACTATA
GCAGTCCTTAAGATGACATTCTAGGGAACATGGAAGGTCATTTAAGGAAGCTACCAAAGATCTTAAAGTAGGG
GAATGATGTCATCAGTCTGTCTTTCAGGAAGATCAGTGTAATAGGATGGAACTTGCAGGCAGGGGTGGGTGC
GGGAGTGATGAGTGTAGGCTGATGGGCAAGTTAGGAAACGCTATTGCTCAGAGCTAATATGGATGAATAGGAG
ACTGAGCCAGGATAGTGGCAGTGGAAATGGAGAAGAAGAGGCATAAGGACTGAAAACATATTAGAGGAAGA
GTCACCACCACTCATTGACCAAATGGATTTGAGGGAAGGGAAGGGAAGGGAAGGGGTGCAAGATGGTGCTG
GGATTGCCAGCCTTGGTGAATGACGATGCCCTTAGCTGAAACAAAGGATGGAAGAAAATGCAGTCAGTGGGC
AGAAGACAATGTTTTGGACATTTTGAGTGAGACATCCAAAGTGAGATGTATAATGGACAGGGGGAGATATAAG
ATAAAAACATAGAAGAGCAACTGGAATAAGAGAATGTTGGGAATCATCAGCCTCTAAGGGACAGCTGAGGCC
GGAAGGATGAGAGGACCAGGGAAAGGTGTAGGAGAGAAGAGAATGGGGTGGGAAAAGGAGGGGAGGGAG
GGGAAGGCATGGGTAGAGGAAAAAGAATCAGCAAA

Supplemental Figure 4. Full-length cDNA sequence of human *NIS-lncRNA* (1,263 nt). Translation analysis with DNAMAN software shows many stop codons (underlined TAG, TGA, TAA) distributed throughout the sequences. Genomic location: Chr4: 48958094 –48959356. n = 3 repeats (donors).

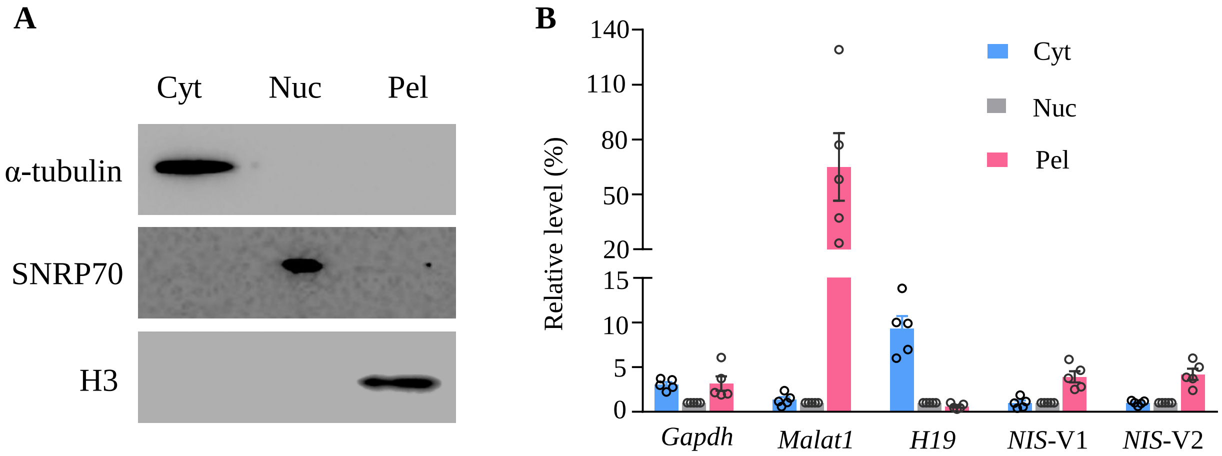

Supplemental Figure 5. Subcellular distribution of *NIS*-V1 and *NIS*-V2 in the dorsal root ganglion (DRG). (**A**) Identification of cytoplasmic soluble (Cyt), nuclear soluble (Nuc) and insoluble pellet (Pel) fractions, which fractionated from the cultured DRG neurons of adult mice. α-tubulin: a marker of cytoplasmic soluble fraction. SNRP70: a marker of nuclear soluble fraction. H3: a marker of nuclear insoluble pellet fraction. (**B**) Relative levels of *Gapdh* mRNA, *Malat1*, *H19*, *NIS*-V1 and *NIS*-V2 expression in cytoplasmic soluble (Cyt), nuclear soluble (Nuc) and insoluble pellet (Pel). n = 5 mice.

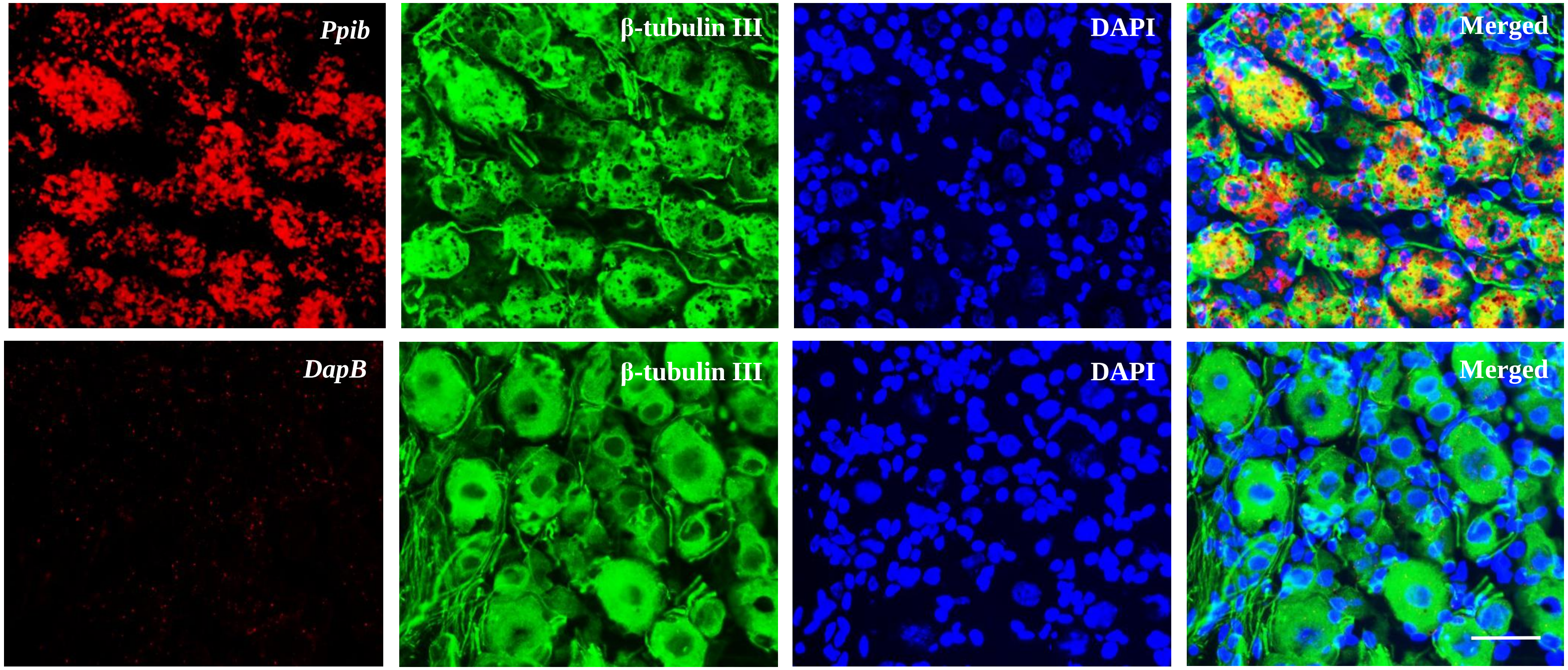

Supplemental Figure 6. Positive and negative controls for BaseScope *in situ* hybridization (ISH) assay. A housekeeping gene *Ppib* (peptidyl-prolyl cis-trans isomerase B) expressed in any eukaryote was used as a positive control, whereas a bacterial gene *DapB* (4-hydroxy-tetrahydrodipicolinate reductase) was used as a negative control. Top panels: Representative images show that *Ppib* signals (red) by BaseScope co-express with β-tubulin III (green, a specific neuronal marker) in individual dorsal root ganglion neurons from naive mice. Bottom panels: Representative images show no signals of *DapB* by BaseScope, but display β-tubulin III immunostainings in individual dorsal root ganglion neurons from naive mice. Cellular nuclei are co-labeled by 4', 6-diamidino-2-phenylindole (DAPI, blue). n = 3 mice. Scale bar: 50 μm.

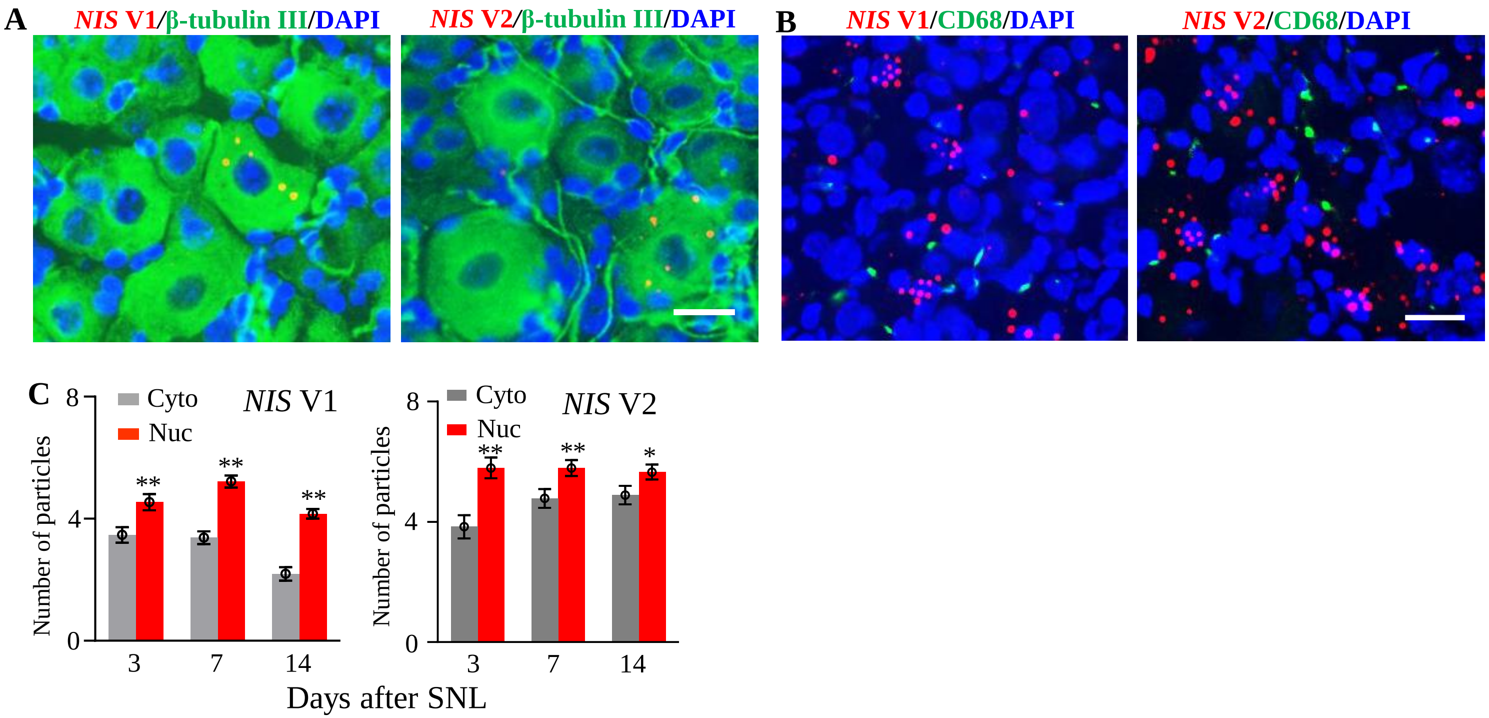

Supplemental Figure 7. *NIS* V1 and V2 are expressed in the DRG neurons from naive and SNL mice. (**A**) Representative photomicrographs showing the expression of *NIS* V1 and V2 in the L4 DRG from naive mice. Signal particles *NIS* V1 (red, left) and V2 (red, right) are undetected or sparsely detected in the neurons. (**B**) Representative photomicrographs showing that signal particles of *NIS* V1 (red, left) and V2 (red, right) are undetected in the macrophages marked by Cluster of Differentiation 68 (CD68, green) in the ipsilateral L4 DRG on day 7 post-SNL. Cellular nuclei are labeled by 4′, 6 diamidino-2-phenylindole (DAPI, blue). (**C**) Numbers of nuclear (Nuc) particles and cytoplastic (Cyto) particles per *NIS* V1- or *NIS* V2-labeled neuron from the ipsilateral L4 DRG on days 3, 7 and 14 post-SNL. n = 70, 76 and 67 neurons for *NIS* V1 and n = 68, 82 and 73 neurons for *NIS* V2 from the ipsilateral L4 DRG on days 3, 7 and 14 post-SNL, respectively. n = 5 mice/time point. $*P < 0.05$ or $**P < 0.01$ versus cytoplasm at the corresponding time points by two-way ANOVA with post hoc Tukey test. Scale bar: 25 µm.

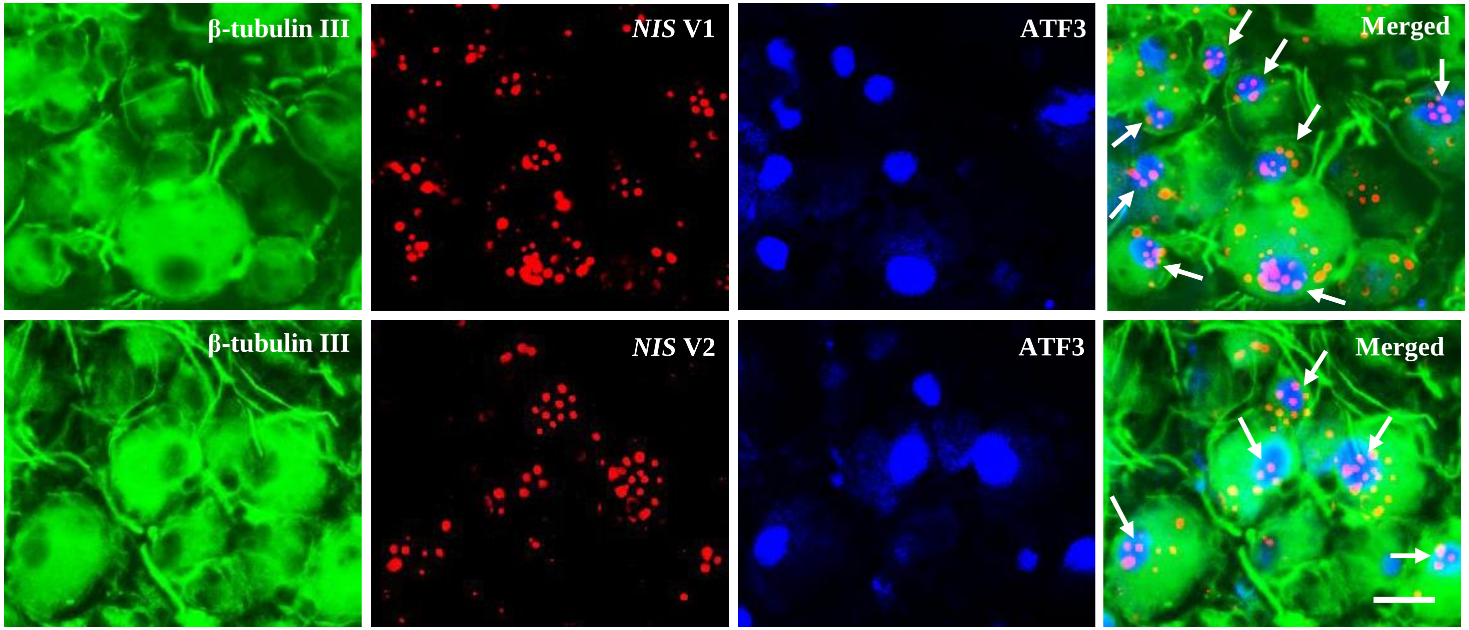

Supplemental Figure 8. *NIS* V1 or V2 is expressed in most injured DRG neurons under neuropathic pain conditions. Representative photomicrographs showing that *NIS* V1 (red) or *NIS* V2 (red) was co-expressed with ATF3 (blue, an injury marker) in individual neurons (marked by β-tubulin III) of DRG 7 days after SNL. Arrows: signal particles (≥ 3) in neuronal nuclei. n = 3 mice.

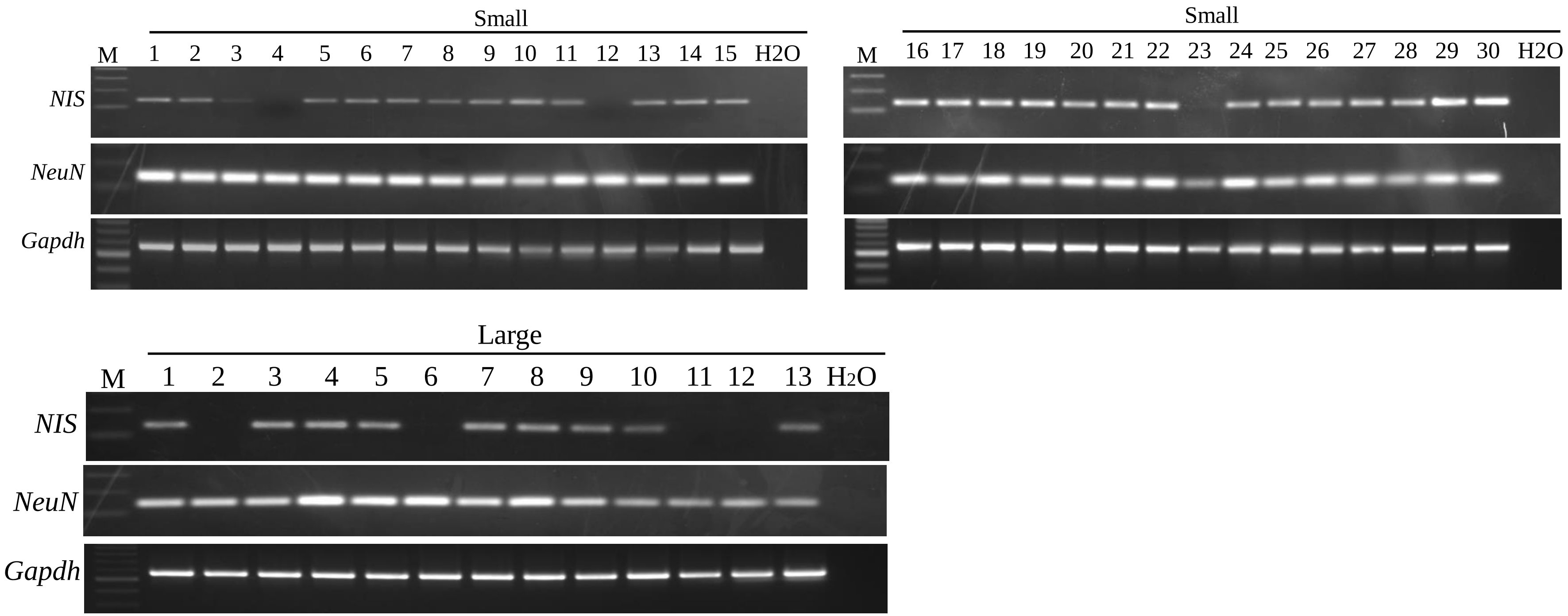

Supplemental Figure 9. Expression analysis of *NIS-lncRNA* (*NIS*) in individual neurons of human dorsal root ganglion (DRG) by single-cell RT-PCR assay. *NeuN* mRNA is used as a marker for DRG neurons. *Gapdh* mRNA is used as a loading control. Approximately 86.7 % (26/30) of small DRG neurons (≤ 60 µm) and 69.2 % (9/13) of large DRG neurons (> 60 µm) are positive for *NIS-lncRNA*. n = 3 donors. Number 1–13/15 or 16-30 indicates different neurons. M: DNA ladder marker. H_2O, no cDNA, is a negative control.

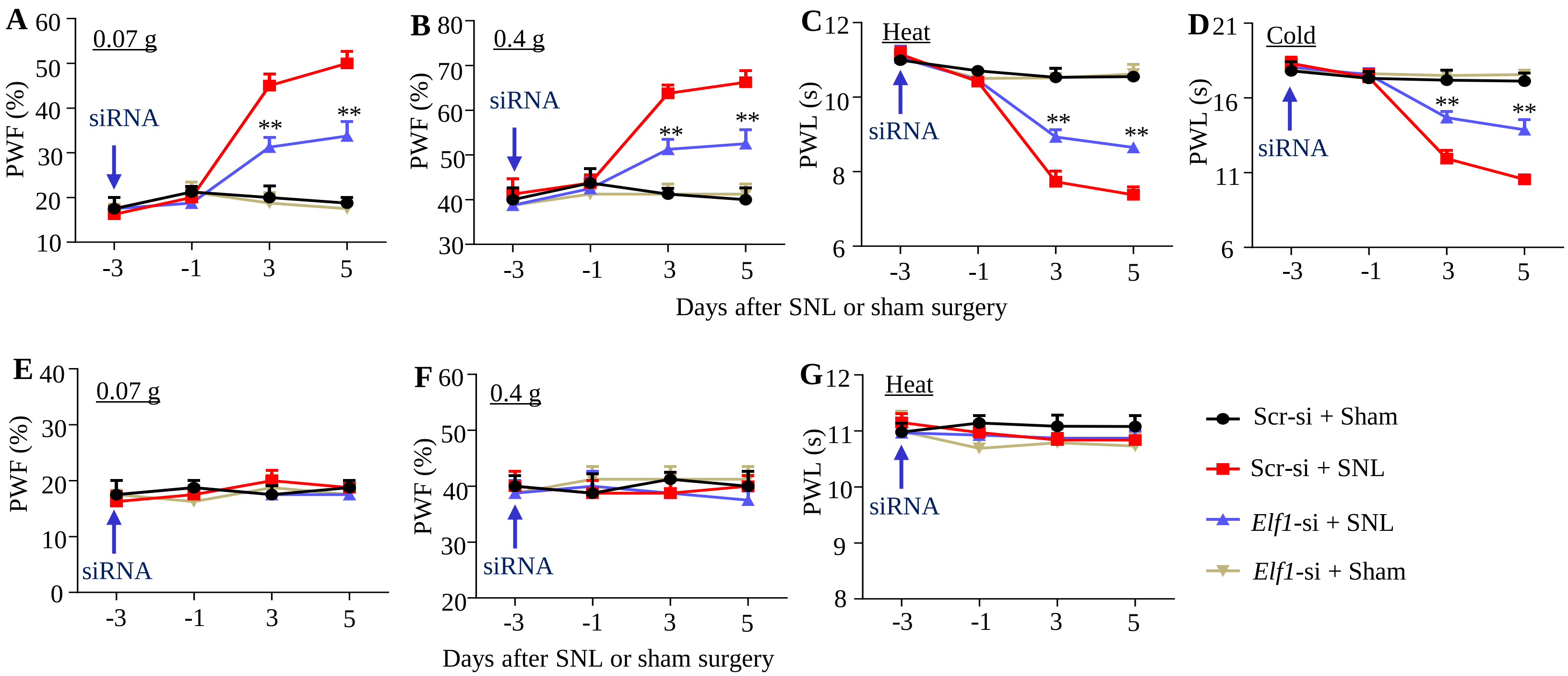

Supplemental Figure 10. Blocking nerve injury-induced increase in DRG *Elf1* attenuated the development of SNL-induced nociceptive hypersensitivity in male mice. (**A-G**) Effect of microinjection of *Elf1* siRNA (*Elf1*-si) or control scrambled siRNA (Scr-si) into the ipsilateral L4 DRG 3 days before surgery on the paw withdrawal frequency (PWF) to 0.07 g (A and E) and 0.4 g (B and F) von Frey filaments and on paw withdrawal latencies (PWL) to heat (C**,** G) and cold (d) stimuli on the ipsilateral (A-D) and contralateral (E-G) sides at the different days after SNL or sham surgery. n = 8 mice/group. ***P* < 0.01 versus the scrambled siRNA plus SNL group at the corresponding time points by three-way ANOVA with repeated measures followed by post hoc Tukey test.

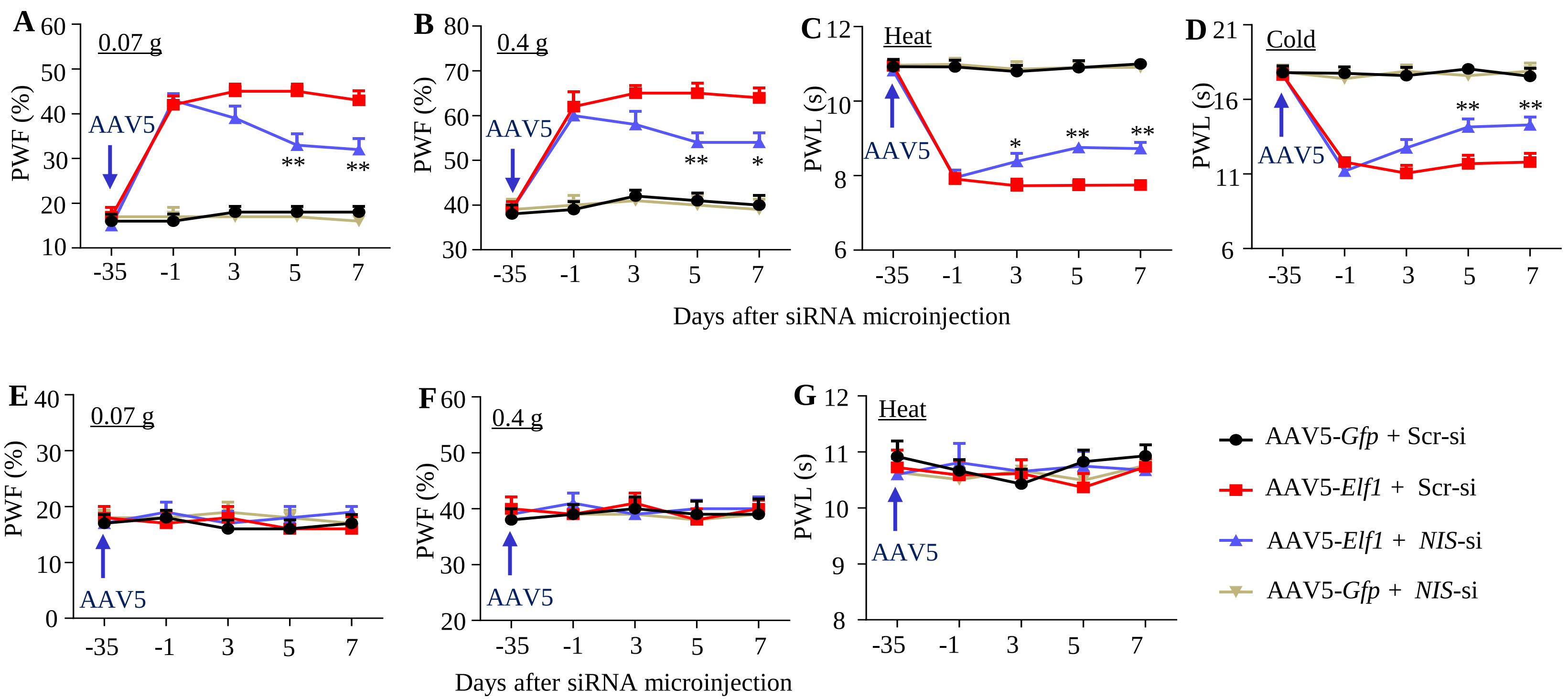

Supplemental Figure 11. DRG *NIS-lncRNA* mediated nociceptive hypersensitivities caused by DRG overexpression of *Elf1* in male mice. (**A-G**) Effect of microinjection of *NIS-lncRNA* siRNA (*NIS*-si) or control scrambled siRNA (Scr-si) into ipsilateral L3/4 DRGs on paw withdrawal frequency (PWF) to 0.07 g (A and E) and 0.4 g (B and F) von Frey filaments and on paw withdrawal latencies (PWL) to heat (C and G) and cold (D) stimuli on the ipsilateral (A-D) and contralateral (E-G) sides at time points as shown in the mice with microinjection of AAV5-*Elf1* or AAV5-*Gfp* into unilateral L3/4 DRGs 35 days before siRNA microinjection. n = 10 mice/group. **P* < 0.05, ***P* < 0.01 versus the AAV5-*Elf1* plus Scr-si group at the corresponding time points by two-way ANOVA with repeated measures followed by post hoc Tukey test.

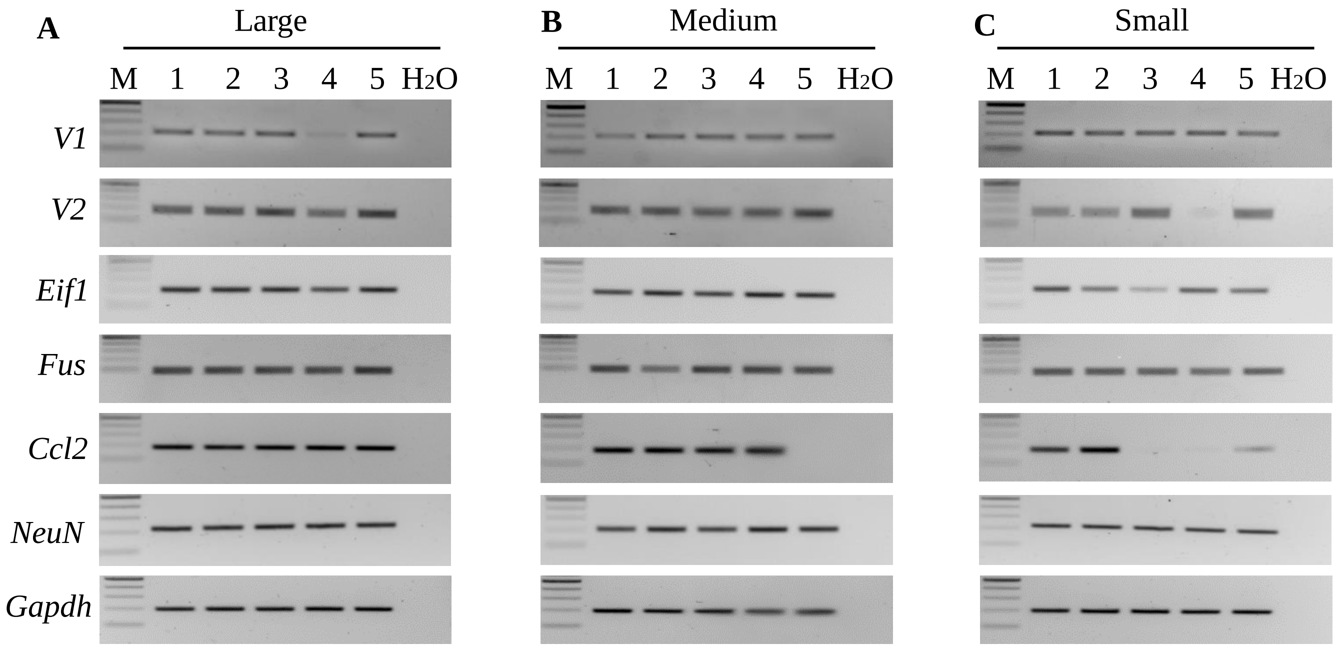

Supplemental Figure 12. Co-expression analysis of *NIS-lncRNA* V1 (*NIS* V1), *NIS* V2, *Elf1* mRNA, *Fus* mRNA and *Ccl2* mRNA in individual neurons from injured dorsal root ganglion (DRG) 7 days after CCI by single-cell RT-PCR assay. *NeuN* mRNA is used as a marker for DRG neurons. *Gapdh* mRNA is used as a loading control. (**A**) Co-localization of *NIS* V1 and *NIS* V2 with *Elf1*, *Fus* and *Ccl2* mRNAs in four of 5 individual large DRG neurons (> 35 µm in diameter). (**B**) Co-localization of *NIS* V1 and *NIS* V2 with *Elf1*, *Fus* and *Ccl2* mRNAs in four of 5 individual medium DRG neurons (25-35 µm in diameter). (**C**) Co-localization of *NIS* V1 and *NIS* V2 with *Elf1*, *Fus* and *Ccl2* mRNAs in three of 5 individual small DRG neurons (< 25 µm in diameter). n = 5 neurons/size. Number 1–5 indicates five different neurons. M: DNA ladder marker. H_2O, no cDNA, is a negative control.

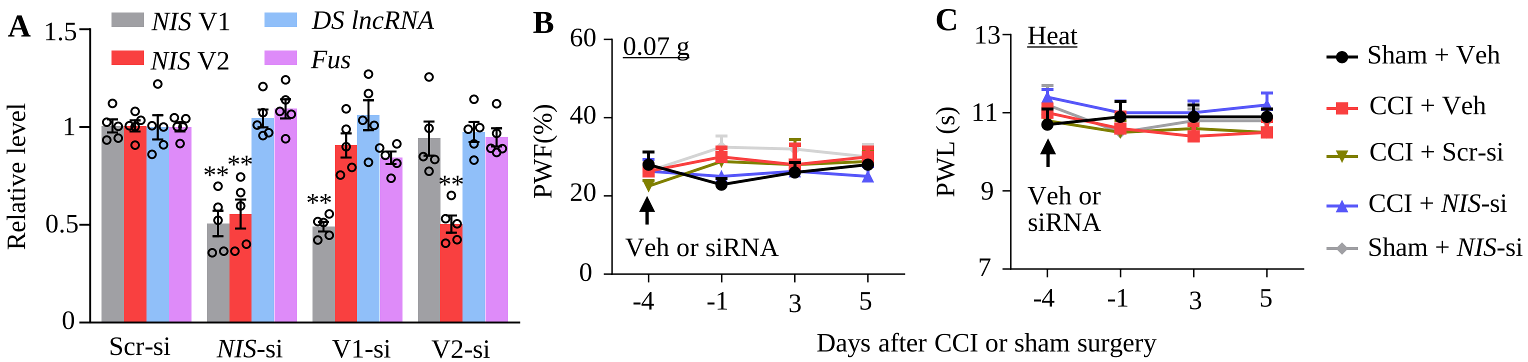

Supplemental Figure 13. Specific effect of *NIS-lncRNA* siRNA, *NIS* V1 siRNA and *NIS* V2 siRNA on *NIS* V1 and *NIS* V2 expression and effect of *NIS-lncRNA* siRNA on contralateral paw withdrawal responses in male mice. **(A)** Levels of *NIS* V1, *NIS* V2, *DS-lncRNA (DRG-specifically enriched long noncoding RNA)* and *Fus* mRNA in the cultured DRG neurons 2 days after transfection of *NIS-lncRNA* siRNA (*NIS*-si), *NIS* V1 siRNA (V1-si) , *NIS* V2 siRNA (V2-si) or control scrambled siRNA (Scr-si). n = 5 biological repeats/group. **P* < 0.05, ***P* < 0.01 versus the corresponding control scrambled siRNA group by one-way ANOVA with post hoc Tukey test. **(B** and **C)** Effect of microinjection of *NIS* siRNA (*NIS*-si), scrambled siRNA (Scr-si) or vehicle (Veh) into the ipsilateral L3/4 DRG 4 days before surgery on basal paw withdrawal frequency (PWF) to 0.07 g von Frey filament (B) and basal paw withdrawal latency (PWL) to heat stimulation (C) on the contralateral side at days as indicated post-CCI or sham surgery. n = 10 mice/group. Three-way ANOVA with repeated measures followed by post hoc Tukey test

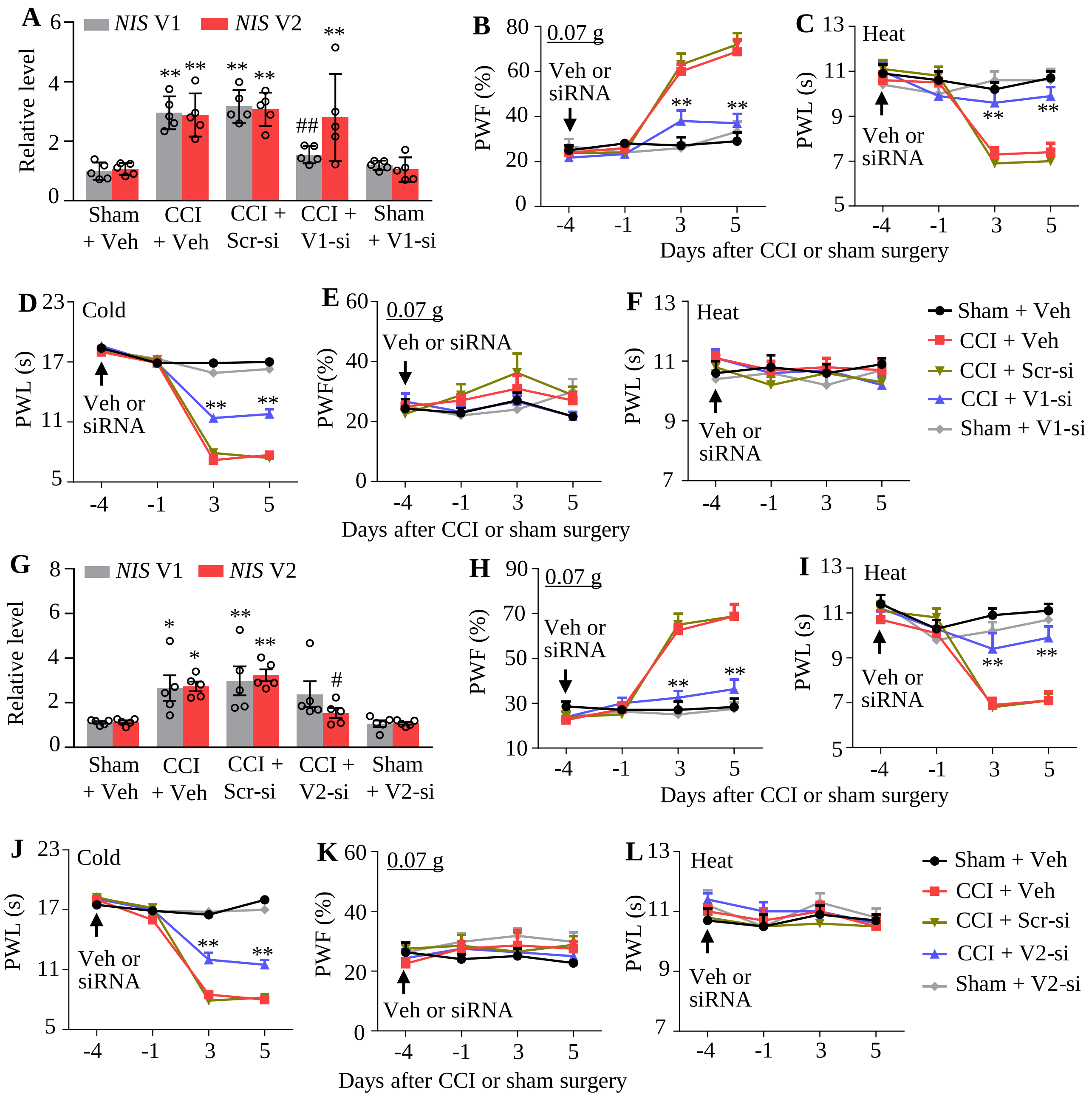

A
NIS V1
NIS V2
Relative level
Sham + Veh
CCI + Veh
CCI + Scr-si
CCI + V1-si
Sham + V1-si
B
0.07 g
Veh or siRNA
PWF (%)
C
Heat
PWL (s)
Days after CCI or sham surgery
D
Cold
E
PWF(%)
F
Sham + Veh
CCI + Veh
CCI + Scr-si
CCI + V1-si
Sham + V1-si
G
CCI + V2-si
Sham + V2-si
H
I
J
K
L

Supplemental Figure 14. Blocking the CCI-induced increases of *NIS* V1 or *NIS* V2 in injured DRG alleviated the development of CCI-induced nociceptive hypersensitivities in male mice. **(A)** Levels of *NIS* V1 and *NIS* V2 in the ipsilateral L3/4 DRG on day 5 after CCI or sham surgery in the mice with microinjection of vehicle (Veh), *NIS* V1 siRNA (V1-si) or control scrambled siRNA (Scr-si) 4 days before surgery. n = 10 mice/group. **P < 0.01 versus the corresponding sham plus vehicle group and ##P < 0.01 versus the corresponding CCI plus scrambled group by two-way ANOVA followed by post hoc Tukey test. **(B-F)** Paw withdrawal frequency (PWF) to 0.07 g von Frey filament (B and E) and paw withdrawal latencies (PWL) to heat (C and F) and cold (D) stimuli on the ipsilateral (B-D) and contralateral (E-F) sides at the different days as indicated after CCI or sham surgery in the mice with microinjection of vehicle (Veh), *NIS* V1 siRNA (V1-si), or control scrambled siRNA (Scr-si) 4 days before surgery. n = 10 mice/group. **P < 0.01 versus the CCI plus control scrambled group at the corresponding time points by three-way ANOVA with repeated measures followed by post hoc Tukey test. **(G)** Levels of *NIS* V1 and *NIS* V2 in the ipsilateral L3/4 DRG on day 5 after CCI or sham surgery in the mice with microinjection of vehicle (Veh), *NIS* V2 siRNA (V2-si), or control scrambled siRNA (Scr-si). n = 10 mice/group. *P < 0.05, **P < 0.01 versus the corresponding sham plus vehicle group and ##P < 0.01 versus the corresponding CCI plus scrambled group by two-way ANOVA followed by post hoc Tukey test. **(H-L)** Paw withdrawal frequency (PWF) to 0.07 g von Frey filament (H and J) and paw withdrawal latencies (PWL) to heat (I and L) and cold (J) stimuli on the ipsilateral (H-J) and contralateral (K-L) sides at the different days as indicated after CCI or sham surgery in the mice with microinjection of vehicle (Veh), *NIS* V2 siRNA (V2-si), or control scrambled siRNA (Scr-si) 4 days before surgery. n = 10 mice/group. **P < 0.01 versus the CCI plus scrambled group at the corresponding time points by three-way ANOVA with repeated measures followed by post hoc Tukey test.

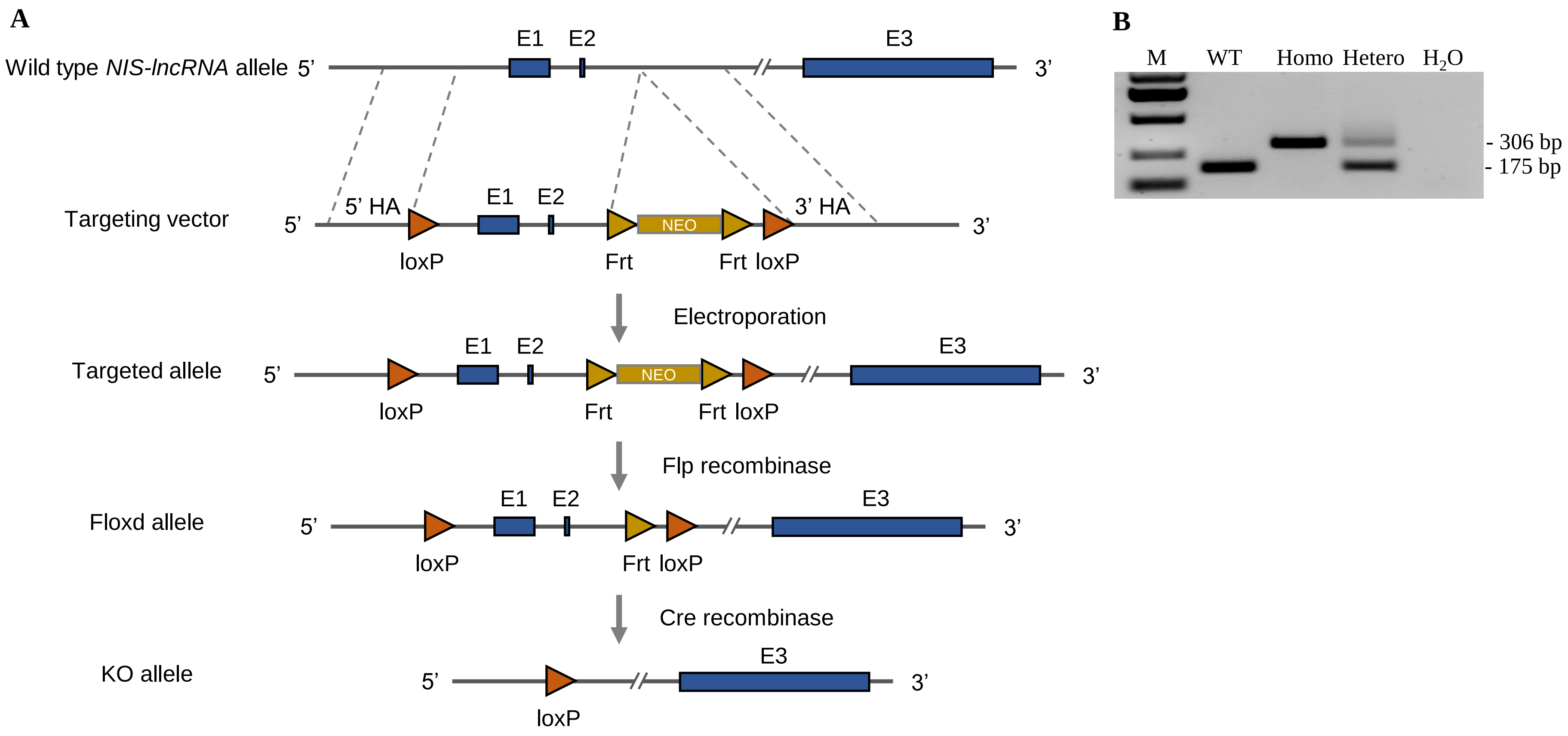

Supplemental Figure 15. Strategy for generation of *NIS-lncRNA*$^{fl/fl}$ mice. (**A**) Constructed vector containing exons 1 and 2 flanked by the loxP sites in *NIS-lncRNA* gene was inserted into *NIS-lncRNA* allele by homologous recombination in C57BL/6 mouse. HA, homologous arm. E1, 2, and 3: *NIS-lncRNA* exons 1, 2, and 3. NEO: neomycin resistance gene. (**B**) Genotyping identification of *NIS-lncRNA*$^{fl/fl}$ mice by PCR. M: DNA ladder marker. Homo: Homozygous. Hetero: Heterozygous. WT: wild type. H_2O: without DNA template.

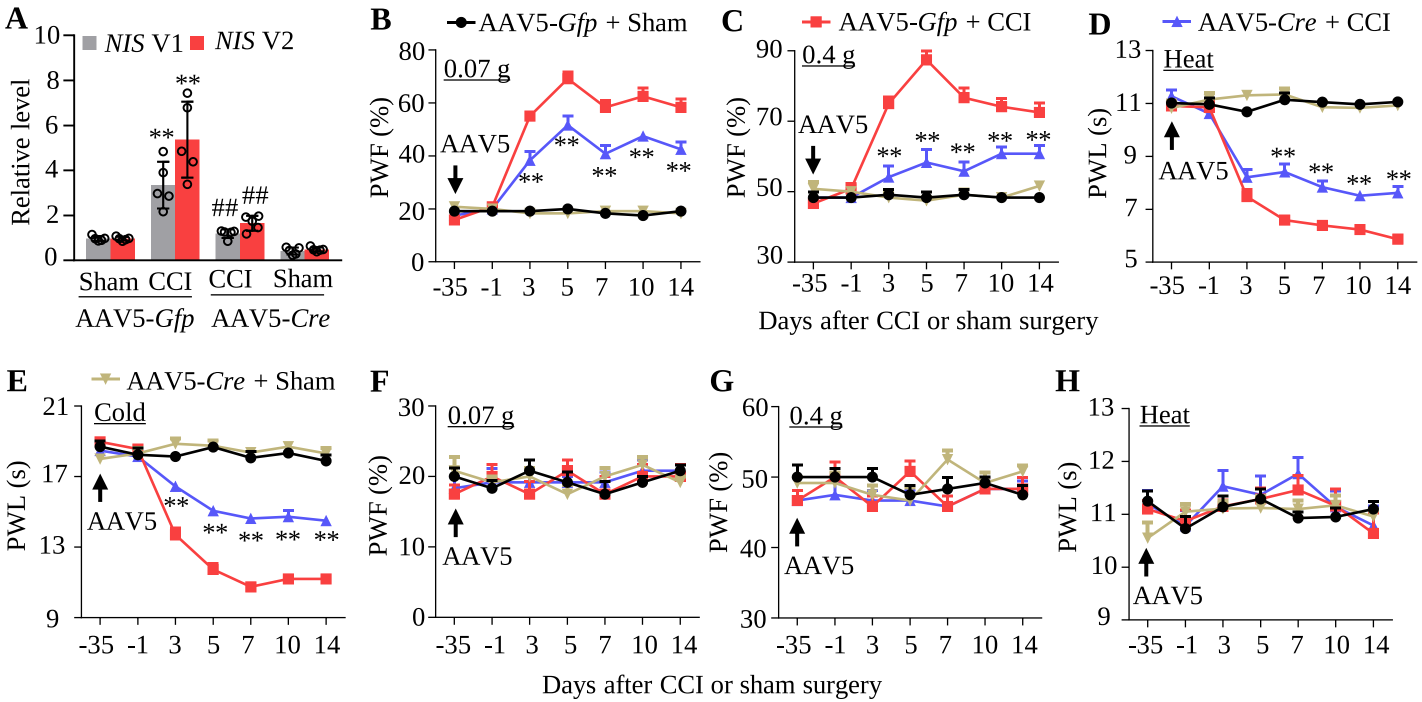

Supplemental Figure 16. DRG knockdown of *NIS-lncRNA* through DRG microinjection of AAV5-*Cre* into ipsilateral L3/4 DRGs of female *NIS-lncRNA*$^{fl/fl}$ mice attenuated the development of CCI-induced nociceptive hypersensitivity. **(A)** Levels of *NIS-lncRNA* V1 *(NIS* V1*)* and *NIS* V2 in the ipsilateral L3/4 DRGs on day 14 after CCI or sham surgery in the *NIS-lncRNA*$^{fl/fl}$ mice with microinjection of AAV5-*Gfp* or AAV5-*Cre* into ipsilateral L3/4 DRGs 35 days before surgery. n = 10 mice/group. $^{**}P < 0.01$ versus the corresponding AAV5-*Gfp* plus sham mice and $^{\#\#}P < 0.01$ versus the corresponding AAV5-*Gfp* plus CCI mice by two-way ANOVA followed by post hoc Tukey test. **(B-H)** Effect of microinjection of AAV5-*Gfp* or AAV5-*Cre* into ipsilateral L3/4 DRG of *NIS-lncRNA*$^{fl/fl}$ mice 35 days before surgery on paw withdrawal responses to 0.07 g (B and F) and 0.4 g (C and G) von Frey filaments and paw withdrawal latencies (PWL) to heat (D and H) and cold (E) stimuli on the ipsilateral (B-E) and contralateral (F-H) sides at the different days as indicated after CCI or sham surgery. n = 12 mice/group. $^{**}P < 0.01$ versus the AAV5-*Gfp* plus CCI mice at the corresponding time points by three-way ANOVA with repeated measures followed by post hoc Tukey test.

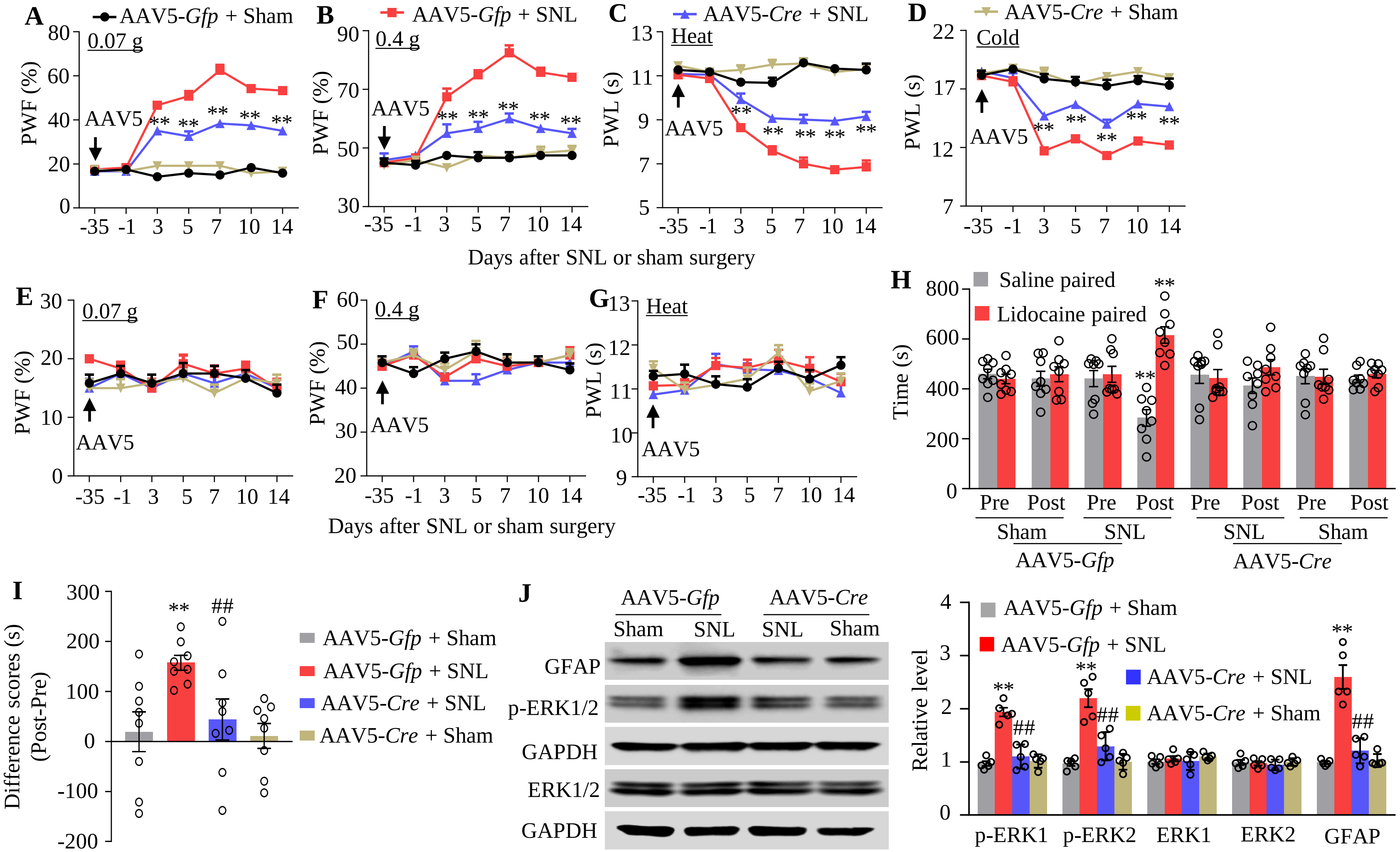
A
AAV5-Gfp + Sham
0.07 g
PWF (%)
AAV5
B
AAV5-Gfp + SNL
0.4 g
C
AAV5-Cre + SNL
Heat
PWL (s)
D
AAV5-Cre + Sham
Cold
Days after SNL or sham surgery
E
F
G
H
Saline paired
Lidocaine paired
Time (s)
Pre
Post
Sham
SNL
AAV5-Gfp
AAV5-Cre
I
Difference scores (s) (Post-Pre)
AAV5-Gfp + Sham
AAV5-Gfp + SNL
AAV5-Cre + SNL
AAV5-Cre + Sham
J
GFAP
p-ERK1/2
GAPDH
ERK1/2
Relative level
p-ERK1
p-ERK2
ERK1
ERK2

Supplemental Figure 17. DRG knockdown of *NIS-lncRNA* through DRG microinjection of AAV5-*Cre* into ipsilateral L4 DRG of male *NIS-lncRNA*$^{fl/fl}$ mice attenuated the development of SNL-induced nociceptive hypersensitivity. **(A-G)** Effect of microinjection of AAV5-*Gfp* or AAV5-*Cre* into ipsilateral L4 DRG of the *NIS-lncRNA*$^{fl/fl}$ mice 35 days before surgery on paw withdrawal responses to 0.07 g (A and E) and 0.4 g (B and F) von Frey filaments and paw withdrawal latencies (PWL) to heat (C and G) and cold (D) stimuli on the ipsilateral (A-D)and contralateral (E-G) sides on the days before or after SNL/sham surgery. n = 12 mice/group. $^{**}P < 0.01$ versus the AAV5-*Gfp* plus SNL mice at the corresponding time points by three-way ANOVA with repeated measures followed by post hoc Tukey test. **(H-I)** Effect of microinjection of AAV5-*Cre* or AAV5-*Gfp* into the ipsilateral L4 DRG of *NIS-lncRNA*$^{fl/fl}$ mice 35 days before surgery on spontaneous ongoing pain as assessed by the CPP paradigm 2 weeks after SNL or sham surgery. Pre: preconditioning. Post: post-conditioning. n = 8 mice/group. $^{**}P < 0.01$ versus the corresponding preconditioning by three-way ANOVA followed by post hoc Tukey test (H) or the AAV5-*Gfp*-treated sham mice by two-way ANOVA followed by post hoc Tukey test (I). ##$P < 0.01$ versus the AAV5-*Gfp*-treated SNL group by two-way ANOVA followed by post hoc Tukey test (I). **(J)** Levels of p-ERK1/2, ERK1/2 and GFAP in the ipsilateral L4 dorsal horn 14 days after SNL or sham surgery from the *NIS-lncRNA*$^{fl/fl}$ mice with microinjection of AAV5-*Cre* or AAV5-*Gfp* into the ipsilateral L4 DRG 35 days post-surgery. n = 5 mice/group. $^{**}P < 0.01$ versus the corresponding AAV5-*Gfp*-treated sham group and #$P < 0.05$, ##$P < 0.01$ versus the corresponding AAV5-*Gfp*-treated SNL group by two-way ANOVA followed by post hoc Tukey test.

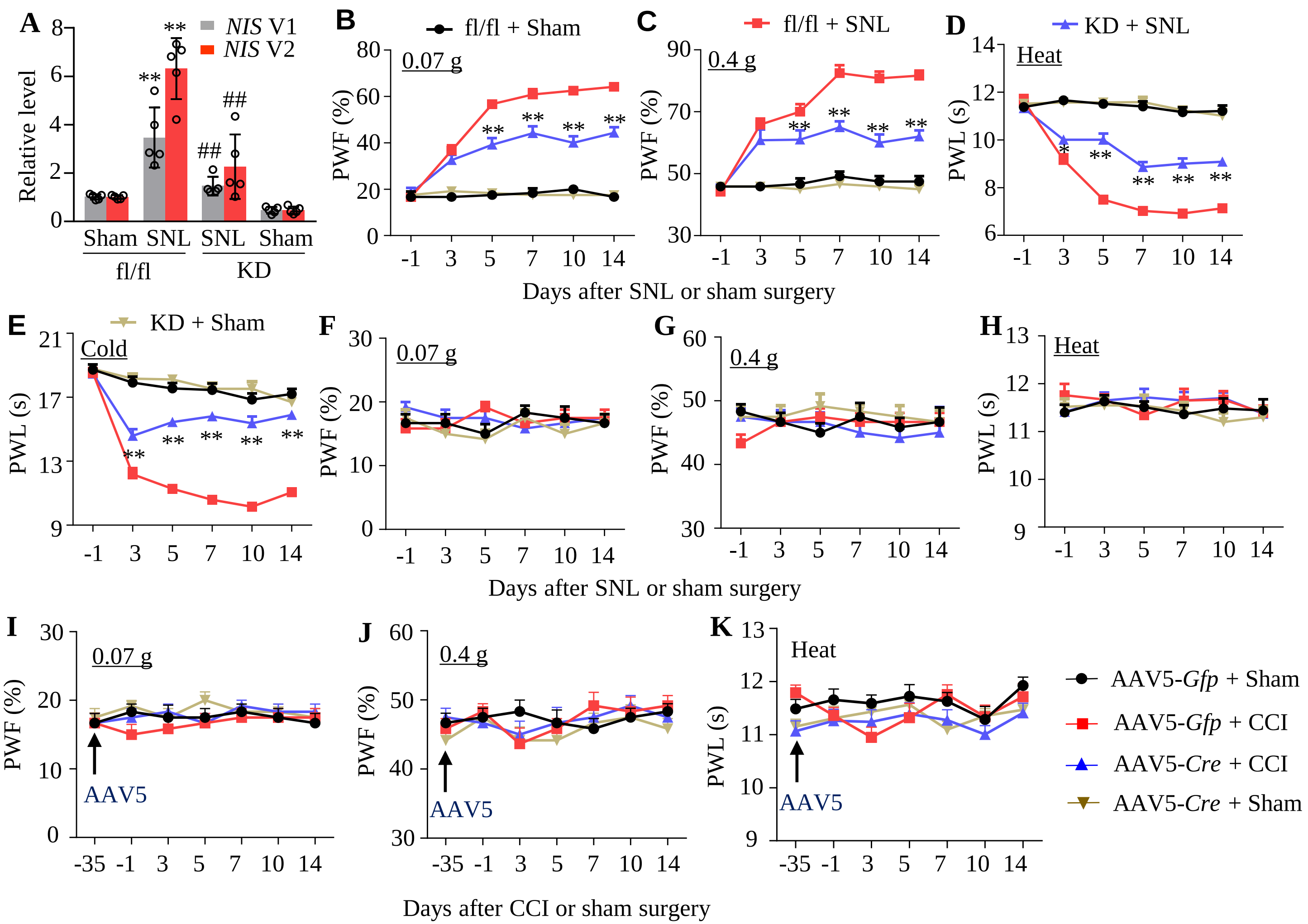

A
B
C
D
E
F
G
H
I
J
K
Relative level
NIS V1
NIS V2
Sham SNL SNL Sham
fl/fl
KD
fl/fl + Sham
fl/fl + SNL
KD + SNL
KD + Sham
0.07 g
0.4 g
Heat
Cold
PWF (%)
PWL (s)
Days after SNL or sham surgery
Days after CCI or sham surgery
AAV5
AAV5-Gfp + Sham
AAV5-Gfp + CCI
AAV5-Cre + CCI
AAV5-Cre + Sham

Supplemental Figure 18. Effect of DRG *NIS-lncRNA* knockdown on basal paw withdrawal responses on both sides of SNL model and on the contralateral side of CCI model in male mice. (**A**) Levels of *NIS* V1 and V2 in the ipsilateral L4 DRG on day 14 after SNL or sham surgery in *NIS-lncRNA*$^{fl/fl}$ mice (fl/fl) or conditional *NIS-lncRNA* knockdown mice (KD). n = 20 mice/group. **P < 0.01 versus the corresponding fl/fl plus sham mice and ##P < 0.01 versus the corresponding fl/fl plus CCI mice by two-way ANOVA followed by *post hoc* Tukey test. (**B-H**) Effect of DRG *NIS-lncRNA* knockdown on the development of SNL-induced nociceptive hypersensitivity. Paw withdrawal frequency (PWF) to 0.07 g (B and F) and 0.4 g (C and G) von Frey filament stimuli and paw withdrawal latency (PWL) to heat (D and H) and cold (E) stimuli from *NIS-lncRNA*$^{fl/fl}$ mice (fl/fl) and the conditional *NIS-lncRNA* knockdown mice (KD) at the different days indicated before or after surgery on the ipsilateral (B-E) and contralateral (F-H) sides. n = 12 mice/group. **P < 0.01 versus the fl/fl plus SNL mice at the corresponding time points by three-way ANOVA with repeated measures followed by *post hoc* Tukey test. **(I-K)** Effect of microinjection of AAV5-*Gfp* or AAV5-*Cre* into ipsilateral L3/4 DRG of the *NIS-lncRNA*$^{fl/fl}$ mice 35 days before surgery on basal paw withdrawal responses to 0.07 g (I) and 0.4 g (J) von Frey filaments and paw withdrawal latencies (PWL) to heat (K) stimulation on the contralateral sides at the different days as indicated after CCI or sham surgery. n = 12 mice/group. Three-way ANOVA with repeated measures followed by post hoc turkey test.

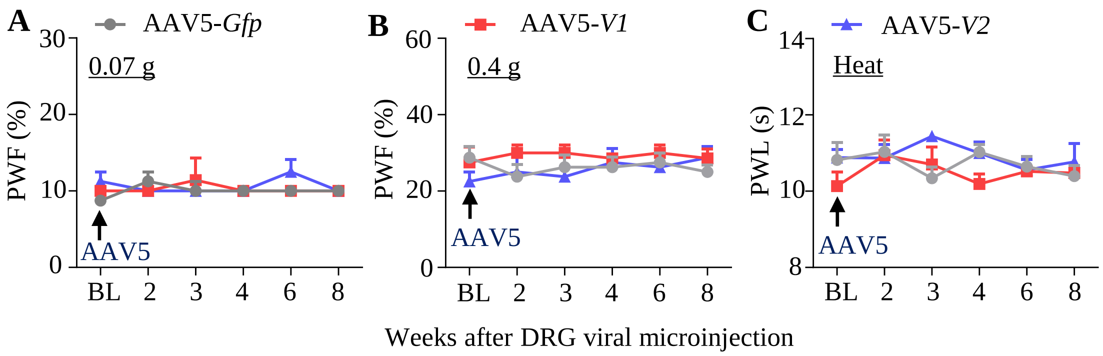

Supplemental Figure 19. Effect of DRG *NIS* V1 or V2 overexpression on the contralateral paw withdrawal responses in male mice. Effect of microinjection of AAV5-*Gfp*, AAV5-*V1* or AAV5-*V2* into ipsilateral L3/4 DRG of naive mice on basal paw withdrawal responses to 0.07 g (A) and 0.4 g (B) von Frey filaments and paw withdrawal latencies (PWL) to heat (C) stimulation on the contralateral sides at the different weeks as indicated after DRG microinjection. n = 8 mice/group. Two-way ANOVA with repeated measures followed by post hoc turkey test.

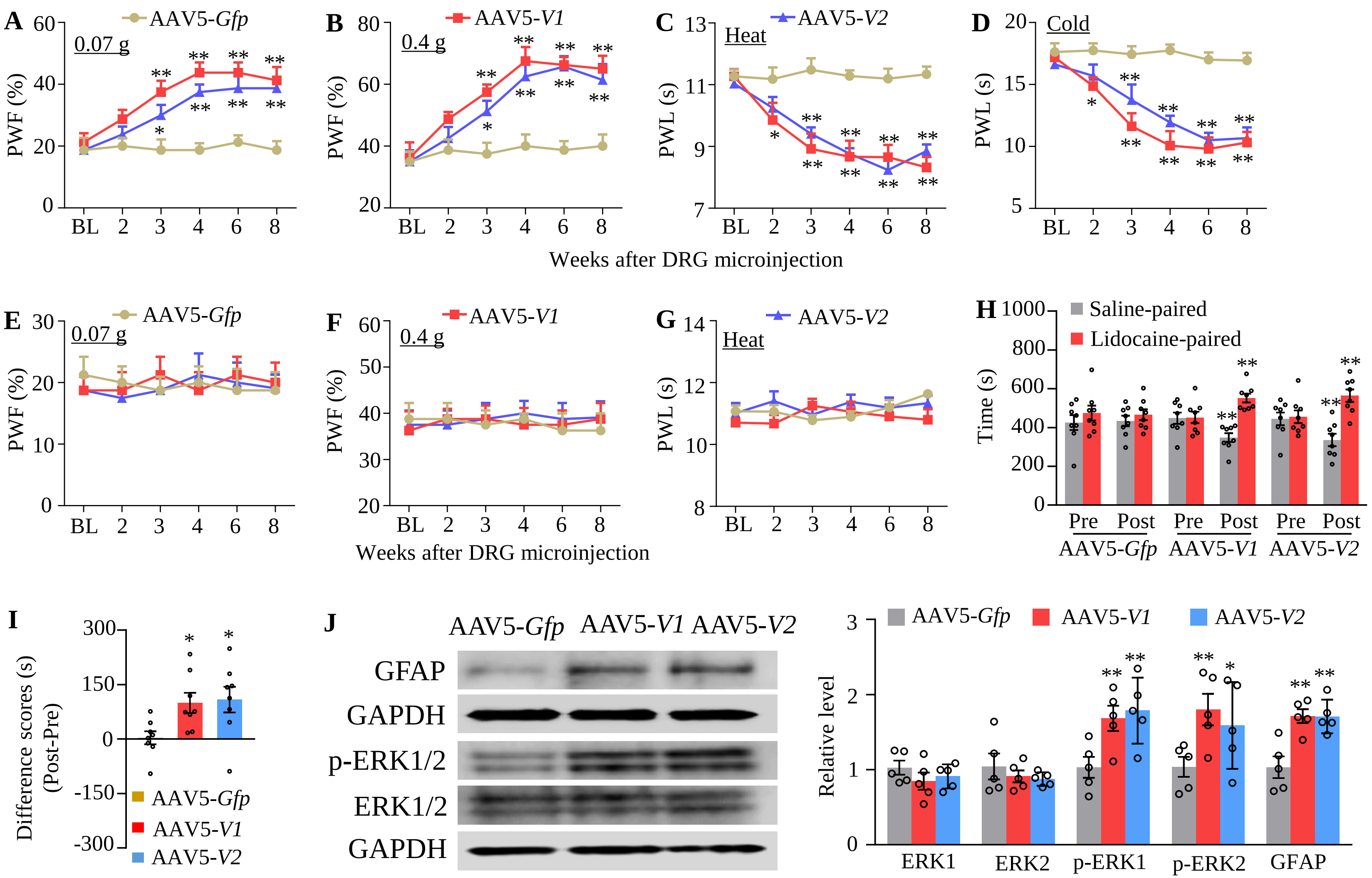

A
B
C
D
AAV5-Gfp
AAV5-V1
AAV5-V2
0.07 g
0.4 g
Heat
Cold
PWF (%)
PWL (s)
Weeks after DRG microinjection
E
F
G
H
Saline-paired
Lidocaine-paired
Time (s)
Pre
Post
I
Difference scores (s) (Post-Pre)
J
GFAP
GAPDH
p-ERK1/2
ERK1/2
Relative level
ERK1
ERK2
p-ERK1
p-ERK2

Supplemental Figure 20. DRG overexpression of NIS-lncRNA produced neuropathic pain-like symptoms in naive female mice. (**A-G**) Effect of microinjection of AAV5-*V1*, AAV5-*V2* or AAV5-*Gfp* into the ipsilateral L3/4 DRGs on paw withdrawal frequency (PWF) to 0.07 g (A and E) and 0.4 g (B and F) von Frey filament stimuli and paw withdrawal latency (PWL) to heat (C and G) and cold (D) stimuli on the ipsilateral (A-D) and contralateral (E-G) sides at time points as shown after viral microinjection. n = 8 mice/group. *$P < 0.05$, **$P < 0.01$ versus the AAV5-*Gfp*-treated mice at the corresponding time points by two-way ANOVA with repeated measures followed by post hoc Tukey test. (**H, I**) Effect of microinjection of AAV5-*V1*, AAV5-*V2* or AAV5-*Gfp* into the ipsilateral L3/4 DRGs on spontaneous ongoing pain as assessed by the CPP paradigm 8 weeks after viral microinjection. Pre: preconditioning. Post: post-conditioning. n = 8 mice/group. H: **$P < 0.01$ versus the corresponding preconditioning by two-way ANOVA with repeated measures followed by post hoc Tukey test. I: **$P < 0.01$ versus the AAV5-Gfp group by one-way ANOVA followed by post hoc Tukey test. (**J**) Levels of p-ERK1/2, ERK1/2 and GFAP in the ipsilateral L3/4 dorsal horn 8 weeks after microinjection of AAV5-*V1*, AAV5-*V2* or AAV5-*Gfp* into the ipsilateral L3/4 DRGs. n = 5 mice/group. *$P < 0.05$, **$P < 0.01$ versus the corresponding AAV5-*Gfp*-treated group by one-way ANOVA followed by post hoc Tukey test.

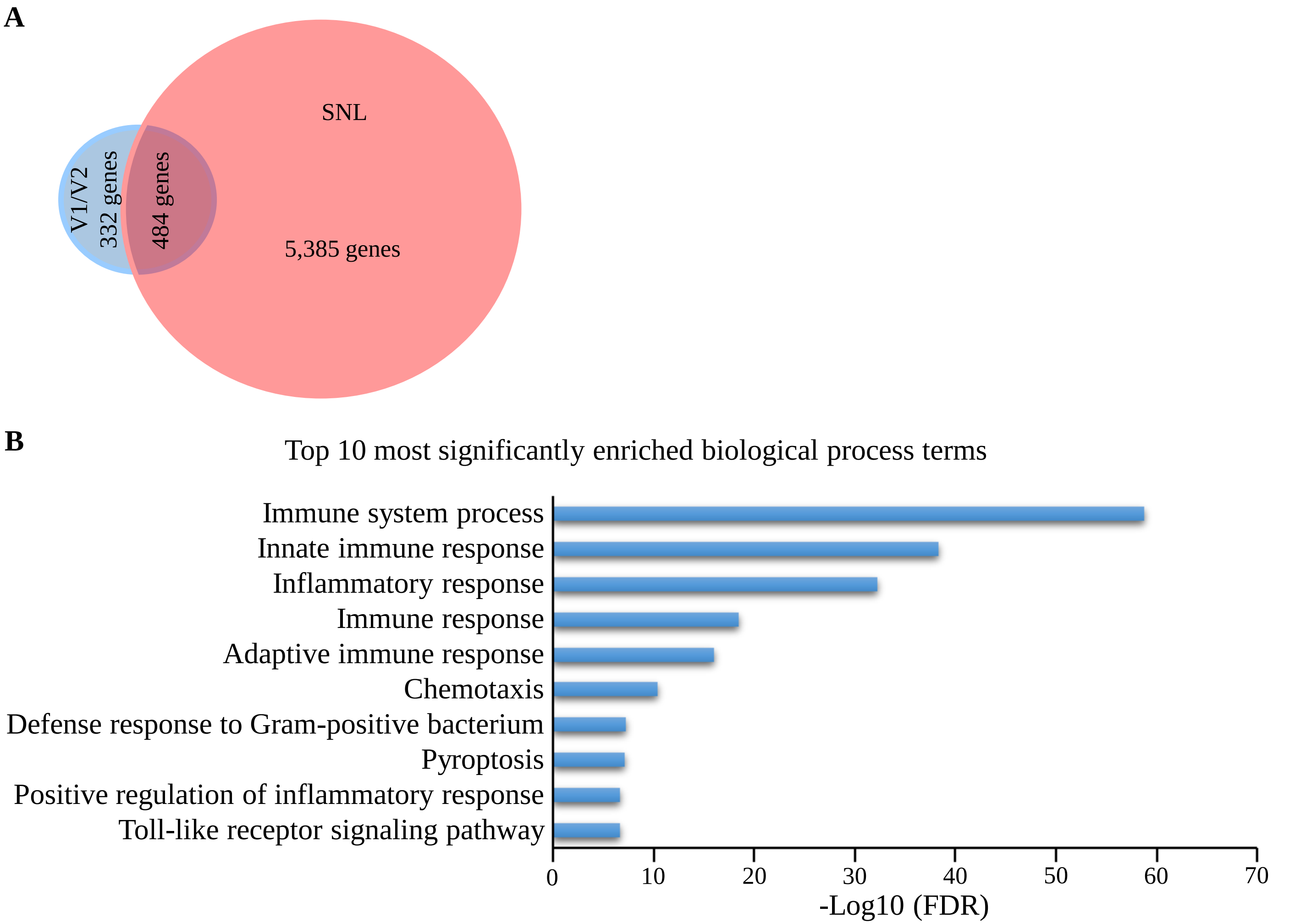

Supplemental Figure 21. Potential downstream targets of *NIS-lncRNA* and analysis of their biological processes. (**A**) Venn diagram of genes from two RNA sequencing data: one from injected DRG 4 weeks post-microinjection of a mixture of AAV5-*V1* (V1) and AAV5 *V2* (V2) and another from injured DRG 7 days after SNL (Ref: 22). About 484 genes overlapped between two databases. (**B**) Analysis of the Gene Ontology database showed top 10 biological process function of these overlapped genes (FDR < 0.05 and $|\log_2$fold-change$|>1$) in DRGs.

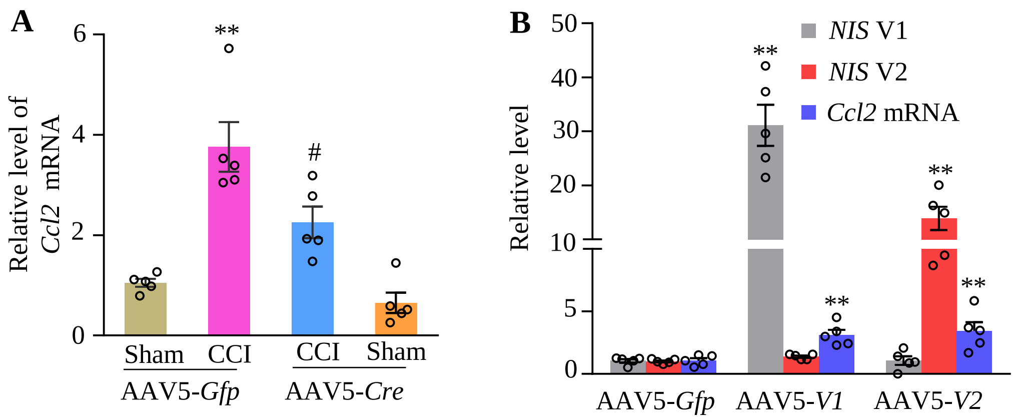

Supplemental Figure 22. Upregulated *NIS-lncRNA* was required for the CCI-induced increase of *Ccl2 mRNA* in injured DRG of female mice. (**A**) Level of *Ccl2* mRNA in the ipsilateral L3/4 DRGs on day 14 after CCI or sham surgery in the *NIS-lncRNA*$^{fl/fl}$ mice with microinjection of AAV5-*Gfp* or AAV5-*Cre* into ipsilateral L3/4 DRGs 35 days before surgery. n = 10 mice/group. **P < 0.01 versus the corresponding AAV5-*Gfp*-microinjected sham mice and #P < 0.05 versus the corresponding AAV5-*Gfp*-microinjected CCI mice by two-way ANOVA followed by post hoc Tukey test. (**B**) Levels of *NIS* V1, *NIS* V2, and *Ccl2* mRNA in the ipsilateral L3/4 DRGs 8 weeks after microinjection of AAV5-*Gfp*, AAV5-*NIS* V1 (AAV5-*V1*) or AAV5-*NIS* V2 (AAV5-*V2*) into unilateral L3/4 DRGs. n = 10 mice/group. **P < 0.01 versus the corresponding AAV5-*Gfp* group by one-way ANOVA followed by post hoc Tukey test.

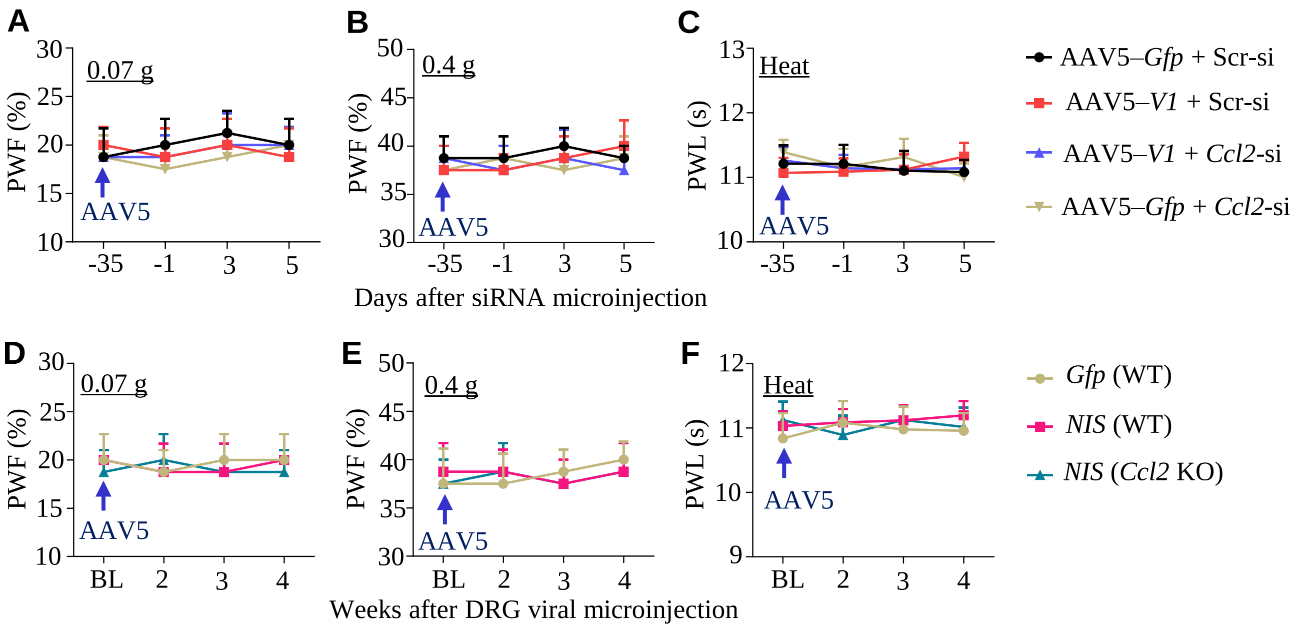

Supplemental Figure 23. Effect of genetic knockdown/knockout of DRG CCL2 on paw withdrawal responses on the contralateral side in male mice. (**A-C**) Paw withdrawal frequency (PWF) to 0.07 g (A) and 0.4 g (B) von Frey filament stimuli and paw withdrawal latency (PWL) to heat (C) stimulation on the contralateral side on the days as shown before or after microinjection of *Ccl2* siRNA (Ccl2-si) or scrambled siRNA (Scr-si) into unilateral L3/4 DRGs in the mice with microinjection of AAV5-*V1* or AAV5-*Gfp* for 35 days. n = 8 mice/group. Three-way ANOVA with repeated measures followed by post hoc Tukey test. (**D-F**) Paw withdrawal frequency (PWF) to 0.07 g (D) and 0.4 g (E) von Frey filament stimuli and paw withdrawal latency (PWL) to heat (F) stimulation on the weeks as shown after microinjection of AAV5-*Gfp (Gfp)*or a mixture of AAV5-*Gfp* and AAV5-*V1* (*NIS*) into unilateral L3/4 DRGs on the contralateral side of wild type (WT) or *Ccl2* knockout (KO) mice. n = 8 mice/group. Three-way ANOVA with repeated measures followed by post hoc Tukey test.

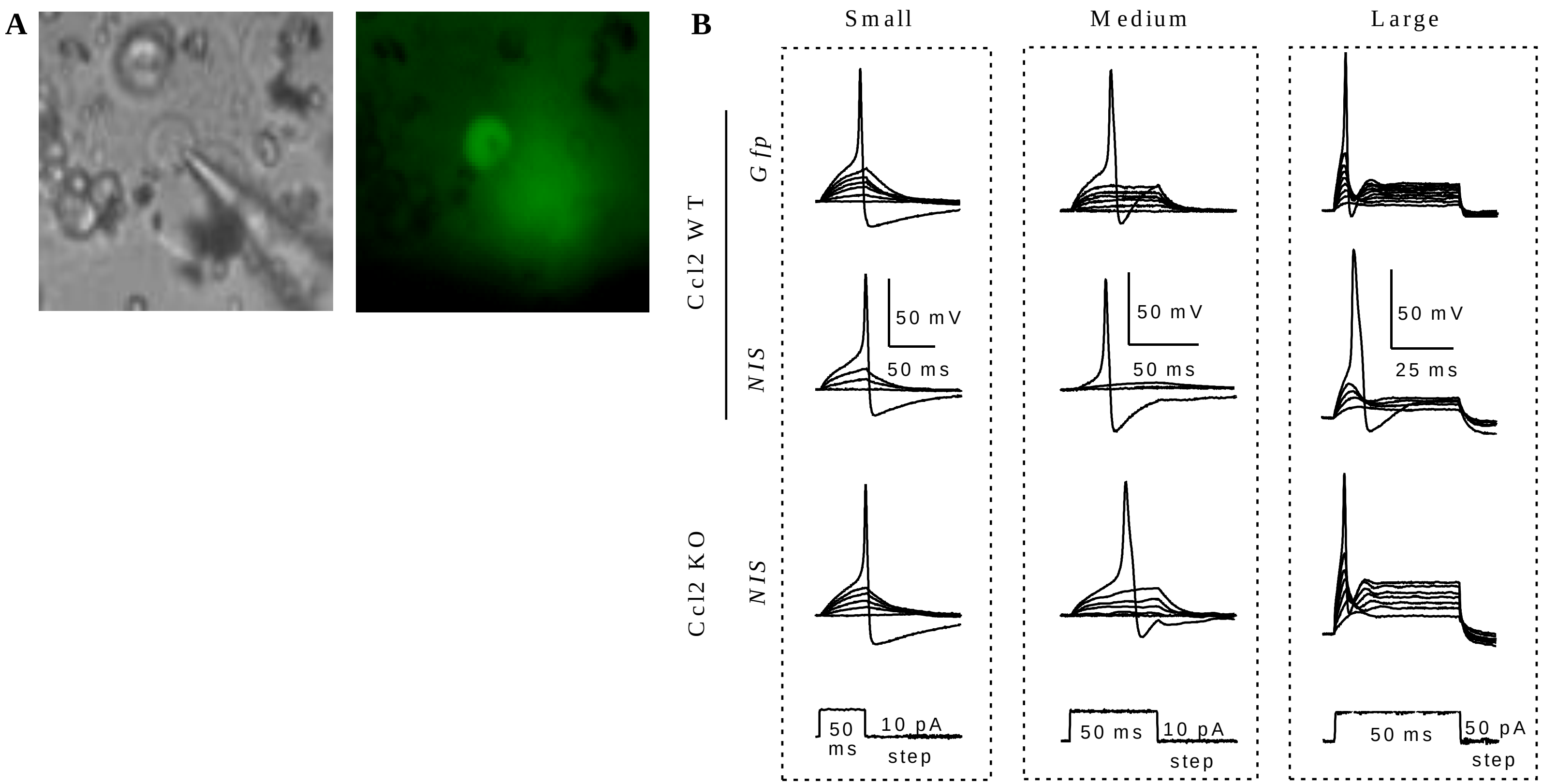

Supplemental Figure 24. (A) Representative images showing that only green color-labeled neuron was recorded from acute disassociated neurons of the ipsilateral L3/4 DRGs 4 weeks after microinjection of AAV5-*Gfp* or a mixture of *AAV5-Gfp* and AAV5-*V1* into unilateral L3/4 DRGs of wild type (WT) or *Ccl2* knockout (KO) mice. (**B**) Representative traces of rheobase in small, medium and large DRG neurons from wild type (WT) or *Ccl2* knock out (KO) mice with microinjection of AAV5-*Gfp (Gfp)* or a mixture of AAV5-*Gfp* and AAV5-*V1* (*NIS*) into unilateral L3/4 DRGs for 4 weeks.

A

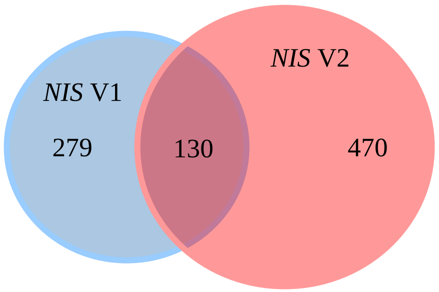

B

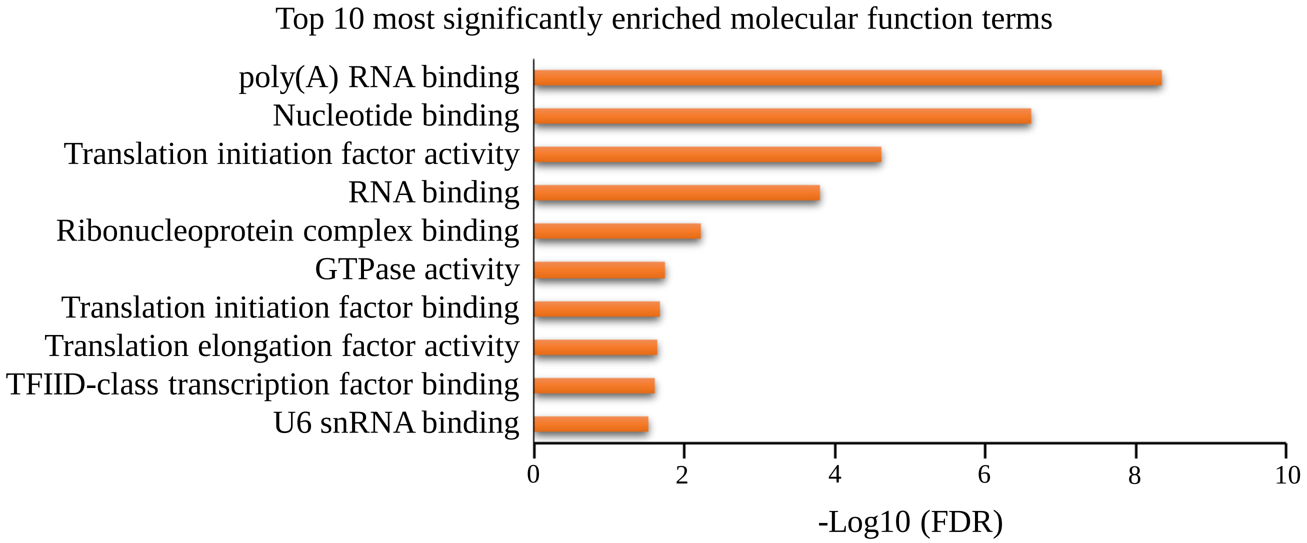

Supplemental Figure 25. Potential *NIS-lncRNA*-binding proteins and analysis of their molecular function. (**A**) Venn diagram of the proteins from the cultured DRG neurons incubated with specific *NIS* V1 and V2 sense RNA probes, respectively. *NIS* V1 and *NIS* V2 interacted with 409 and 600 proteins, respectively. About 130 proteins were overlapped between two mass spectrometry databases. (**B**) Analysis of the Gene Ontology database showed top 10 enriched molecular function of these overlapped proteins in DRGs.

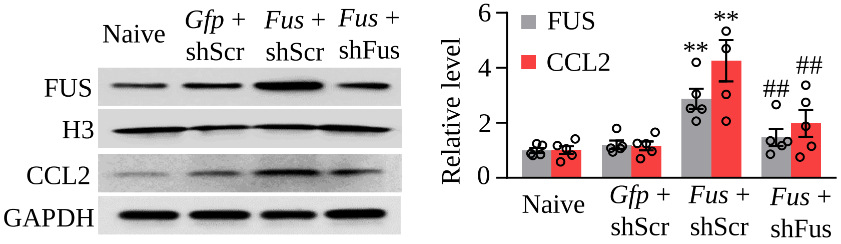

Supplemental Figure 26. **DRG overexpression of FUS increased CCL2 expression in DRG neurons**. Levels of FUS and CCL2 proteins in the cultured DRG neuron with transduction as indicated. Naive: no transduction. *Fus*: AAV5 expressing *Fus* mRNA. *Gfp*: AAV5 expressing *Gfp* mRNA. shFus: AAV5 expressing *Fus* shRNA. shScr: AAV5 expressing scrambled shRNA. n = 5 repeats/treatment. ***P* < 0.01 versus the corresponding naive group and ##*P* < 0.01 versus the corresponding *Fus* plus shScr group by one-way ANOVA with *post hoc* Tukey test.

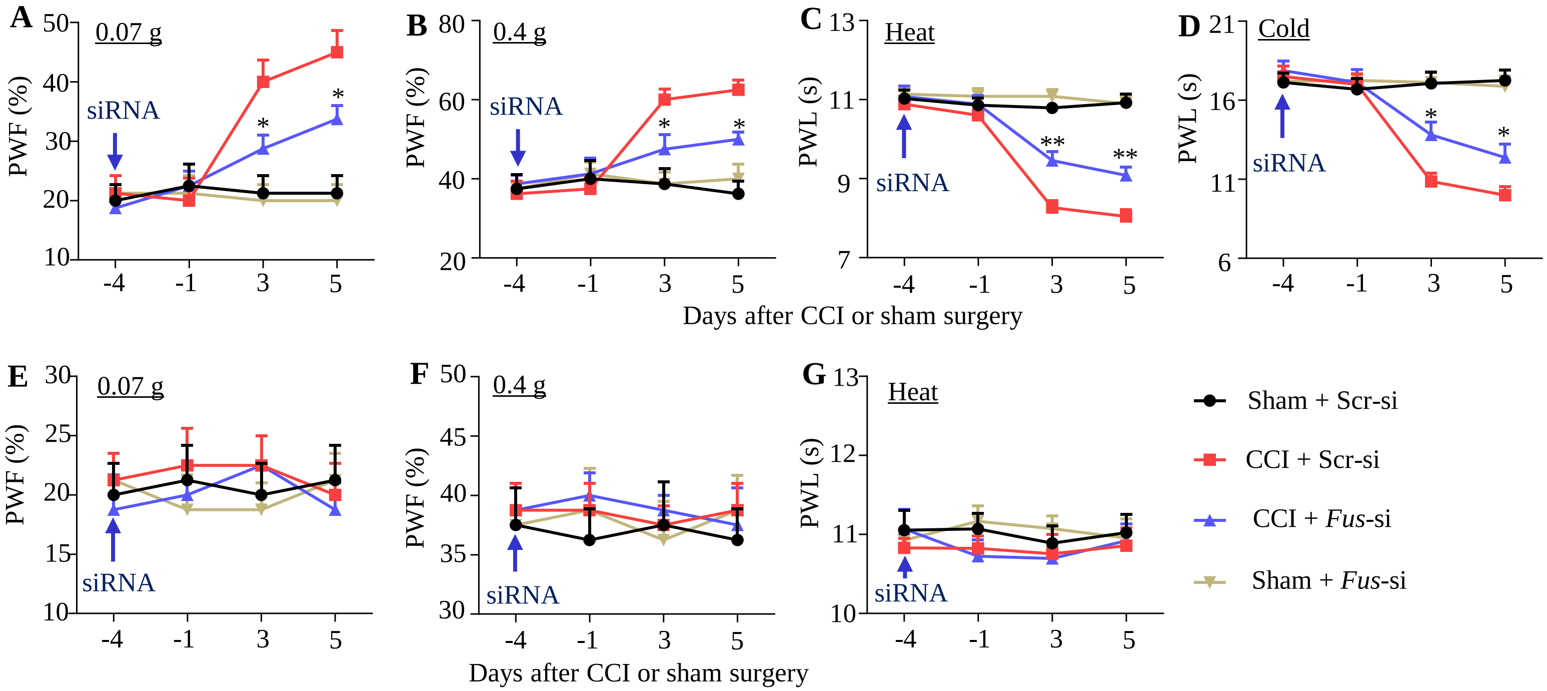

Supplemental Figure 27. Blocking the CCI-induced increase of DRG FUS attenuated the development of the CCI-induced nociceptive hypersensitivities in male mice. **(A-G)** Effect of microinjection of *Fus* siRNA (*Fus*-si) or control scrambled siRNA (Scr-si) into ipsilateral L3/4 DRGs 4 days before surgery on paw withdrawal responses to 0.07 g (A, E) and 0.4 g (B, F) von Frey filaments and paw withdrawal latencies (PWL) to heat (C, G) and cold (D) stimuli on the ipsilateral (A-D) and contralateral (E-G) sides at time points as shown after CCI or sham surgery. n = 8 mice/group. **P* < 0.05， ***P* < 0.01 versus the CCI plus Scr-si group at the corresponding time points by three-way ANOVA with repeated measures followed by post hoc Tukey test.

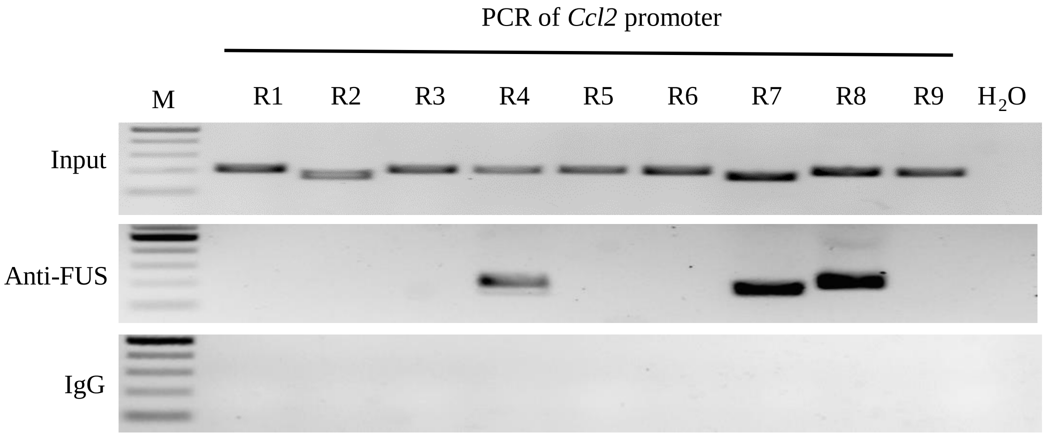

Supplemental Figure 28. Potential binding of FUS to the regions of *Ccl2* promoter. Three regions (R4, -196/+5; R7, -589/-399; R8, -748/-542), but not other regions (R1, +321/+546; R2, +142/+334; R3, -18/+186; R5, -345/-138; R6, -443/-237; R9, -863/-661), from the *Ccl2* gene promoter were immunoprecipitated by mouse anti-FUS (not by mouse purified IgG) in mouse lumbar DRG. Input: total purified fragments. M: DNA ladder marker. n = 3 biological repeats (mice). H_2O, no cDNA, is a negative control.

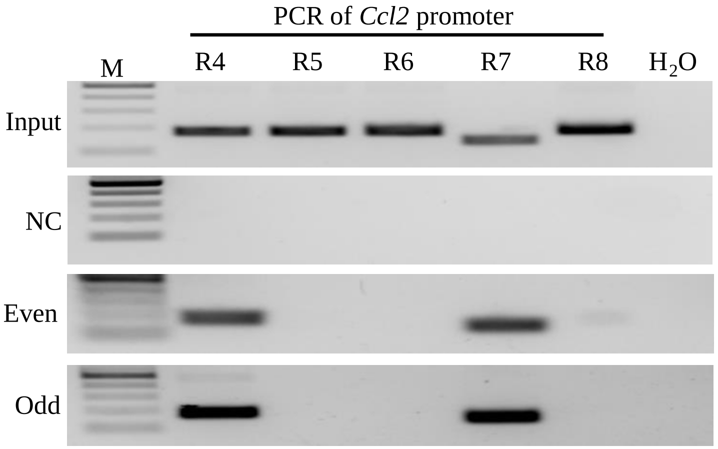

Supplemental Figure 29. Potential binding of *NIS-lncRNA* to the regions of *Ccl2* promoter. A total of 14 different biotinylated antisense DNA probes that were complementary to the sequence of full-length of *NIS-lncRNA* were designed and numbered. Seven even-numbered probers (Even) and 7 odd-numbered probers (Odd) were pooled together, respectively, for hybridization of cell lysates. R4: -196/+5. R5: -345/-138. R6: -443/-237. R7: -589/-399. R8: -748/-542. Input: extracted DNA. NC: negative control prober. M: DNA ladder marker. n = 3 biological repeats.

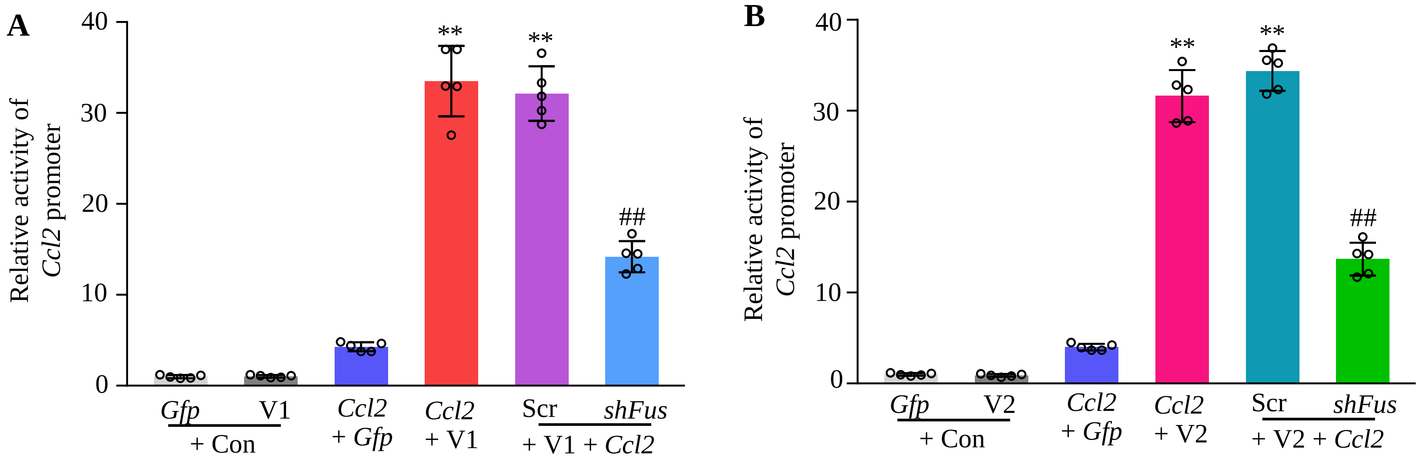

Supplemental Figure 30. *NIS* V1- or *NIS* V2-induced increase in promoter activity of *Ccl2* gene is FUS-dependent. (**A**) Effect of *NIS* V1 on *Ccl2* gene promoter (R7 and R8) activities in *in vitro* CAD cells transfected as shown. Con: control vector (pGL3-Basic). Ccl2: pGL3 reporter vector with *Ccl2* promoter. V1: vector expressing full-length *NIS* V1 (V1). *Gfp*: vector expressing *Gfp* mRNA. *shFus*: vector expressing *Fus* shRNA. Scr: vector expressing control scrambled shRNA. n = 5 biological repeats/treatment. ***P* < 0.01 versus the Con plus *Gfp*-treated group by one-way ANOVA with post hoc Tukey test. ## *P* < 0.01 versus the V1 plus Scr-treated group by one-way ANOVA with post hoc Tukey test. (**B**) Effect of *NIS* V2 on *Ccl2* gene promoter (R7 and R8) activities in *in vitro* CAD cells transfected as shown. Con: control vector (pGL3-Basic). *Ccl2*: pGL3 reporter vector with *Ccl2* promoter. V2: vector expressing full-length *NIS* V2 (V2). *Gfp*: vector expressing *Gfp*. *shFus*: vector expressing *Fus* shRNA. Scr: vector expressing control scrambled shRNA. n = 5 biological repeats/treatment. ** *P* < 0.01 versus the con plus *Gfp*-treated group by one-way ANOVA with post hoc Tukey test. ## *P* < 0.01 versus the V2 plus Scr-treated group by one-way ANOVA with post hoc Tukey test.

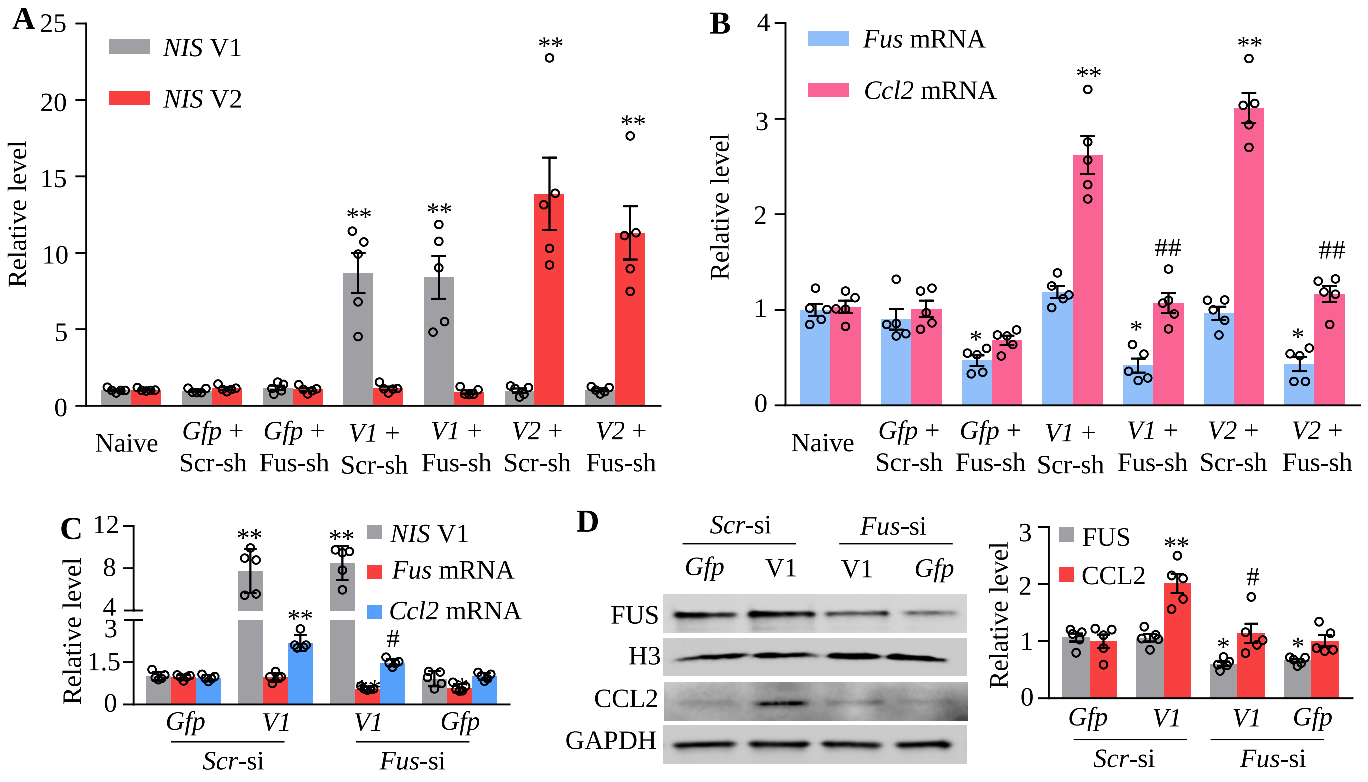

Supplemental Figure 31. *NIS-lncRNA*-induced increase of *Ccl2 mRNA* is dependent on *Fus* in DRG neurons. (**A** and **B**) Levels of *NIS* V1 (A), *NIS* V2 (A), *Fus* mRNA (B) and *Ccl2* mRNA (B) in primary cultured DRG neurons transduced with AAV5 as indicated. *Gfp*: AAV5 expressing *Gfp* mRNA. *V1*: AAV5 expressing full-length *NIS-IncRNA* variant 1. *V2:* AAV5 expressing full-length *NIS-IncRNA* variant 2. Fus-sh: AAV5 expressing *Fus* short hairpin RNA. Scr-sh: AAV5 expressing control scrambled short hairpin RNA. n = 5 biological repeats. ***P* < 0.01 versus the corresponding naive group by one-way ANOVA with post hoc Tukey test. ## *P* < 0.01 versus the corresponding *V1* or *V2* plus Scr-sh group by one-way ANOVA with post hoc Tukey test. (**C** and **D**) Levels of *NIS* V1 (C), *Fus* mRNA (C), *Ccl2* mRNA (C), FUS protein (D) and CCL2 protein (D) in the ipsilateral L3/4 DRGs 5 days after DRG microinjection of *Fus* siRNA (*Fus*-si) or control scrambled siRNA (*Scr*-si) in mice with DRG microinjection of AAV5-*NIS* V1 *(V1)* or AAV5-*Gfp (Gfp)* 35 days before siRNA microinjection. n = 10 mice/group. **P* < 0.05, ***P* < 0.01 versus the corresponding *Scr*-si-treated *Gfp* group and #*P* < 0.05 versus the corresponding *Scr*-si-treated *V1* group by two-way ANOVA with *post hoc* Tukey test.

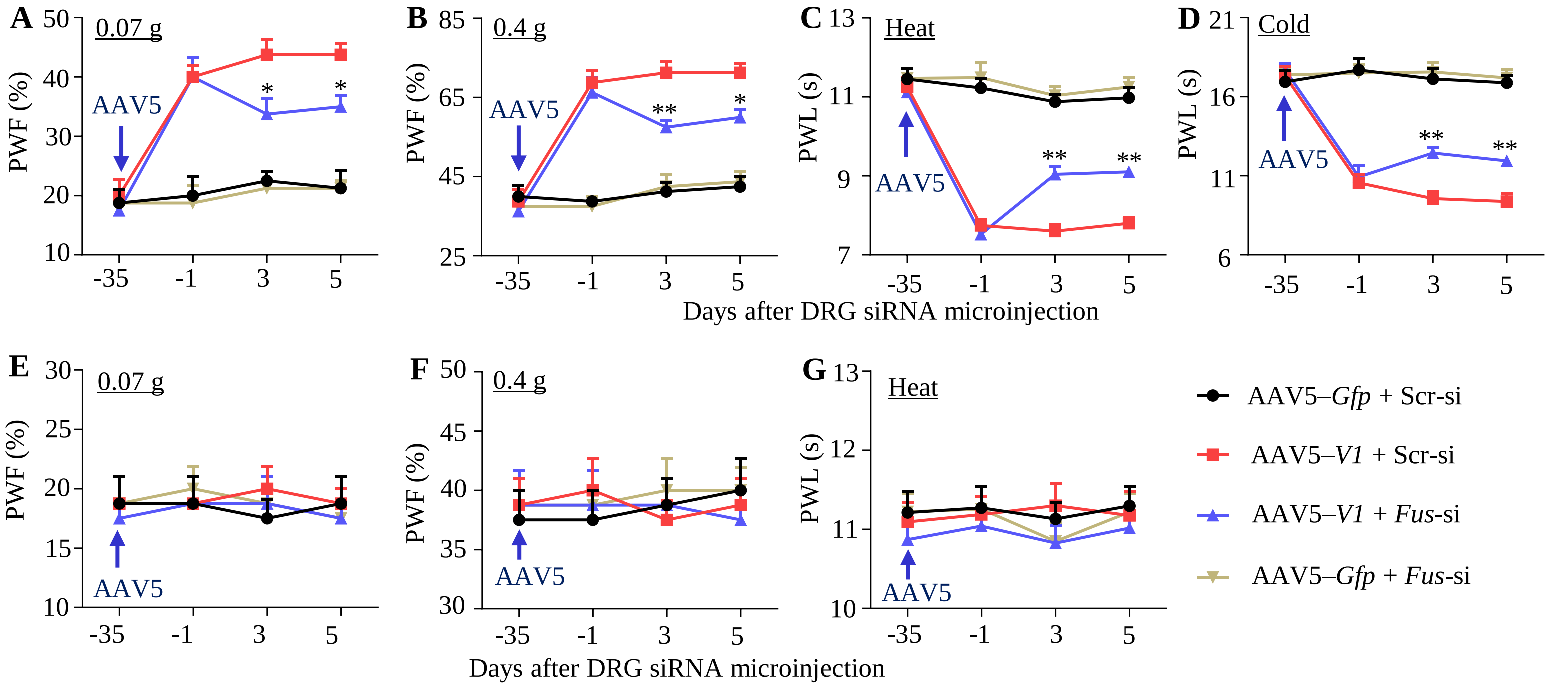

Supplemental Figure 32. DRG FUS knockdown attenuated nociceptive hypersensitivities caused by DRG overexpression of *DIS-lncRNA* variant V1 in naive male mice. **(A-G)** Effect of microinjection of *Fus* siRNA (*Fus*-si) or control scrambled siRNA (Scr-si) into unilateral L3/4 DRGs on paw withdrawal responses to 0.07 g (A, E) and 0.4 g (B, F) von Frey filaments and paw withdrawal latencies (PWL) to heat (C, G) and cold (D) stimuli on the ipsilateral (A-D) and contralateral (E-G) sides at time points as shown in the mice with microinjection of AAV5 expressing full-length *NIS* V1 *(*AAV5-*V1)* or AAV5-*Gfp* 35 days before siRNA microinjection. n = 8 mice/group. **P* < 0.05, ***P* < 0.01 versus the AAV5-*V1* plus Scr-si group at the corresponding time points by two-way ANOVA with repeated measures followed by post hoc Tukey test.

Supplemental Table S1. Locomotor functions

Treated groups	Placing	Grasping	Righting
Sham + Vehicle	5(0)	5(0)	5(0)
CCI + Vehicle	5(0)	5(0)	5(0)
CCI + Scrambled siRNA	5(0)	5(0)	5(0)
CCI + NIS-siRNA	5(0)	5(0)	5(0)
Sham + NIS-siRNA	5(0)	5(0)	5(0)
CCI + V1-siRNA	5(0)	5(0)	5(0)
Sham + V1-siRNA	5(0)	5(0)	5(0)
CCI + V2-siRNA	5(0)	5(0)	5(0)
Sham + V2-siRNA	5(0)	5(0)	5(0)
AAV5-*Gfp* + Sham	5(0)	5(0)	5(0)
AAV5-*Gfp* + CCI	5(0)	5(0)	5(0)
AAV5-*Cre* + Sham	5(0)	5(0)	5(0)
AAV5-*Cre* + CCI	5(0)	5(0)	5(0)
AAV5-*Gfp* + SNL	5(0)	5(0)	5(0)
AAV5-*Cre* + SNL	5(0)	5(0)	5(0)
$NIS^{fl/fl}$ + Sham	5(0)	5(0)	5(0)
$NIS^{fl/fl}$ + SNL	5(0)	5(0)	5(0)
Adv^{cre} $NIS^{fl/fl}$ + Sham	5(0)	5(0)	5(0)
Adv^{cre} $NIS^{fl/fl}$ + SNL	5(0)	5(0)	5(0)
AAV5-*Gfp*	5(0)	5(0)	5(0)
AAV5-V1	5(0)	5(0)	5(0)
AAV5-V2	5(0)	5(0)	5(0)
Scrambled siRNA + Sham	5(0)	5(0)	5(0)
Scrambled siRNA + SNL	5(0)	5(0)	5(0)
Elf1-siRNA + SNL	5(0)	5(0)	5(0)
Elf1-siRNA + Sham	5(0)	5(0)	5(0)
AAV5-*Gfp* + Scrambled siRNA	5(0)	5(0)	5(0)
AAV5-*Elf1* + Scrambled siRNA	5(0)	5(0)	5(0)
AAV5-*Elf1* + NIS-siRNA	5(0)	5(0)	5(0)
AAV5-*Gfp* + NIS-siRNA	5(0)	5(0)	5(0)
AAV5-*V1* + Scrambled siRNA	5(0)	5(0)	5(0)
AAV5-*V1* + Ccl2-siRNA	5(0)	5(0)	5(0)
AAV5-*Gfp* + Ccl2-siRNA	5(0)	5(0)	5(0)
AAV5-*V1* + Vehicle	5(0)	5(0)	5(0)
AAV5-*V1* + CCR2-RA	5(0)	5(0)	5(0)
AAV5-V1 (*Ccl2* WT)	5(0)	5(0)	5(0)
AAV5-V1 (*Ccl2* KO)	5(0)	5(0)	5(0)
CCI + FUS-siRNA	5(0)	5(0)	5(0)
Sham + FUS-siRNA	5(0)	5(0)	5(0)
AAV5-*V1* + Fus-siRNA	5(0)	5(0)	5(0)
AAV5-*Gfp* + Fus-siRNA	5(0)	5(0)	5(0)

All values are Mean (SEM). n = 8-12 mice/group. 5 trials.

Supplemental Table 2. Effect of *NIS* V1 on membrane input resistance and other action potential parameters in the DRG neurons from WT and *Ccl2* KO mice

Large cell

	Gfp in WT	*NIS* in WT	*NIS* in KO	F/p value
**n**	20 cells, 14 mice	22 cells, 18 mice	22 cells, 18 mice	
R_{in}, MΩ	163.2 ± 18.52	174.36 ± 17.86	176.77 ± 14.20	0.17/0.84
APA, mV	90.93 ± 2.16	94.91 ± 2.25	89.66 ± 2.06	1.60/0.21
APT, mV	-19.04 ± 0.47	-21.69 ± 0.69	-21.09 ± 0.79	2.54/0.08
APO, mV	38.4 ± 1.67	42.08 ± 2.29	37.87 ± 1.63	1.39/0.26
AHPA, mV	-11.98 ± 2.25	-13.24 ± 4.23	-12.31 ± 5.31	0.60/0.55

Medium cell

	Gfp in WT	*NIS* in WT	*NIS* in KO	F/p value
**n**	23 cells, 14 mice	24 cells, 18 mice	24 cells, 18 mice	
R_{in}, MΩ	387.07 ± 34.84	403.08 ± 39.75	408.29 ± 28.70	0.10/0.91
APA, mV	90.35 ± 2.26	88.52 ± 2.00	89.08 ± 2.55	0.16/0.85
APT, mV	-21.92 ± 0.99	-19.71 ± 0.75	-21.29 ± 0.84	1.57/0.22
APO, mV	40.52 ± 1.69	44.21 ± 1.14	40.92 ± 1.52	1.77/0.18
AHPA, mV	-15.82 ± 1.28	-13.95 ± 1.06	-14.58 ± 1.20	0.64/0.53

Small cell

	Gfp in WT	*NIS* in WT	*NIS* in KO	F/p value
**n**	22 cells, 14 mice	24 cells, 18 mice	24 cells, 18 mice	
R_{in}, MΩ	402.54 ± 37.78	412.23 ± 37.17	425.13 ± 37.10	0.09/0.91
APA, mV	89.18 ± 1.89	87.78 ± 1.69	88.79 ± 1.63	0.16/0.85
APT, mV	-19.27 ± 0.75	-18.30 ± 0.72	-19.26 ± 0.86	0.43/0.63
APO, mV	37.27 ± 1.26	44.39 ± 1.28	39.65 ± 1.29	2.91/0.06
AHPA, mV	-16.27 ± 0.81	-18.06 ± 1.10	-14.43 ± 0.75	2.28/0.11

Values are mean ± SEM. R_{in}: membrane input resistance. APA: action potential amplitude. APT: action potential threshold. APO: action potential overshoot. AHPA: afterhyperpolarization amplitude.

Supplemental Table 3. All primers, probes and siRNAs used

Names	Sequences
RT-PCR	
Mouse-NIS F	5′- GGGTGAGGACATCCAAGAGA-3′
Mouse-NIS R	5′- CGTTCTAGCCTCAGCTGTCC-3′
Rat-NIS F	5′- GGAAAGATGATCCGAGCAAA-3′
Rat-NIS R	5′- CTCCCCTCTCCCAAGTCTTC-3′
Rat-GAPDH F	5′- TCGGTGTGAACGGATTTGGC-3′
Rat-GAPDH R	5′- CCTTCAGGTGAGCCCCAGC-3′
Human-NIS F	5′- CAGTCAGTGGGCAGAAGACA-3′
Human-NIS R	5′- GGCTGATGATTCCCAACATT-3′
Human-GAPDH F	5′- TCACCATCTTCCAGGAGCG-3′
Human-GAPDH R	5′- CTGCTTCACCACCTTCTTGA-3′
mNIS V1 F	5′- GGAGAGAGACAAGGGGAAGG-3′
mNIS V1 R	5′- TGTCAGTGACGTAGCCAAGC-3′
mNIS V2 F	5′- AGCTTGGGAAGCAGATTTGA-3′
mNIS V2R	5′- TGTCTTCACACTGGCAGGAG-3′
Total-mNIS F	5′- GGGTGAGGACATCCAAGAGA-3′
Total-mNIS R	5′- CGTTCTAGCCTCAGCTGTCC-3′
mElf1 F	5′- CAGGAGGAAGCAGCAAATTC-3′
mElf1 R	5′- GAGACCCAGCACCTGATGAT-3′
mCcl2 F	5′- AGCACCAGCCAACTCTCACT-3′
mCcl2 R	5′- CGTTAACTGCATCTGGCTGA-3′
mFus F	5′- GGCTACTCCCAACAGAGCAG-3′
mFus R	5′- ATATCCCTGGGGAGCTGACT-3′
mTuba1a F	5′- GTGCATCTCCATCCATGTTG-3′
mTuba1a R	5′- GTGGGTTCCAGGTCTACGAA-3′
mGAPDH F	5′- TCGGTGTGAACGGATTTGGC-3′
mGAPDH R	5′- TCCCATTCTCGGCCTTGACT-3′
Northern blot	
NIS V1 F	5′- AAGGGAACGCAGTGAGTGAG-3′
NIS-V1 R	5′- TAATACGACTCACTATAGGGAGGGCTGGAAACCTGAAGTC-3′
NIS-V2 F	5′- TCACCAAGACGGGCAAATC-3′
NIS-V2 R	5′- TAATACGACTCACTATAGGGTAAGAAGTGGTCAAGATTT-3′
In vitro protein translation	
T7-NIS V1 F	5′- TAATACGACTCACTATAGGGACAAAGGGAACGCAGTGAG-3′
T7-NIS V1 R	5′- TATGCATGCTGTTTATTTTAG-3′
T7-NIS V2 F	5′- TAATACGACTCACTATAGGGTCACCAAGACGGGCAAATC-3′
T7-NIS V2 R	5′- TAAGAAGTGGTCAAGATTT-3′
T7-Creb1 F	5′- TAATACGACTCACTATAGGGAGCCACCATGACCATGGAATCTGGA-3′
T7-Creb1 R	5′- TTAATCTGATTTGTGGCAGTAAAG-3′
RACE primers	
Oligo d(T)-anchor	5′- GACCACGCGTATCGATGTCGACTTTTTTTTTTTTTTTTV-3′
PCR anchor primer	5′- GACCACGCGTATCGATGTCGAC-3′
Mouse:	
NIS-V1 RT for 5'RACE	5′- TGTCAGTGACGTAGCCAAGC-3′
NIS-V2 RT for 5'RACE	5′- AGCCAGAGGCTGTAGGTGAA-3′
Nested SP2 primer for 5'RACE	5′- CGTTCTAGCCTCAGCTGTCC -3′
Nested SP3 primer for 5'RACE	5′- GCTCATCTTTCCTGCACGTC -3′
NIS-V1 SP5 primer for 3'RACE	5′- ATGACGACGTGCAGGAAAGAT -3′
NIS-V2 SP5 primer for 3'RACE	5′- TTCTTTTCGCTGGATCTCCTC -3′
Rat:	
RT for 5'RACE	5′- CTCCCCTCTCCCAAGTCTTC-3′
Nested SP2 primer for 5'RACE	5′- CCTTCTTGGGCTCACTCCTCTC-3′
SP5 primer for 3'RACE	5′- GAGAGGAGTGAGCCCAAGAAGG-3′
Human:	
RT for 5'RACE	5′- GCTGATGATTCCCAACATTC-3 ′
Nested SP2 primer for 5'RACE	5′- CTCACTTTGGATGTCTCACTC-3 ′
SP5 primer for 3'RACE	5′- GAGAATGTTGGGAATCATCAGCC-3 ′
Vector construction	
AAV-NIS V1 F	5′- ATATCCGGACAAAGGGAACGCAGTGAGTG-3′
AAV-NIS V1 R	5′- CTAGCGGCCGCTATGCATGCTGTTTATTTTAG -3′
AAV-NIS V2 F	5′- ATACCGCGGTCACCAAGACGGGCAAATC-3′
AAV-NIS V2 R	5′- GCGCTCGAGTAAGAAGTGGTCAAGATTT-3′
AAV-Elf1 OE F	5′- TAGAAGCTTGCCACCATGGCTGCTGTTGTCCA-3′
AAV-Elf1 OE R	5′- GCGCTCGAGTTAAAAAGAGTTGGGCTCTAG-3′
AAV-Fus OE F	5′- TAGAAGCTTGCCACCATGGCTTCAAACGACTA-3′
AAV-Fus OE R	5′- GCACTCGAGCTAATATGGCCTCTCCCT-3′
AAV-shElf1 F	5′- GATCCGATTCTAAAGCCGTGTCTAGAAGCTTGTAGACACGGCTTTAGAATCTTTTTTT-3′
AAV-shElf1 R	5′- CTAGAAAAAAAGATTCTAAAGCCGTGTCTACAAGCTTCTAGACACGGCTTTAGAATCG-3′
AAV-shFus F	5′- GATCCGTGGAGGTTATGGTCAACAGAAGCTTGTGTTGACCATAACCTCCACTTTTTTT-3′
AAV-shFus R	5′- CTAGAAAAAAAGTGGAGGTTATGGTCAACACAAGCTTCTGTTGACCATAACCTCCACG-3′
pGL3-NIS F	5′- ATAGGTACCAGCGCACAGAAGGAACTGAT -3′
pGL3-NIS R	5′- CGCAAGCTTCTTTGTGATTTGCCCGTCTT -3′
pGL3-Ccl2 F	5′- CGCGGTACCGTGAGACATTAAATCTCATG -3′
pGL3-Ccl2 R	5′- GATCTCGAGACTTCTGGCTGCTCTGAGGCAGC -3′
Single cell RT-PCR	
mNIS V1 F	5′- GGAGAGAGACAAGGGGAAGG-3′
mNIS V1 R	5′- TGTCAGTGACGTAGCCAAGC-3′
mNIS V2 F	5′- AGCTTGGGAAGCAGATTTGA-3′
mNIS V2 R	5′- TGTCTTCACACTGGCAGGAG-3′
mCcl2 F	5′- AGCACCAGCCAACTCTCACT-3′
mCcl2 R	5′- CGTTAACTGCATCTGGCTGA-3′
mFus F	5′- GGCTACTCCCAACAGAGCAG-3′
mFus R	5′- ATATCCCTGGGGAGCTGACT-3′
mRbfox3(NeuN) F	5′- AGCCTGGGAACCCATATGCC-3′
mRbfox3(NeuN) R	5′- CATCCTGATACACGACCGCT-3′
mGAPDH F	5′- GGTGAAGGTCGGTGTGAACG-3′
mGAPDH R	5′- CTCGCTCCTGGAAGATGGTG-3′
ChIP-PCR	
ChIP-NIS F	5′- CACACCCAGAGAATCCACCT-3′
ChIP-NIS R	5′- CTTACCCTGTTGGCTTGCAT-3′
ChIP-Ccl2F	5′- TCTGGTAACCACCAAGTGGAG-3′
ChIP-Ccl2R	5′- CTTTCACTGAAATAAAAGGAAGTC-3′
siRNA	
si-NIS -S	5′- GCAUAGUUGGGAAGGAGAATT-3′
si-NIS -AS	5′- UUCUCCUUCCCAACUAUGCTT-3′
si-NIS V1-S	5′- AGACUGGGCUUGUAGCAGATT-3′
si-NIS V1-AS	5′- UCUGCUACAAGCCCAGUCUGC-3′
si-NIS V2-S	5′- GGAUCUCCAUUGUGCGAGATT-3′
si-NIS V2-AS	5′- UCUCGCACAAUGGAGAUCCCA-3′
si-Ccl2-S	5′- AUCGGAACCAAAUGAGATCTT-3′
si-Ccl2-AS	5′- AAGAUCUCAUUUGGUUCCGAU-3′
si-Elf1-S	5′- CUAAUCCACAUGUGAGAACA-3′
si-Elf1-AS	5′- UGUUCUCACAUGUGGAUUAGCA-3′
si-Fus-S	5′- AUUCUAAAGCUGCAAACCCCA -3′
si-Fus-AS	5′- UGGGGUUUGCAGCUUUAGAAU-3′
RIP-PCR	
RIP-NIS-V1 F	5′- GAGGAGAGAGACAAGGGGAAG-3′
RIP-NIS-V1 R	5′- GGTGTCAGTGACGTAGCCAAG-3′
RIP-NIS-V2 F	5′- GGAAGCAGATTTGAAGCTACATG-3′
RIP-NIS-V2 R	5′- CATCTGTCTTCACACTGGCAGGAG-3′

RT: Reverse-transcription; F, Forward; R, Reverse.
